# Comparison of Lipids and Volatile Compounds in Dezhou Donkey Meat with High and Low Intramuscular Fat Content

**DOI:** 10.3390/foods12173269

**Published:** 2023-08-31

**Authors:** Qingshan Ma, Xiyan Kou, Youyou Yang, Yunshuang Yue, Weihai Xing, Xiaohui Feng, Guiqin Liu, Changfa Wang, Yan Li

**Affiliations:** 1 School of Agricultural Science and Engineering, Liaocheng University, Liaocheng 252000, China; horsegreenhill@163.com (Q.M.); kxy19990113@163.com (X.K.); guiqinliu@lcu.edu.cn (G.L.); wangcf1967@163.com (C.W.); 2 State Key Laboratory of Animal Nutrition and Feeding, Institute of Animal Sciences of Chinese Academy of Agricultural Sciences, Beijing 100193, China; yangyouyou@caas.cn (Y.Y.); oceanxwh@foxmail.com (W.X.); fengxiaohui@caas.cn (X.F.); 3 College of Food Science and Nutritional Engineering, China Agricultural University, Beijing 100083, China; daisy_1022@126.com

**Keywords:** comparison, donkey, intramuscular fat, lipids, volatile compounds

## Abstract

The intramuscular fat (IMF) content is considered an important factor for assessing meat quality, and is highly related to meat flavor. However, in donkey meat, the influences of IMF content on lipid and volatile profiles remain unclear. Thus, we conducted lipidomic and volatilomic investigations on high- and low-IMF samples from donkey *longissimus dorsi* muscle. When the IMF level increased, the monounsaturated fatty acid (especially oleic acid) content significantly increased but the saturated fatty acid content decreased (*p* < 0.05). Twenty-nine of 876 lipids showed significant differences between the two groups. Volatile profiles from differential IMF content samples were also distinct. Five differential volatile odorants were identified in the two groups: 2-acetyl-2-thiazoline, octanal, 2-pentylfuran, pentanal, and 1-(2-pyridinyl) ethanone. Additionally, strong correlations were found between differential fatty acids and lipids with differential odorants. Thus, the difference in volatile odorants may result from the change in the fatty acid composition and lipid profiles induced by different IMF contents, highlighting the urgent need to increase IMF levels in donkey meat.

## 1. Introduction

As living standards have improved in recent years, consumers’ interest in meat products that are healthy and nutritious has increased. In particular, donkey meat is a healthy food choice, as the meat is very nutritious, contains high levels of protein, is low in fat and cholesterol, and contains a high ratio of polyunsaturated fatty acids [1]. However, consumers generally believe donkey meat is tough [2], which may affect its reputation and acceptance. It is well established that intramuscular fat (IMF) content is positively related to the tenderness and flavor [3,4]. Therefore, it is very important to improve the level of IMF in donkey meat to meet the growing demands of consumers and promote the consumption of donkey meat.

When choosing meat products, consumers also strongly consider their flavor. The identification of volatile compounds in meat has recently received much attention, as these compounds greatly determine the flavor and/or odor of meat products [5]. Studies have shown that the IMF level exhibited an effect on the volatile flavor substances in meat, which included lipid oxidation/degradation products (such as aldehydes, alcohols, and ketones) and Maillard reaction products [6,7]. Fatty acid profiles of IMF also have significant influence on the formation of flavor [8]. Furthermore, the fatty acid composition in meat must be considered as it is an important factor for human health. Additionally, lipid species in IMF not only act as solvents for volatile compounds in meat but also produce special flavors, which are important flavor precursors [9]. Regarding this topic, Liu et al. [10] noted that 61 differential lipid subclasses (particularly phosphatidylcholine (PC) and phosphatidylethanolamine (PE)) may be responsible for the production of volatile aroma compounds (such as hexanal, heptanal, and 1-octen-3-ol) in roasted lamb after various cooking times. Moreover, lipids also contribute to food texture (such as the texture of food during mastication) and thus lead to greater acceptance by the consumer. Thus, the lipid profiles of donkey meat with varying IMF levels should be analyzed and identified to better discover the flavor precursors and enhance the meat flavor and quality.

China is the world’s greatest producer of donkey meat (183,755 tons/year), followed by Niger (9946 tons/year) [11]. The Dezhou donkey is a well-known breed in China, mainly owing to its remarkable characteristics, including huge size, muscular body, and best skin quality. In our previous studies, we observed differences in the volatile compounds (VOCs) of meat from two donkey strains (SanFen and WuTou) [12]. However, the impact of IMF level on the flavor compounds and precursors of Dezhou donkey meat remains unclear. Therefore, we utilized a lipidomic approach combined with volatilomic analyses to characterize the lipid and volatile profiles and their variations sampled with high- and low-IMF levels, and to further evaluate the relationship between lipid profiles and volatile compounds in donkey meat. The present study could provide a better understanding of IMF deposition affecting the flavor of donkey meat.

## 2. Materials and Methods

### 2.1. Sample Collection

Eighty healthy male Dezhou donkeys (carcass weight: 136.94 ± 2.08 kg) approximately 2.5 years of age were obtained from the same farm and slaughtered at a donkey slaughterhouse (Dong’a Tianlong Food Company, Liaocheng, China) according to international standards (CAC/RCP 41-1993 and ISO/TS 34700: 2016). Soybean straw diets were fed ad libitum in addition to a commercial concentrate diet (Hekangyuan Group Co., Ltd., Jinan, China), and donkeys were fed twice daily at 07:00 and 19:00. A total of approximately 20 g of *longissimus dorsi* (LD) muscle samples (between ribs 17 and 18) were collected and divided from two parts after sacrifice, washed with sterile saline, frozen in liquid nitrogen, and subjected to measurement of the chemical composition, lipids, and volatile compounds. All frozen samples were then stored at −80 °C. All procedures performed in the present work were approved by the Liaocheng University Animal Care Committee (No. 2022121601).

### 2.2. Measurement of the Chemical Composition

The IMF contents in the donkey LD muscle samples were measured by the Soxhlet extraction method following the Chinese National Standard (GB/T 6433.2006), and expressed as a percentage of wet meat weight. Then, samples were divided into two groups according to IMF content: low 10% IMF (L-IMF, *n* = 8) and high 10% IMF (H-IMF, *n* = 8) groups (Table S1). 

Additionally, the fatty acid profile of donkey meat was detected through gas chromatography (GC; 6890N, Agilent) with a DB-23 column at the Ministry of Agriculture Feed Industry Centre of China. Briefly, samples were put into a hydrolysis tube, and the internal standard of 4 mL of chloroacetyl methanol solution (1:10) and 1 mL of C11:0 methyl ester were added. Then, the samples were mixed with 1 mL of *n*-hexane in a water bath at 80 °C for 2.0 h. After cooling, 5 mL of potassium carbonate solution (7%) was added and vortexed for 1 min, and then centrifuged at 1000 r/min for 5 min. Finally, the samples were filtered into a sample vial for GC analysis, and injected with a volume of 1 µL. Fatty acids were identified by comparisons of their retention time with those of fatty acid standards and expressed as percentages of total fatty acids.

### 2.3. Lipidomic Analysis

Lipids were extracted and collected from donkey meat according to a prior method with a slight modification [13]. In brief, the LD muscles were suspended in the mixture of chloroform and methanol with the volumetric ratio of two to one and the obtained suspension was then mixed with deionized water. After vortex mixing, the solid phase was separated from the suspension via centrifugation at 4 °C and the liquid phase was collected and dried under nitrogen to form pellet, which was resuspended in the chloroform/methanol (1:1, *v*/*v*) before LC-MS analysis. To monitor the quality and stability of data assayed, quality control samples were obtained by mixing the equal volume of each sample, followed by the injection at every seven samples.

Lipid sample was separated and analyzed on a modular 1290 infinity HPLC system (Agilent, Waldbronn, Germany) equipped on a Waters Acquity UPLC HSS T3 (Waters, Milford, USA). The mobile phase was composed of mobile phase A (acetonitrile/water, 60:40, *v*/*v*) and mobile phase B (isopropanol/acetonitrile, 90:10, *v*/*v*), both of which contained 10 mM ammonium formate. The gradient program was set as follows: 0–1.0 min, 40% B; 1.0–9.0 min, 100% B; and then 10.20–13 min, 60% B. The total run time was 13 min with the flow rate of 300 μL/min. The column temperature was 50 °C and the injection volume of the sample was 2 μL. Mass spectrometry (MS) was performed in both positive- and negative-ion modes. The MS parameters for extracting lipids were performed as follows: ion spray voltage of the positive and negative modes, 5.0 kV and 4.5 kV, respectively; temperature of turbo source gun, 500 °C; and curtain gas (CUR), 35 psi.

The lipids were identified and screened using MExplorer (version 1.0.158(158)). Mass tolerance was 10 ppm and 5 ppm for fragments and precursors, respectively. The relative quantifications of the lipids identified in this work were performed using their relative peak areas.

### 2.4. Volatile Compounds Analysis

The pretreatment, extraction, and analysis of volatile compounds from LD muscle samples were performed using headspace solid-phase micro extraction and gas chromatography-mass spectrometry (HS-SPME-GCMS) [6,14]. Following incubation for 20 min in water at 55 °C, the samples were extracted for 40 min at 55 °C using 50/30 μm DVB/CAR/PDMS (Supelco, Inc., Bellefonte, PA, USA), and then desorbed at 250 °C for 3 min into the GC inlet.

Volatile components were separated and analyzed via a GC-MS system (Thermo Fisher Scientific, Austin, TX, USA) equipped with a VF WAX capillary column (Agilent, Santa Clara, CA, USA). Helium (99.9999%) was the carrier gas under a constant flow rate of 1.0 mL/min. The column oven temperature program was first set to 40 °C for 2 min, then raised to 230 °C at a rate of 4 °C/min and maintained for 5 min. The MS Electron ionization was carried out at 70 eV electron energy. The MS ion source was 280 °C, whereas the MS transfer line was 250 °C. An Orbitrap MS at 60,000 resolution was used for full scan MS. Moreover, the scanning range was from 30 *m*/*z* to 400 *m*/*z*.

Volatile compounds were identified and confirmed by comparisons of their retention indices and mass spectra with the corresponding database of NIST v2.3 and Wiley libraries built with authentic reference standards. These compounds were then semiquantified by utilizing an internal standard (2-methyl-3-heptanone). To further analyze the contribution of aroma compounds, the odor-active values (OAVs) of aroma compounds were calculated using their semi-quantification concentration divided by the corresponding threshold value [15]. Finally, the volatile components with OAV > 1 were generally defined as key flavor substances.

### 2.5. Data Analysis

Student’s *t*-test (normal distribution data) or Wilcoxon rank sum test (non-normal distribution data) were carried out to run the statistical analyses with SPSS software (version 22). Data were expressed as the mean ± standard error (SE), and *p* < 0.05 was regarded as statistical significance. Significantly differential lipids were screened by following these criteria: false discovery rate (FDR) < 0.05, fold-change (FC) > 2 or < 0.5, and variable importance in projection (VIP) > 1. Partial least-squares discriminant analysis (PLS-DA), volcano plot, heatmaps, and VIP scores were performed and visualized using MetaboAnalyst 5.0. Spearman correlation method analyses among the discriminative key volatile compounds and differential fatty acids and lipids were conducted using OriginPro 2021. Additionally, the GraphPad Prism 8.0 was carried out to build the bar charts.

## 3. Results and Discussion

### 3.1. Comparison of Lipid Profiles

**Figure 1:**
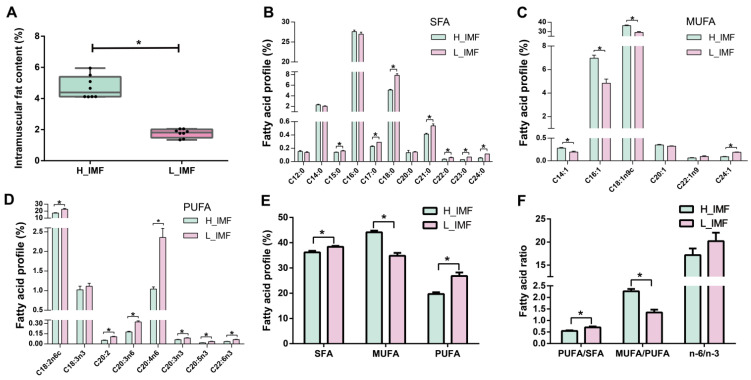
Intramuscular fat content and fatty acid profile in H_IMF and L_IMF. (**A**) Intramuscular fat content. (**B**) SFAs. (**C**) MUFAs. (**D**) PUFAs. (**E**) Total SFAs, MUFAs and PUFAs. (**F**) PUFA/SFA, MUFA/PUFA and n-6/n-3 PUFA. L_IMF, low intramuscular fat; H_IMF, high intramuscular fat; SFA, saturated fatty acid; MUFA, monounsaturated fatty acid; PUFA, polyunsaturated fatty acid. * *p* < 0.05.

Based on the IMF content, extreme samples were screened and divided into two groups: the L-IMF and H-IMF groups. As expected, significantly higher accumulation of IMF was observed in the H-IMF group compared with the L-IMF group (4.71% versus 1.77%; *p* < 0.01; Figure 1A). Compared to lamb or beef, donkey meat has a low percentage of IMF [16,17]. Additionally, previous studies have shown that palatability is noticeably diminished when the fat content drops to as low as 3% [18]. This indicates that higher levels of intramuscular fat (IMF) in this study could enhance consumer acceptance of donkey meat.

To better explore the changes in lipid profiles in donkey LD muscle with different IMF contents, lipid profiles were assessed and analyzed by the following approaches: targeted free fatty acid assay and untargeted lipidomic analysis. Three major fatty acids identified in these meat samples were C18:2n-6, C18:1n-9, and C16:0. Furthermore, the most abundant saturated fatty acid (SFA) and monounsaturated fatty acid (MUFA) as well as polyunsaturated fatty acid (PUFA) in donkey meat were C16:0, C18:1n-9, and C18:2n-6, respectively (Figure 1B–D). The overall fatty acid proportion of the donkey LD muscle was similar to that found in donkey meat in prior studies [19,20]. Specifically, for the SFAs, we found that the proportions of C15:0, C17:0, C18:0, C21:0, C22:0, C23:0 and C24:0 as well as the total SFA were significantly lower in H-IMF than in L-IMF (*p* < 0.05; Figure 1B,E). Compared with the SFAs, the IMF level displayed its particular roles in the MUFA content of the donkey LD muscle. Oleic acid (C18:1n-9) was the most abundant MUFA among all samples, followed by palmitoleic acid (C16:1), and the contents of these MUFAs were significantly higher in H-IMF samples than L-IMF samples (*p* < 0.05; Figure 1C). Regarding the PUFAs, almost all PUFAs (except linolenic acid (C18:3n-3)) and the total PUFA content were reduced significantly in H-IMF (*p* < 0.05; Figure 1D,E). These data suggested that MUFAs (particularly oleic acid) might contribute to IMF deposition. The results also suggested that increases in the IMF level in the donkey LD muscle were accompanied by increases in the total MUFA proportion and decreases in the total SFA proportion, which concurred with the findings obtained by Joo et al. [21] and Gotoh et al. [22] with cattle. However, both SFAs and MUFAs increased with increasing IMF level in lamb meat [23] and pork [24]. The discriminatory results obtained with meats from various animals may result from the differences in energy metabolism and mitochondrial function in muscle fibers. The PUFA/SFA ratio was observably lower in H-IMF samples than in L-IMF samples (*p* < 0.05), but the values found for these two groups were close to or slightly higher than the upper limit (≥0.45–0.7) according to health authorities’ guidelines (Figure 1F) [25]. In addition to the concentration related to the n-6 PUFA and n-3 PUFA series, the ratio of n-6 to n-3 PUFA also has a significant effect on human health [26], but the value of this ratio did not differ between the two groups in the present study (Figure 1F). Considering the MUFA effect, its main fatty acid is oleic acid, which is strongly associated with improvements in human health including decreased blood pressure, controlled glycaemia, and improved lipid metabolism [27,28]. Furthermore, a high level of oleic acid and a high MUFA/PUFA ratio could promote the production of pleasant flavor in cooked ham [29] and improve pork quality [30]. Additionally, atherogenic and hypocholesterolemic/hypercholesterolemic indices did not differ noticeably between two groups, whereas the H-IMF group had a lower thrombogenic index than the L-IMF group (Figure S1). These results obtained in this study suggest that an improvement in IMF content does not exert adverse effects on human health or lower the nutritional values of donkey meat based on the fatty acid profiles. This observation was also supported by the results of a study of pork [31]. Additionally, these data also showed that the change in fatty acid composition may further affect the meat flavor and quality [8,29,30].

**Figure 2:**
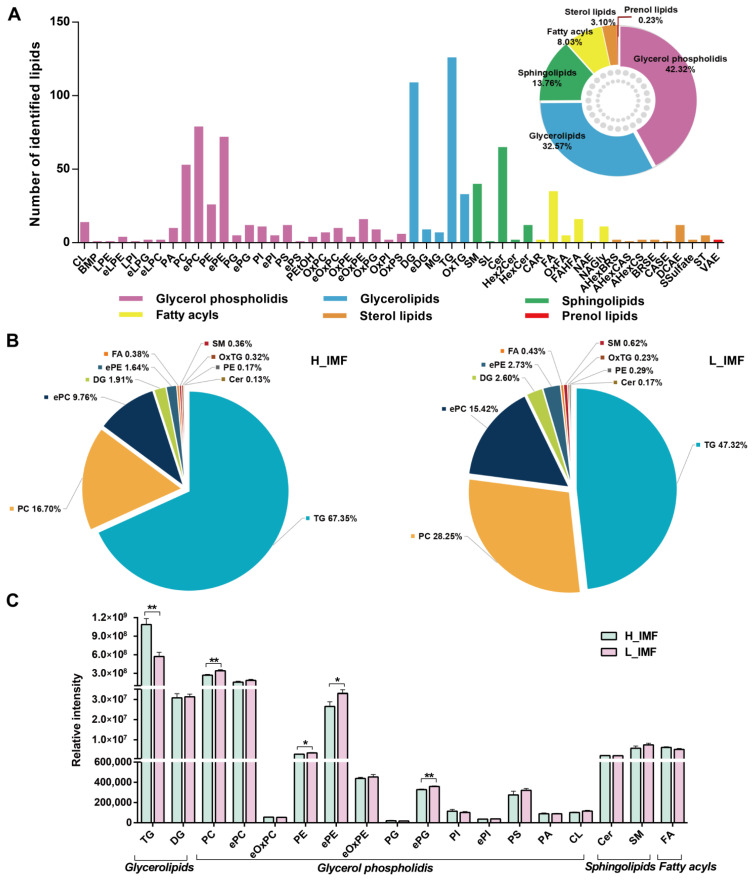
Overview of lipid categories and subclasses. (**A**) Numbers of lipids identified in 51 lipid subclasses and percentages of the numbers of 6 lipid categories. (**B**) Comparison of the percentages of lipid subclasses between H_IMF and L_IMF. (**C**) Comparison of the contents of each lipid subclass between H_IMF and L_IMF. Abbreviations: L_IMF, low intramuscular fat; H_IMF, high intramuscular fat; CL, cardiolipin; BMP, bismonoacylglycerophosphate; LPE, lysophosphatidylethanolamine; eLPE, ether-linked lysophosphatidylethanolamine; LPI, lysophosphatidylinositol; eLPG, ether-linked lysophosphatidylglycerol; PA, phosphatidic acid; PC, phosphatidylcholines; ePC, ether-linked phosphatidylcholine; eLPC, ether-linked lysophosphatidylcholine; ePE, ether linked phosphatidylethanolamine; PE, phosphatidylethanolamine; PG, phosphatidyl glycerol; ePG, ether-linked phosphatidylglycerol; PI, phosphatidylinositol; ePI, ether-linked phosphatidylinositol; PS, phosphatidylserine; ePS, ether-linked phosphatidylserine; PEtOH, phosphatidylethanol; OxPC, oxidized phosphatidylcholine; eOxPC, ether linked oxidized phosphatidylcholine; eOxPE, ether linked oxidized phosphatidylethanolamine; OxPG, oxidized phosphatidylglycerol; OxPE, oxidized phosphatidylethanolamine; OxPS, Oxidized phosphatidylserine; OxPI, oxidized phosphatidylinositol; DG, diacylglycerol; eDG, ether-linked diacylglycerol; MG, monoacylglycerol; OxTG, oxidized triacylglycerols; TG, triacylglycerols; SM, sphingomyelin; SL, sulfonolipid; Cer, ceramides; Hex2Cer, dihexosylceramide; HexCer, hexosylceramide; FA, free fatty acid; OxFA, oxidized fatty acid; CAR, acylcarnitine; FAHFA, fatty acid ester of hydroxyl fatty acid; NAE, N-acyl ethanolamines; NAGly, N-acyl glycine; AHexBRS, acylhexosyl brassicasterol; AHexCAS, acylhexosyl campesterol; AHexCS, acylhexosyl cholesterol; BRSE, brassicasterol ester; CASE, campesterol ester; DCAE, esterified deoxycholic acid; SSulfate, sterol sulfate; VAE, vitamin A fatty acid ester. * *p* < 0.05; ** *p* < 0.01.

Lipidomic profiling of donkey meat with different IMF contents was performed by UPLC–MS. In all, 876 lipids were successfully discovered and characterized in positive and negative ion modes. Lipids were classified into 51 subclasses, including 126 TGs, 109 DGs, 79 ePCs, 72 ePEs, 65 Cers, 53 PCs, 40 SMs, 35 FAs, 33 OxTGs, 26 PEs, 16 FAHFAs, 16 eOxPEs, 14 CLs, and others (Figure 2A and Table S2). Furthermore, all lipids were grouped into six major categories, namely, 369 (42.32%) glycerol phospholipids (GPs), 284 (32.57%) glycerolipids (GLs), 122 (13.76%) sphingolipids (SPs), 70 (8.03%) fatty acyls (FAs), 27 (3.10%) sterol lipids (STs), and two (0.23%) prenol lipids (PRs) (Figure 2A). The relative contents of lipid classes for the two groups are given in Figure 2B,C. The H-IMF samples contained the highest relative abundance of the TG lipid class, accounting for 67.35%, followed by PC (16.70%), ePC (9.76%), and DG (1.91%), and a similar trend was observed in the L-IMF groups. This finding was consistent with the results obtained in a prior study, which found that the overall lipid profiles of lambs exhibit similar distributions in the HIMF and LIMF groups [6]. Past studies have noted that lipid profiles could be affected by the IMF content [6,32]. As indicated in Figure 2C, the H-IMF group had significantly increased TG, and lower PC, PE, ePE, and ePG levels than the L-IMF group (*p* < 0.05); however, the other lipid classes were unaffected (*p* >0.05), indicating that glycerides (mainly TG content) in H-IMF samples may contribute to their high IMF level. These findings are similar to the results previously obtained by Hou et al. [32] and Li et al. [33], who demonstrated that Laiwu pork (high IMF content) contained more triglycerides than Yorkshire pork (low IMF content), and decreases in the PE and PC levels were observed with increasing the IMF content.

**Figure 3:**
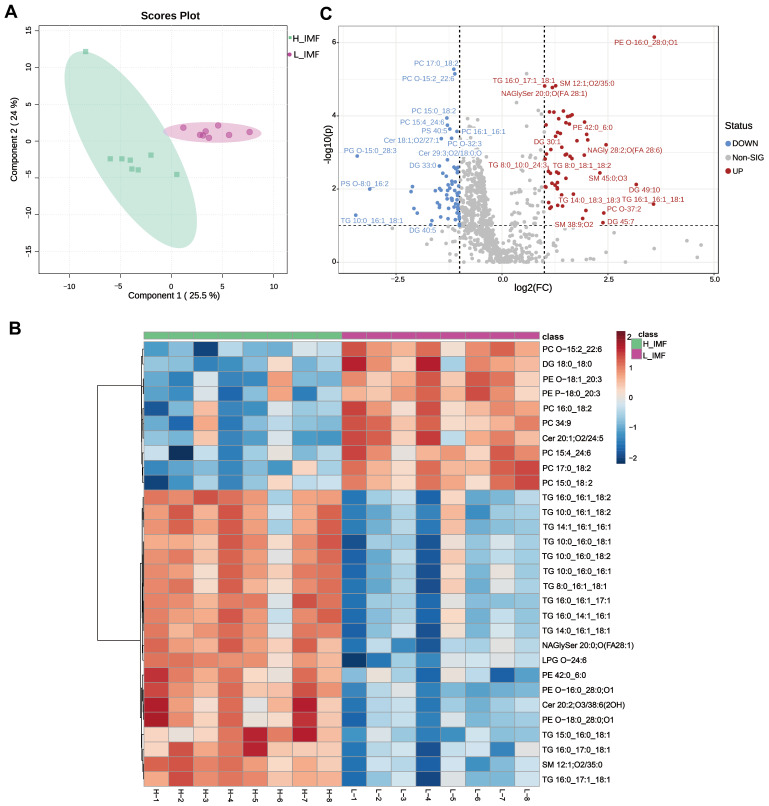
Lipid profiles of donkey meat from the H_IMF and L_IMF groups. (**A**) PLS-DA score plots. (**B**) Heatmap analysis. (**C**) Volcano plot. L_IMF, low intramuscular fat; H_IMF, high intramuscular fat; PLS-DA, partial least squares-discriminant analysis.

To further demonstrate the difference between the lipid profiles of the H-IMF and L-IMF samples, lipidomics were then analyzed by multivariate statistics. We generated the PLS-DA plots and found that the two groups could be separated clearly without any overlap (Figure 3A), indicating that the PLS-DA model can be used effectively to filter differential lipids between groups [34]. Then, 105 significantly differential lipids were identified and selected with the criteria of VIP scores > 1 and FDR < 0.05 (Table S3). A heatmap (TOP 30) was generated to visually compare the significantly different lipids of each lipid subclass, as shown in Figure 3B. As shown in the volcano plots in Figure 3C, 124 significantly different lipids were identified and filtered with fold change > 2 or <0.5, and 58 and 66 lipids were up-regulated and down-regulated, respectively, in H-IMF compared to L-IMF. Afterward, differential lipids were identified and screened under these criteria of volcano plot (FC > 2 or <0.5) and VIP (VIP > 1.0, FDR < 0.01). Subsequently, 29 of these lipids, eight down-regulated and 21 up-regulated, were kept for further correlation analysis (Table S4). The outcomes indicate that the most differential molecular makeups of PCs enriched in PUFAs are more abundant in L-IMF, which is in accordance with the aforementioned change in the fatty acid composition observed in the study subjects. Moreover, TGs and phospholipids could offer the aroma and flavor of meat, owing to their high content of unsaturated fatty acids (more prone to lipid oxidation) [35], indicating that differential lipids could affect the meat flavor.

Taken together, these findings indicate that lipid profiles of meat with different IMF contents were distinct, which may influence the flavor production of donkey meat.

### 3.2. Changes in Aroma Compounds

**Figure 4:**
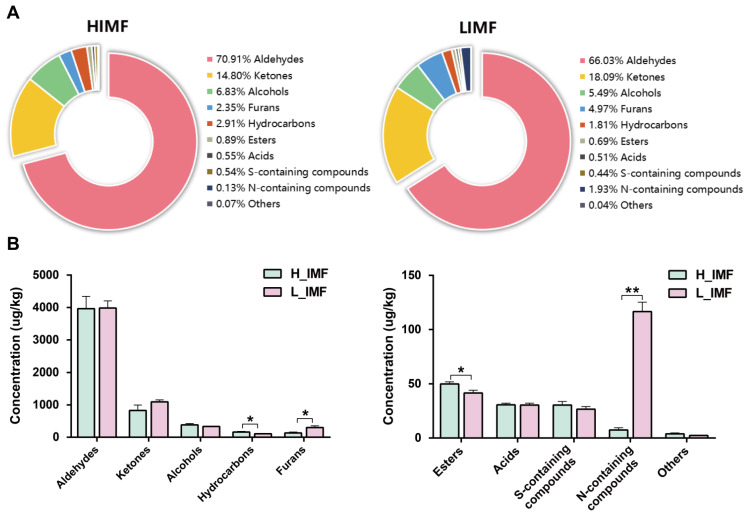
Overview of volatile categories between the H_IMF and L_IMF groups. Percentages (**A**) and concentrations (**B**) of volatile categories identified between the two groups. L_IMF, low intramuscular fat; H_IMF, high intramuscular fat. * *p* < 0.05; ** *p* < 0.01.

**Figure 5:**
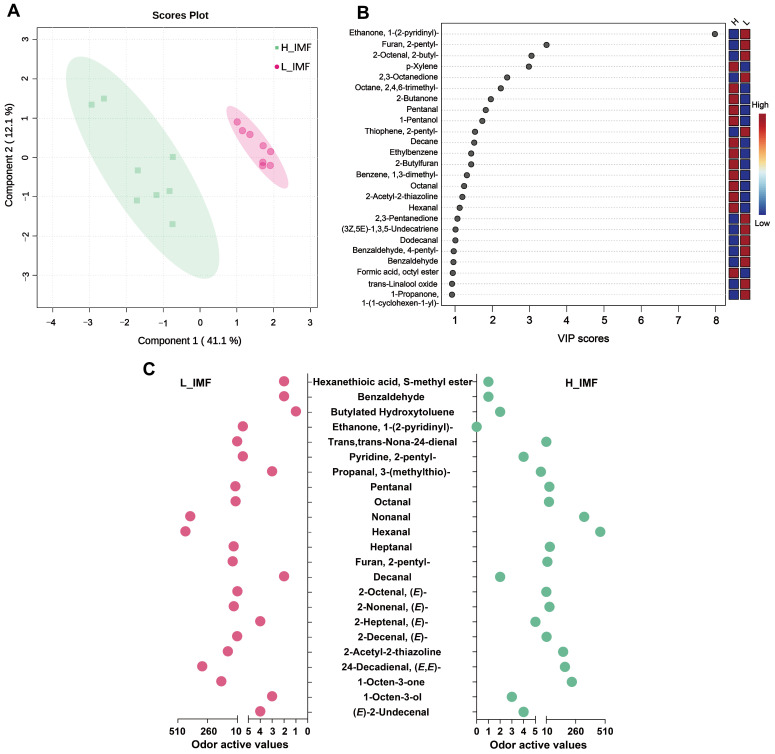
Volatile profiles for donkeys with varied IMF content. (**A**) PLS-DA score plots. (**B**) VIP scores (top 25). Each point in the graph represents the VIP score. (**C**) Odor active values of volatile compounds. L_IMF, low intramuscular fat group; H_IMF, high intramuscular fat group; VIP, variable importance in projection; PLS-DA, partial least squares-discriminant analysis.

Volatile compounds can determine the aroma and flavor of meat to some extent. A total of 158 volatile compounds were analyzed and identified in the H-IMF and L-IMF groups using HS-SPME-GCMS (Table S5). These compounds are grouped into ten main classes, including 34 aldehydes, 29 ketones, 11 alcohols, 8 furans, 24 hydrocarbons, 25 esters, 9 acids, 8 S-containing compounds, 6 N-containing compounds and 4 others (Table S5 and Figure 4A). Among the ten substances in donkey meat observed in this study, aldehydes were present at the highest levels, accounting for 70% and 66% in H-IMF and L-IMF, respectively (Figure 4A), indicating that aldehydes might play a key role in the flavor production of donkey meat. Similarly, a number of studies reported that aldehydes were more abundant and the most important aroma compounds owing to their low odor threshold for meat [20,36]. The concentrations of hydrocarbons, furans, N-containing compounds, and esters were affected by IMF level (*p* < 0.05, Figure 4B). As given in Figure 5A, the score plots from the PLS-DA showed an obvious separation between groups, indicating a difference in volatile flavor compounds between the two groups. It has been shown that the volatile aroma profiles could be affected by the IMF content [6,37], but there are also the differential effects on each volatile substance mainly connecting with their lipophilicity and precursors [6].

In brief, Figure 5B shows a VIP score plot of volatile compounds with a VIP value > 1.0 (top 25). Table S5 also shows 16 volatile compounds with significant differences between the two groups (*p* < 0.05, VIP > 1.0). These differential compounds include four aldehydes (2-octenal,2-butyl-, dodecanal, octanal and pentanal), one ketone (2-butanone), one alcohol (1-pentanol), one furan (pentylfuran), two sulfur-containing compounds (thiophene, 2-pentyl- and 2-acetyl-2-thiazoline), one nitrogen-containing compound (ethanone, 1-(2-pyridinyl)-), and six hydrocarbons ((3Z,5E)-1,3,5-Undecatriene, benzene,1,3-dimethyl-, decane, ethylbenzene, octane,2,4,6-trimethyl-, and *p*-xylene). Notably, hydrocarbons are the most differential volatile compounds, but they do not contribute to the flavor of donkey meat, owing to low concentrations and high aroma threshold [38]. Thus, to further evaluate the aroma contribution of volatile compounds in these samples, the odor activity values (OAVs) are determined, as listed in Table S6. As presented in Figure 5C and Table S7, 23 odor-active volatile compounds with OAV > 1.0 were discovered in the H-IMF and L-IMF groups, indicating that they may provide a powerful contribution to the aroma and flavor of donkey meat. Among them, hexanal had the highest OAV in the H-IMF samples, followed by nonanal, 1-octen-3-one, 2,4-decadienal (*E*,*E*) and 2-acetyl-2-thiazoline, all of which had OAVs > 100, and a similar trend was observed in the L-IMF samples. This finding corresponded with those obtained in previous results, and some aldehydes (e.g., hexanal) and alcohols (e.g.,1-octen-3-ol) were the predominant volatile aroma compounds in donkey meat [20] and roasted mutton [10].

Combined with the data for differential volatile compounds, significant differences in five odor-active volatile compounds (with *p* < 0.05, VIP score > 1.0 and OAV > 1.0), namely, pentanal, 2-acetyl-2-thiazoline, 2-pentylfuran, and 1-(2-pyridinyl) ethanone and octanal, were observed between the two groups, indicating that these compounds could be used as aroma markers to differentiate the two groups. These compounds were also retained for further investigation (Table S8). Among them, three compounds (2-pentylfuran, pentanal and octanal) were mainly derived from lipid oxidation and degradation. For instance, 2-pentylfuran exhibits high flavor activity (musty, beany, butter) due to its low threshold value, which could be produced from the PUFAs oxidation [39,40]. The 2-pentylfuran content was significantly lower in H-IMF than in L-IMF. In contrast, significantly higher contents of octanal and pentanal were observed in the LD muscle of the H-IMF group compared to the L-IMF group, which is similar to the data obtained for pork bellies [37]. Octanal can confer pleasant flavors, such as meat-like, green, and citrus-like notes, which are mainly derived from oleic acid autoxidation [41]. As previously mentioned, apart from the volatile molecules derived from lipid oxidation/degradation, the compounds produced by Maillard reaction were also affected by the IMF level [6,7]. This finding was also confirmed in our study. For example, 2-acetyl-2-thiazoline, which is mainly generated by the Maillard reaction between cysteine/cysteamine and reducing sugars [42,43], imparts an attractive aroma with a nutty, roasted meaty-like flavor and popcorn-like aroma [44], and is an important aroma compound in cooked beef [45] and dry aged beef loin [46]. This compound was found at significantly higher levels in the H-IMF group than in the L-IMF group. A similar outcome was observed by Li et al. [6]; the 2-acetyl-2-thiazoline concentration exhibited an increasing trend (8.03 μg/kg to 9.39 μg/kg) with increasing IMF level (2.24% to 5.17%), but these values were lower than those detected in our study (18.48 μg/kg and 10.9 μg/kg were detected in H-IMF and L-IMF, respectively). Furthermore, we found that the OAV of 2-acetyl-2-thiazoline was highest among the five differential aroma compounds, suggesting that this compound is an important aroma compound of donkey meat and that improving the IMF content might enhance the production of this compound. Taken together, the results indicate that the volatile profiles of meat with different IMF contents are distinct.

### 3.3. Correlation Analysis of Fatty Acids, Lipidomics Data, and Volatile Compounds

**Figure 6:**
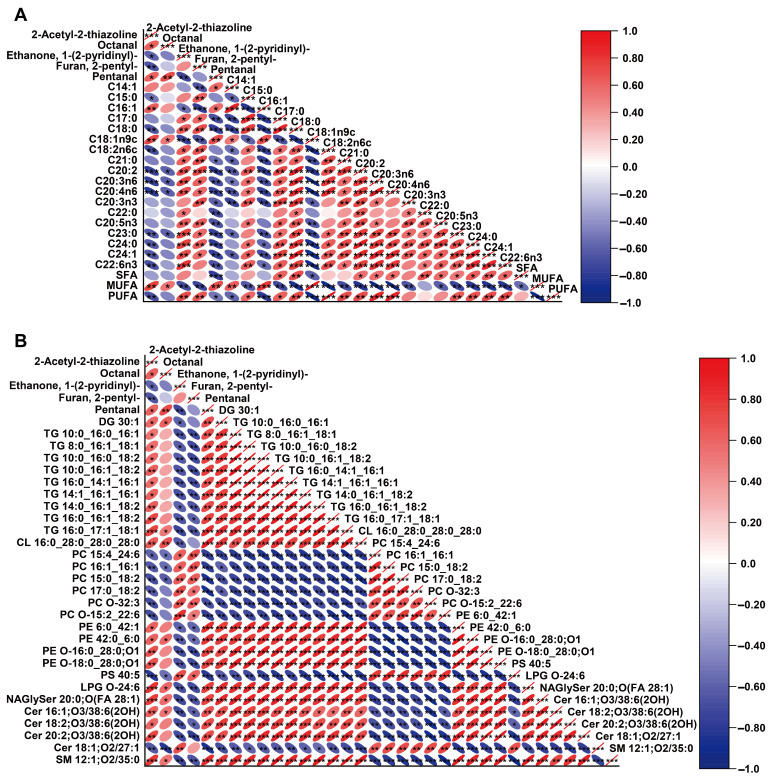
Correlation map for the differential volatile aroma compounds with differential fatty acids (**A**) and screened lipid markers (**B**). Red and blue ellipses indicate positive and negative correlations, respectively. The darker color of the graph represents the stronger correlation, * *p* < 0.05; ** *p* < 0.01; *** *p* < 0.001.

The correlations among fatty acids, lipidomics data, and volatile compounds were analyzed to assess the contribution and effect of each lipid compound on the flavor of donkey meat with different IMF contents. The correlation heatmaps are shown in Figure 6. Five discriminative odorants were significantly correlated with the 18 differential fatty acids (*p* < 0.05; Figure 6A and Table S9). We observed that the three aroma compounds, 2-acetyl-2-thiazoline, octanal, and pentanal were positively correlated with palmitoleic acid (C16:1) and oleic acid (C18:1n9c), whereas most SFAs (e.g., C18:0)) and PUFAs (e.g., linoleic acid (C18:2n6c)) were negatively associated. In contrast, two aroma compounds, namely, 1-(2-pyridinyl) ethanone and 2-pentylfuran, were enriched in the L-IMF group and were significantly positively correlated with most SFAs and PUFAs (e.g., linoleic acid (C18:2n6c)) but negatively correlated with palmitoleic acid (C16:1) and oleic acid (C18:1n9c) contents. This result was confirmed by reports showing that the volatile compounds in meat could be affected by fat level and may be partly responsible for altering the fatty acid profiles [37]. According to a report, even a minor alteration in the fatty acid composition of meat can lead to changes in the aroma volatiles [47]. This finding also agreed with prior studies that the oxidation of oleic acid (C18:1n9c) could produce octanal [41] and pentanal [48]. Pentanal was positively related to the content of palmitoleic acid (C16:1) but negatively related to the concentration of linoleic acid (C18:2n6c) [49]. 2-Pentylfuran was also observed to have a significant positive correlation with C18:2 in pork [50]. In the current investigation, 29 differential lipids were significantly related to five discriminative odorants (*p* < 0.05; Figure 6B and Table S10). Twenty, eight, and twenty-one differential lipids, mainly belonging to the TG and PE classes, were positively correlated with 2-acetyl-2-thiazoline, octanal and pentanal, respectively. Furthermore, a total of eight and seven differential lipids mainly belonging to the PC class (such as PC15:0_18:2 and PC17:0_18:2) were positively correlated with 1-(2-pyridinyl) ethanone and 2-pentylfuran, respectively. Li et al. [51], Liu et al. [10] and Man et al. [52] found a significant relationship between differential lipids and aroma compounds in meat products, which supports the current finding. For example, Liu et al. [10] found that differential lipids (especially PC, PE and TG) were significantly related to predominant aroma compounds (including 1-octen-3-ol, hexanal, pentanal, and 2-pentylfuran) in roasted lamb at various cooking times. This result indicates that the 18 differential fatty acids and 29 lipid subclasses (mainly belonging to TG and PC) may predominantly contribute to the formation of discriminative odorants affected by the IMF levels in the donkey meat. Although the lipidomics in this study was used to detect lipid-soluble flavor precursors, the influence of the IMF content on hydrophilic metabolites in donkey meat needs to be further investigated.

## 4. Conclusions

In summary, the present study investigated the differences in donkey meat with different IMF content through lipidomic and volatilomic analysis. The MUFA proportion increased but SFA proportion decreased with increasing IMF levels in donkey meat. Lipidomic analysis showed a significant impact of IMF levels on lipid profiles. Furthermore, the integrated analysis of volatile compounds comparison and OAV results (OAV > 1.0) revealed the presence of five specific volatile compounds in donkey meat. These compounds, namely, 2-acetyl-2-thiazoline, octanal, 2-pentylfuran, pentanal, and 1-(2-pyridinyl) ethanone, not only exhibit distinct differences between the high and low IMF groups but also significantly contribute to the flavor of donkey meat.. Additionally, correlation analysis showed that fatty acids (such as oleic acid and linoleic acid) and lipid species (especially TG, PC and PE) might be involved in the production of donkey meat flavor. More work is needed to further unravel these complex relationships, especially in terms of the Maillard reaction product (e.g., 2-acetyl-2-thiazoline). This study could promote our knowledge of how the IMF level affects the meat flavor.

## Data Availability

Data will be made available on request.

## Supplementary Materials

The following supporting information can be downloaded at: https://www.mdpi.com/article/10.3390/foods12173269/s1, Figure S1: Consumer health indexes based on fatty acid concentrations between the H_IMF and L_IMF groups. AI, atherogenic index; TI, thrombogenic index; h/H, hypocholesterolemic/hypercholesterolemic; H_IMF, high intramuscular fat group; L_IMF, low intramuscular fat group. * *p* < 0.05; Table S1: Basic information on 80 donkeys; Table S2: Lipid molecular species identified in the low and high intramuscular fat groups using untargeted lipidomics (*n* = 8); Table S3: Significantly changed lipid molecules between the low and high intramuscular fat groups (*n* = 8); Table S4: Significantly changed and FC > 2/FC < 0.5 lipid molecules between the low and high intramuscular fat groups (*n* = 8); Table S5: Aroma compounds identified in donkey meats (*n* = 8); Table S6: Odor thresholds and odor active values in low and high intramuscular fat groups (*n* = 8); Table S7: Odorants (OAV > 1) in low and high intramuscular fat groups (*n* = 8); Table S8: Significantly changed odorants between the low and high intramuscular fat groups (*n* = 8); Table S9: Correlation between the fatty acids and differential odorants; Table S10: Correlation between the differential lipids and differential odorants.
