# Supplementary material for: Comparison of Lipids and Volatile Compounds in Dezhou Donkey Meat with High and Low Intramuscular Fat Content
Source: Foods. 2023 Aug 31;12(17):3269. doi: 10.3390/foods12173269 (PMC10486446; doi:10.3390/foods12173269)
Supplement: Supplementary file 1 [file foods-12-03269-s001.zip › foods-2572847-supplementary.pdf]

Supplementary information

Figure S1: Consumer health indexes based on fatty acid concentrations between the H_IMF and L_IMF groups. AI, atherogenic index; TI, thrombogenic index; h/H, hypocholesterolemic/hypercholesterolemic; H_IMF, high intramuscular fat group; L_IMF, low intramuscular fat group. *$P < 0.05$.

Table S1: Basic information on 80 donkeys.

Table S2: Lipid molecular species identified in the low and high intramuscular fat groups using untargeted lipidomics (n=8).

Table S3: Significantly changed lipid molecules between the low and high intramuscular fat groups (n=8).

Table S4: Significantly changed and FC > 2/FC < 0.5 lipid molecules between the low and high intramuscular fat groups (n=8).

Table S5: Aroma compounds identified in donkey meats (n=8).

Table S6: Odor thresholds and odor active values in low and high intramuscular fat groups (n=8).

Table S7: Odorants (OAV > 1) in low and high intramuscular fat groups (n=8).

Table S8: Significantly changed odorants between the low and high intramuscular fat groups (n=8).

Table S9: Correlation between the fatty acids and differential odorants.

Table S10: Correlation between the differential lipids and differential odorants.

Supplementary Figure

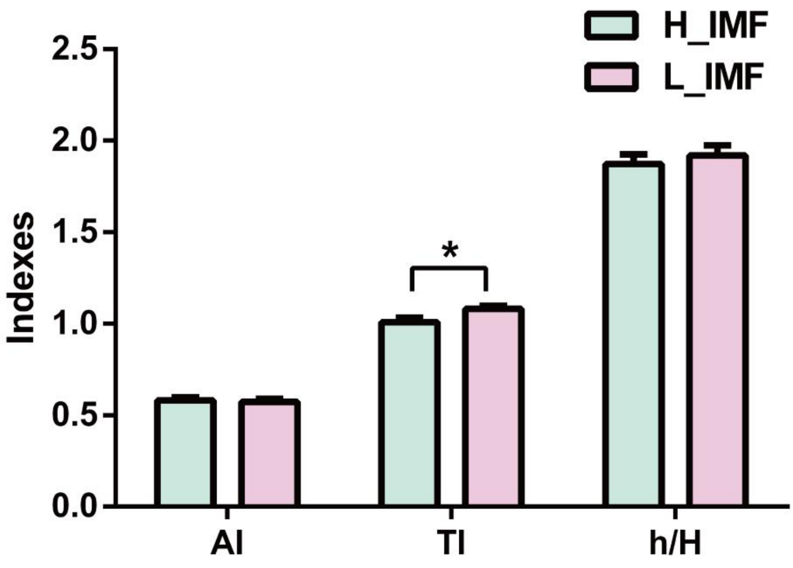

Figure S1. Consumer health indexes based on fatty acid concentrations between the H_IMF and L_IMF groups. AI, atherogenic index; TI, thrombogenic index; h/H, hypocholesterolemic/hypercholesterolemic; H_IMF, high intramuscular fat group; L_IMF, low intramuscular fat group. **P* < 0.05. Three indexes were calculated based on FA concentrations as follows:

1 Atherogenic index (AI): [C12:0 + (4*C14:0) + C16:0] / [n-6 PUFA +n-3 PUFA + MUFA] (Ulbricht & Southgate, 1991).

2 Thrombogenic index (TI): [C14:0 + C16:0 + C18:0] / [0.5*MUFA +0.5*n-6 PUFA +3*n-3 PUFA + (n-3 PUFA/n-6 PUFA)] (Ulbricht & Southgate, 1991).

3 Hypocholesterolemic / hypercholesterolemic (h/H): [C18:1 cis-9 +C18:2 n-6 + C20:4 n-6 + C18:3 n-3 + C20:5 n-3 + C22:5 n-3 + C22–6 n-3]/ [C14:0 + C16:0] (Flakemore, Malau-Aduli, Nichols, & Malau-Aduli, 2017).

References

Flakemore, A. R., Malau-Aduli, B. S., Nichols, P. D., & Malau-Aduli, A. E. O. (2017). Omega-3 fatty acids, nutrient retention values, and sensory meat eating quality in cooked and raw Australia lamb. Meat Science, 123, 79–87. https://doi.org/10.1016/j.meatsci.2016.09.006

Ulbricht, T. L. V., & Southgate, D. A. T. (1991). Coronary heart disease: seven dietary factors. Lancet, 338(8773), 985–992. https://doi.org/10.1016/0140-6736(91)91846-M

Supplementary Tables

Table S1. Basic information on 80 donkeys

NO.	Srain	Sex	Age (years)	Carcass weights (kg)	IMF content（%）	Group
DK01	Dezhou donkey	male	3	146.00	5.95	H-IMF 1
DK02	Dezhou donkey	male	2.5	121.40	5.50	H-IMF 2
DK03	Dezhou donkey	male	2	105.00	5.09	H-IMF 3
DK04	Dezhou donkey	male	2.5	135.80	4.66	H-IMF 4
DK05	Dezhou donkey	male	3	146.50	4.12	H-IMF 5
DK06	Dezhou donkey	male	2.5	121.60	4.12	H-IMF 6
DK07	Dezhou donkey	male	2.5	97.20	4.12	H-IMF 7
DK08	Dezhou donkey	male	2.5	121.20	4.11	H-IMF 8
DK09	Dezhou donkey	male	2	122.50	3.72	
DK10	Dezhou donkey	male	3	149.50	3.70	
DK11	Dezhou donkey	male	2.5	150.60	3.65	
DK12	Dezhou donkey	male	3	133.75	3.65	
DK13	Dezhou donkey	male	2.5	157.60	3.63	
DK14	Dezhou donkey	male	2.5	139.60	3.53	
DK15	Dezhou donkey	male	2	157.50	3.48	
DK16	Dezhou donkey	male	2.5	100.40	3.47	
DK17	Dezhou donkey	male	2.5	140.60	3.33	
DK18	Dezhou donkey	male	2.5	137.80	3.32	
DK19	Dezhou donkey	male	2.5	165.60	3.32	
DK20	Dezhou donkey	male	2.5	127.60	3.25	
DK21	Dezhou donkey	male	2.5	188.30	3.22	
DK22	Dezhou donkey	male	2.5	142.00	3.18	
DK23	Dezhou donkey	male	3	147.50	3.16	
DK24	Dezhou donkey	male	2.5	132.00	3.15	
DK25	Dezhou donkey	male	2.5	154.00	3.11	
DK26	Dezhou donkey	male	2.5	114.80	3.10	
DK27	Dezhou donkey	male	2.5	172.20	3.04	
DK28	Dezhou donkey	male	2.5	163.60	3.02	
DK29	Dezhou donkey	male	2.5	155.40	3.02	
DK30	Dezhou donkey	male	2.5	124.40	2.98	
DK31	Dezhou donkey	male	2.5	132.50	2.98	
DK32	Dezhou donkey	male	2.5	116.00	2.97	
DK33	Dezhou donkey	male	2.5	131.60	2.96	
DK34	Dezhou donkey	male	2.5	133.80	2.94	
DK35	Dezhou donkey	male	2.5	117.60	2.92	
DK36	Dezhou donkey	male	2.5	151.80	2.91	
DK37	Dezhou donkey	male	2	120.00	2.81	
DK38	Dezhou donkey	male	2.5	157.80	2.73	
DK39	Dezhou donkey	male	2.5	138.50	2.72	
DK40	Dezhou donkey	male	2.5	130.40	2.69	
DK41	Dezhou donkey	male	2.5	154.85	2.67	
DK42	Dezhou donkey	male	2.5	103.60	2.63	

DK43	Dezhou donkey	male	2.5	130.60	2.54	
DK44	Dezhou donkey	male	2	119.50	2.52	
DK45	Dezhou donkey	male	2.5	139.80	2.51	
DK46	Dezhou donkey	male	2.5	152.00	2.51	
DK47	Dezhou donkey	male	2.5	168.40	2.51	
DK48	Dezhou donkey	male	2.5	148.00	2.50	
DK49	Dezhou donkey	male	2.5	152.20	2.49	
DK50	Dezhou donkey	male	2.5	153.00	2.49	
DK51	Dezhou donkey	male	2.5	148.20	2.48	
DK52	Dezhou donkey	male	2.5	174.40	2.47	
DK53	Dezhou donkey	male	2.5	145.80	2.42	
DK54	Dezhou donkey	male	2.5	159.80	2.39	
DK55	Dezhou donkey	male	2.5	137.00	2.37	
DK56	Dezhou donkey	male	2	124.20	2.37	
DK57	Dezhou donkey	male	2.5	128.20	2.36	
DK58	Dezhou donkey	male	2	99.25	2.35	
DK59	Dezhou donkey	male	2.5	135.60	2.34	
DK60	Dezhou donkey	male	3	151.00	2.33	
DK61	Dezhou donkey	male	3	132.00	2.32	
DK62	Dezhou donkey	male	2.5	116.40	2.28	
DK63	Dezhou donkey	male	3	141.00	2.26	
DK64	Dezhou donkey	male	2.5	129.80	2.23	
DK65	Dezhou donkey	male	2	137.50	2.21	
DK66	Dezhou donkey	male	2.5	147.40	2.15	
DK67	Dezhou donkey	male	2.5	134.40	2.15	
DK68	Dezhou donkey	male	2.5	167.75	2.11	
DK69	Dezhou donkey	male	2.5	116.40	2.11	
DK70	Dezhou donkey	male	2.5	139.20	2.10	
DK71	Dezhou donkey	male	2.5	150.40	2.10	
DK72	Dezhou donkey	male	2.5	121.20	2.07	
DK73	Dezhou donkey	male	2	102.50	2.05	L-IMF 8
DK74	Dezhou donkey	male	2	117.50	2.05	L-IMF 7
DK75	Dezhou donkey	male	2.5	134.20	1.92	L-IMF 6
DK76	Dezhou donkey	male	2.5	128.00	1.87	L-IMF 5
DK77	Dezhou donkey	male	2.5	134.80	1.76	L-IMF 4
DK78	Dezhou donkey	male	2.5	139.20	1.76	L-IMF 3
DK79	Dezhou donkey	male	2.5	120.60	1.39	L-IMF 2
DK80	Dezhou donkey	male	2.5	117.60	1.34	L-IMF 1

H-IMF, high intramuscular fat group; L-IMF, low intramuscular fat group.

Table S2. Lipid molecular species identified in the low and high intramuscular fat groups using untargeted lipidomics (n=8).

Category	Class	Compounds	Average Mz	Adduct	Rt(min)	H-1	H-2	H-3	H-4	H-5	H-6	H-7	H-8	L-1	L-2	L-3	L-4	L-5	L-6	L-7	L-8
Sterol lipids	AHexBRS	ASG 28:2;O;Hex;FA 15:4	821.55988	[M-H]-	6.92	11197	13743	8928	7761	6950	12824	14291	5030	11080	12459	14220	15056	10233	6487	12810	15403
	AHexBRS	ASG 28:2;O;Hex;FA 16:2	839.60071	[M-H]-	7.551	3782	3357	12062	2151	2607	2707	2469	15230	4890	2141	2794	7408	3665	2278	1916	2313
	AHexCAS	ASG 28:1;O;Hex;FA 21:0	915.72363	[M-H]-	9.428	2068	3072	1925	2683	1867	412	1015	1621	486	456	870	156	1709	441	98	541
	AHexCS	ASG 27:1;O;Hex;FA 12:0	748.61426	[M+NH4]+	6.527	36527	4154	14227	29215	111617	62815	8701	60689	84259	6443	15941	29592	48013	27383	8314	33896
	AHexCS	ASG 27:1;O;Hex;FA 9:0	706.5705	[M+NH4]+	5.135	6309	10754	1483	9579	11410	16888	9175	14033	16463	5168	13437	14780	9476	12685	17988	10437
	BRSE	SE 28:2/14:0	626.58582	[M+NH4]+	6.523	10425	3631	13837	6505	10390	16276	9483	25339	24761	12139	11632	14865	14453	1189	990	7970
	BRSE	SE 28:2/17:1	666.61401	[M+NH4]+	9.216	186807	98093	114883	101778	132855	216621	116961	126656	147231	164477	147884	167459	173406	137521	243144	146205
	CASE	SE 28:1/14:0	628.60284	[M+NH4]+	8.122	1227	1636	1204	897	2198	3364	992	599	902	962	1220	485	2061	966	2240	1872
	DCAE	SE 24:1;O4/8:0	536.42719	[M+NH4]+	2.763	14397	17718	13985	14146	17885	15149	10376	16170	16885	13331	14066	18500	14711	17703	17561	13949
	DCAE	SE 24:1;O4/15:0	615.48877	[M-H]-	6.803	3124	2740	2339	2886	3820	2709	2203	2632	2277	2419	2697	2677	2180	2420	2395	2656
	DCAE	SE 24:1;O4/15:1;O1	629.49121	[M-H]-	7.909	1421	2015	1815	2002	2656	1434	2096	1117	1402	981	1262	1396	1842	1333	1158	902
	DCAE	SE 24:1;O4/19:2	667.53064	[M-H]-	3.545	4869	4811	4437	4281	4532	3864	3179	4962	4933	3308	4445	4298	4118	4455	4588	4552
	DCAE	SE 24:1;O4/20:0	685.58881	[M-H]-	7.581	12859	12738	12147	12911	11954	13019	14178	12695	14988	12791	15075	15087	13528	14425	13941	13480
	DCAE	SE 24:1;O4/22:0	713.62042	[M-H]-	7.95	47604	52219	51742	51318	46571	49023	47799	48771	53942	47383	53384	49848	47138	52065	46399	49879
	DCAE	SE 24:1;O4/22:0;O1	729.61896	[M-H]-	7.117	95272	98386	48514	97273	94512	101427	91749	87663	101955	96684	101791	95676	97199	98903	96627	96674
	DCAE	SE 24:1;O4/24:0;O1	757.63995	[M-H]-	6.911	12782	14841	14074	12116	14042	14503	14775	17651	15958	17663	13337	12193	7629	19100	11584	8683
	DCAE	SE 24:1;O4/25:0	755.65356	[M-H]-	8.437	3074	3831	3062	3221	3786	3230	3789	2989	3153	3860	3445	3354	3543	4005	3506	3337
	DCAE	SE 24:1;O4/25:0;O1	771.64862	[M-H]-	8.239	8988	8824	7926	8018	8274	9172	7681	7984	10405	6944	9860	8442	9542	8991	7867	8230
	DCAE	SE 24:1;O4/26:0;O1	785.66162	[M-H]-	8.367	2756	2832	2533	2449	2643	2648	2129	2677	2997	2561	2767	2727	2844	2888	1557	2398
	DCAE	SE 24:1;O4/27:0;O1	799.67841	[M-H]-	8.526	2975	3313	4567	3236	3308	3710	2972	4669	4615	3682	3335	2978	3605	3462	3130	3089
	SSulfate	ST 27:1;O;S	465.30423	[M-H]-	4.023	5242	4847	4947	4568	5610	4808	4543	4499	5317	4143	4387	4452	4489	4951	4151	4629
	SSulfate	ST 29:2;O;S	491.32001	[M-H]-	4.199	2537	3113	2520	1971	3746	2424	2499	2027	1663	671	1513	1227	3015	1467	648	738
	ST	ST 27:1;O	369.3483	[M+H-H2O]+	4.465	700449	605860	573924	662383	639509	643398	583428	604642	515975	545855	593840	514866	521527	600612	552853	509951

	ST	ST 27:2;O	385.34421	[M+H]+	4.274	32054	42390	28382	52448	43642	15841	28540	27549	13558	12210	21853	13989	27109	14271	12365	12311
	ST	ST 28:1;O	383.35965	[M+H-H2O]+	9.341	1910	3846	899	2376	2540	2251	1593	2022	2197	3888	2631	1329	1308	1710	2178	0
	ST	ST 29:1;O	397.37933	[M+H-H2O]+	9.456	52373	46324	42563	32132	39584	41861	35583	39087	41490	36219	53203	41117	52401	38591	50490	36673
	ST	ST 29:2;O	395.36215	[M+H-H2O]+	8.252	10958	16515	15228	7173	15101	11620	14722	14284	16492	13461	15976	15230	595	742	13127	11832
**Glycerol phospholidis**	BMP	BMP 32:0|BMP 16:0_16:0	740.53302	[M+NH4]+	4.267	56326	6108	144907	11933	39223	51216	8353	31037	66190	60349	32300	54868	90135	53865	56812	57580
	CL	CL 70:3|CL 16:0_16:0_16:0_22:3	714.50824	[M-2H]2-	6.795	35541	45312	76018	43475	61322	48520	38374	48895	67160	47595	42940	61646	95316	58723	60151	73387
	CL	CL 72:7|CL 18:1_18:2_18:2_18:2	724.4881	[M-2H]2-	8.735	3882	2088	2929	2634	3413	3521	3804	2529	4184	2409	2778	4018	3011	2982	2854	3188
	CL	CL 72:8|CL 18:2_18:2_18:2_18:2	723.47913	[M-2H]2-	8.564	20964	15215	14108	13125	17789	14874	15879	14104	21009	15872	17882	23704	20905	18162	17104	19723
	CL	CL 75:6|CL 17:0_18:0_18:0_22:6	746.51044	[M-2H]2-	6.874	2634	5083	4586	4927	3698	4952	4179	4835	8809	2614	4354	6252	10216	5743	5852	7145
	CL	CL 78:1|CL 16:0_16:0_28:0_18:1	772.58398	[M-2H]2-	7.991	5756	3434	3154	3177	3687	6139	3486	4219	2813	2950	5637	4482	4831	5218	3369	4323
	CL	CL 82:16|CL 20:4_20:4_20:4_22:4	785.50977	[M-2H]2-	7.441	3485	4259	2283	2777	358	3135	2156	3298	2748	2181	2378	3264	2471	2363	3135	1415
	CL	CL 83:18|CL 17:0_22:6_22:6_22:6	790.50238	[M-2H]2-	6.977	5028	6789	7421	6397	6561	6566	5218	6540	5973	5679	3808	5867	3938	5455	5302	5228
	CL	CL 12:0_12:0_12:0_24:0	647.45435	[M-H]-	4.193	3021	3267	2560	2271	4107	1778	2238	1845	1963	1152	1435	1536	2678	1523	1193	919
	CL	CL 12:0_12:0_14:0_17:0	612.41687	[M-H]-	4.025	2725	2735	2950	2349	3180	2227	2227	2523	2640	1858	2300	2341	3052	2853	2335	2092
	CL	CL 12:0_15:0_18:0_28:0	738.5509	[M-H]-	7.332	2421	2002	451	622	3370	3579	1482	1537	2665	1273	3445	1793	4986	2850	3830	2363
	CL	CL 12:0_15:1_26:0_22:6	745.5069	[M-H]-	5.978	2610	1406	751	1305	1589	1964	2192	1548	1852	1386	1319	2776	2280	1500	1677	2299
	CL	CL 12:0_28:0_28:0_28:0	899.73456	[M-H]-	9.406	2455	3682	1812	3509	2131	923	1668	1988	449	610	1048	382	2067	848	463	991
	CL	CL 16:0_28:0_28:0_28:0	927.7674	[M-H]-	9.58	3087	4025	2289	3573	2829	859	1801	2125	637	718	732	394	2066	775	327	505
	CL	CL 28:0_16:2_28:0_28:0	925.75024	[M-H]-	9.428	2021	3312	1998	2616	1571	474	1033	1258	413	502	730	287	1876	483	223	531
	EtherLPC	LPC O-16:1	524.33282	[M-H]-	2.626	2259	133	244	158	159	323	174	499	355	269	351	2844	1181	1030	48	162

EtherLPC	LPC O-16:2	522.3205	[M-H]-	5.904	4505	3744	3832	4086	3722	2635	3107	5162	4785	2937	2846	4185	3851	4438	4396	4591
EtherLPE	LPE O-16:1	436.28152	[M-H]-	2.753	6088	750	642	805	1879	3543	848	2154	1884	316	425	9468	5648	889	789	916
EtherLPE	LPE O-18:1	464.31262	[M-H]-	3.741	17841	6354	5227	6889	4078	7883	6423	6762	5502	1970	2750	20212	10626	6500	2324	4156
EtherLPE	LPE O-18:2	462.29736	[M-H]-	2.903	16387	4459	3242	3661	5399	8090	4078	7732	3594	314	2675	19222	14147	3392	2620	2975
EtherLPE	LPE O-18:4	460.27615	[M+H]+	4.77	50421	54332	5248	21934	38325	50824	46268	37943	69832	13232	46707	62833	73687	42923	35553	29687
LPE	LPE 18:0	480.30887	[M-H]-	3.313	25196	3555	5143	7334	4965	16529	7110	7869	5242	2596	2399	23972	20403	5647	2262	3398
EtherLPG	LPG O-18:1	495.30054	[M-H]-	5.903	3014	2423	2642	2557	3138	2290	2505	3144	2960	2345	2665	2709	2264	2634	2849	2506
EtherLPG	LPG O-24:6	569.33289	[M-H]-	9.707	2728	3201	1742	2654	2835	1134	1880	2063	199	271	753	326	1214	854	816	747
LPI	LPI 6:0	431.13831	[M-H]-	5.023	5930	5849	5149	5033	5442	5011	4045	5128	6024	4463	4790	4893	4790	5838	5960	5104
EtherOxPC	PC O-14:0_22:3;O2	846.5871	[M-H]-	6.897	3462	4822	4047	5447	3673	3998	4385	2303	6140	3935	986	4034	3631	2552	3551	1903
EtherOxPC	PC O-15:0_22:5;O2	856.57135	[M-H]-	7.369	9802	9466	8990	10960	12314	9705	9355	8652	11716	9132	11427	10775	9313	7800	8975	8779
EtherOxPC	PC O-15:0_22:5;O3	872.56171	[M-H]-	7.369	9554	9411	11287	7220	8982	9002	8536	9191	7287	3564	9387	9043	9131	9692	8903	6313
EtherOxPC	PC O-15:0_22:6;O2	854.55389	[M-H]-	7.225	10467	14019	10931	13218	7700	5388	12365	10809	13370	10503	11364	11449	9598	9922	7563	6120
EtherOxPC	PC O-17:0_22:6;O2	882.58496	[M-H]-	7.793	5854	9978	1289	8329	4037	1655	934	671	6079	1352	10686	12005	3467	6115	1885	3083
EtherOxPC	PC O-17:1_22:6;O2	880.56519	[M-H]-	7.282	4497	4433	3759	6201	7599	1461	4287	7114	8173	7038	8572	3989	5947	8652	2004	2342
EtherOxPC	PC O-17:2_22:6;O3	894.54419	[M-H]-	6.521	5145	4721	2059	3929	3533	5795	4967	3791	6556	3724	4434	5006	5710	3907	4666	6088
EtherOxPC	PC O-22:3_22:6;O4	978.59375	[M-H]-	5.288	2280	1898	1816	1769	1884	1755	1655	1672	1945	1765	1726	1695	1584	1933	1933	1825
EtherOxPC	PC O-8:0_9:0;O2	586.32727	[M-H]-	5.03	4540	5132	2949	3819	4309	3960	4420	4027	4449	4277	3550	4399	4095	3203	3877	2046
EtherOxPC	PC O-9:0_22:4;O4	790.49402	[M-H]-	6.885	141	228	7421	3716	6223	79	276	65	729	566	637	350	192	329	4301	348
OxPC	PC 10:0_22:5;O4	848.44897	[M-H]-	7.044	1159	1195	1620	288	1323	1202	363	1656	1619	594	1611	1611	1414	1572	1243	1005
OxPC	PC 14:0_18:1;O4	840.52618	[M+FA-H]-	7.546	1677	2238	1775	2007	1983	1921	2615	1780	2021	1745	2135	2406	2464	1830	1841	2745
OxPC	PC 14:0_22:6;O4	886.51135	[M-H]-	6.909	167	347	6052	392	2469	129	1498	3784	975	1641	1751	1434	4997	3089	3809	3144
OxPC	PC 15:1_20:5;O3	856.50018	[M-H]-	7.549	6883	8070	6141	6073	5686	5690	7726	5288	7358	5559	6446	7468	6950	5783	6315	6518
OxPC	PC 15:1_20:5;O4	872.4942	[M+FA-H]-	7.28	6866	6942	7439	7792	6491	8049	8814	6264	8120	7105	6574	7983	8441	6923	7395	9433
OxPC	PC 15:1_22:5;O4	916.52075	[M-H]-	7.229	2718	3902	2714	4833	2871	1643	2833	2728	3076	2246	2783	2698	2590	3264	3133	2983
OxPC	PC 17:0_18:1;O4	882.57397	[M-H]-	7.392	315	3389	2576	574	8345	413	673	6213	1901	1897	8024	4520	7028	3287	6908	5818
EtherOxPE	PE O-12:0_17:1;O2	664.44983	[M-H]-	4.196	1876	2650	1063	1556	2614	1240	1392	1346	1210	773	923	951	1744	1110	846	639
EtherOxPE	PE O-16:0_22:3;O1	770.56927	[M-H]-	7.516	23970	16994	10676	15236	12595	27816	20420	22594	18272	12505	26028	16480	21533	26270	19187	12291
EtherOxPE	PE O-16:0_22:4;O2	784.54938	[M-H]-	6.839	3818	1659	3008	4635	5164	4466	4416	4233	8209	2965	8334	3719	3732	4408	4341	4579
EtherOxPE	PE O-16:0_28:0;O1	860.71173	[M-H]-	9.383	5827	3195	1162	4602	1696	1193	4317	2914	69	234	583	60	229	125	131	163

EtherOxPE	PE O-17:0_22:5;O2	796.5495	[M-H]-	7.866	2096	2906	1575	980	1577	479	2241	2191	2073	1650	2209	1416	815	1988	1396	1129
EtherOxPE	PE O-17:2_22:6;O3	806.49329	[M-H]-	6.437	2082	1886	2305	1627	2209	2431	1815	1564	3431	2098	1720	2630	2859	1878	2283	2951
EtherOxPE	PE O-18:0_18:1;O1	746.56677	[M-H]-	6.648	28713	45446	46371	28556	0	227	0	31988	31871	55	31724	49726	48288	48003	0	0
EtherOxPE	PE O-18:0_22:5;O2	810.56726	[M-H]-	6.836	63935	56732	46323	44250	46583	22552	49756	42183	58255	44019	65652	66640	44575	74712	13473	23500
EtherOxPE	PE O-18:0_28:0;O1	888.74341	[M-H]-	9.571	12091	8102	3167	9321	4522	3371	8585	5345	721	1514	2539	864	3381	1602	1533	1766
EtherOxPE	PE O-18:1_22:6;O1	790.5376	[M-H]-	6.894	1196	1216	1402	809	774	1894	1852	924	1354	1261	965	3221	4103	2347	991	2030
EtherOxPE	PE O-22:3_22:4;O2	862.59412	[M-H]-	7.162	6349	3571	3939	4311	5603	2277	3607	5336	6264	3463	5394	5859	4767	6867	2318	2646
EtherOxPE	PE O-24:0_20:5;O2	866.62671	[M-H]-	7.727	5708	3170	1636	1900	5792	1038	2944	953	3833	2984	3466	2426	2504	2649	2330	1689
EtherOxPE	PE O-26:0_17:0;O2	862.68536	[M-H]-	9.283	4107	3863	3503	3494	3428	3507	3954	3621	3677	2477	3949	3816	3739	3293	2941	3001
EtherOxPE	PE O-28:0_9:0;O2	778.59204	[M-H]-	6.814	5645	3815	4077	4127	4321	4359	3412	3285	3538	4454	4999	4361	4064	3455	5325	3166
EtherOxPE	PE O-8:0_28:0;O1	748.58484	[M-H]-	6.604	1532	11018	12357	4902	0	11732	1858	25916	10172	3451	7655	15884	21350	20840	0	13383
EtherOxPE	PE O-9:0_28:0;O1	762.60052	[M-H]-	6.81	293856	294466	327696	302035	326160	313954	278544	316676	393595	286660	326058	297659	314755	309710	313132	304532
OxPE	PE 37:5;O|PE 22:4_15:1;O	766.50073	[M-H]-	7.078	7039	8030	7822	8208	7115	7364	7043	7515	8976	4967	5829	6561	8895	5281	10324	8093
OxPE	PE 39:6;O|PE 22:5_17:1;O	792.51758	[M-H]-	7.133	11326	14327	11137	12083	11260	10692	14675	9615	12019	8931	11512	11813	11658	7078	12486	13633
OxPE	PE 15:1_22:5;O4	828.47241	[M-H]-	7.083	1175	2614	2022	2897	2755	3268	2884	2569	2450	1098	2169	2633	3108	872	2997	2879
OxPE	PE 18:3_22:5;O4	866.46924	[M-H]-	7.194	2702	973	1557	647	890	2192	1573	680	1874	1595	1615	1943	1476	1604	1121	707
OxPG	PG 16:0_18:2;O1	761.50366	[M-H]-	7.084	2994	1460	2851	3049	2973	3071	3484	2856	3795	1723	2260	3104	3882	1532	4161	3572
OxPG	PG 17:0_18:1;O3	809.51715	[M-H]-	8.185	11353	10884	10738	11230	11518	10683	10621	11227	11455	10412	11792	11485	11231	11453	10980	10437
OxPG	PG 17:0_22:5;O3	857.51947	[M-H]-	7.289	1035	1008	1406	2043	1401	1654	1483	1597	1610	1211	1110	1548	1369	2025	1720	2061
OxPG	PG 18:2_22:3;O4	887.53394	[M-H]-	6.904	7030	3482	7518	4039	4042	3553	8226	3887	7933	8147	6266	5855	7375	6028	8228	3051
OxPG	PG 18:2_22:6;O1	833.50226	[M-H]-	7.038	10732	12420	13024	10987	12531	11822	11792	14519	12973	12994	13434	12448	12197	15966	10364	11933
OxPG	PG 18:3_22:6;O1	831.49066	[M-H]-	6.65	3786	4773	4718	3853	3210	3277	3148	3866	3951	3606	4662	4037	5254	4574	2880	3412
OxPG	PG 22:3_22:5;O4	953.54211	[M-H]-	6.296	3675	1514	336	936	1773	1876	2169	1576	1291	1463	1301	2470	926	1413	460	274
OxPG	PG 26:0_20:5;O4	971.6217	[M-H]-	6.829	3027	2298	306	1442	2255	2993	3061	1536	1694	2389	639	2544	112	131	1911	1708
OxPG	PG 8:0_3:0;O1	443.16757	[M-H]-	3.439	3902	3693	3462	3188	3034	2593	2016	2502	3198	1632	2118	2956	4616	2311	2023	1871
OxPI	PI 15:0_16:0;O3	843.4798	[M-H]-	7.287	2520	3094	3552	3348	3315	1901	3267	2959	4180	2287	1538	2941	3205	3735	1580	4164
OxPI	PI 16:0_22:3;O1	903.56439	[M-H]-	5.03	2052	1770	1755	1689	1604	1772	1420	1736	2056	1338	1710	1602	1795	1956	1496	1230
OxPS	PS 16:1_22:6;O3	852.46838	[M-H]-	6.989	3005	2630	2958	2776	2377	3176	2646	3311	3391	2668	2634	2821	2785	2259	2418	2864
OxPS	PS 18:1_22:6;O3	880.50128	[M+FA-H]-	7.456	4353	5542	5452	4289	4461	4527	4344	4652	5411	5255	5231	4934	4916	4521	3546	3358

OxPS	PS 18:1_22:6;O4	896.49866	[M-H]-	7.198	5167	2498	4405	2537	2498	5421	3039	2047	3266	4306	3364	3123	3615	3072	3064	3159
OxPS	PS 18:2_22:6;O3	878.48456	[M-H]-	7.037	3968	4347	4772	3935	3905	3991	4608	4705	3985	3892	4147	4183	4074	5262	3793	3841
OxPS	PS 22:3_22:6;O2	916.53613	[M-H]-	6.906	4761	5798	4177	5736	2469	3480	6232	5046	6449	4974	5541	6332	3083	6157	5988	6229
OxPS	PS 22:6_7:0;O1	696.34625	[M-H]-	7.076	1669	1765	1885	1783	1753	2185	1602	1800	2454	1220	1710	2243	2629	1525	2866	2402
PA	PA 10:0_28:2	727.52814	[M-H]-	6.877	621	188	243	97	323	10625	117	607	158	220	330	685	1363	169	172	7503
PA	PA 15:0_28:7	787.52777	[M-H]-	7.118	1418	3200	3064	2639	2979	1463	2727	1899	2072	2254	2965	851	2474	754	3123	3238
PA	PA 15:2_28:7	783.49127	[M-H]-	7.082	11293	12165	11215	13029	11574	13206	11570	11755	12938	9378	10449	12456	12979	8046	14238	13696
PA	PA 17:0_28:3	823.62488	[M-H]-	6.449	3695	18906	4370	8714	10651	11534	2672	17772	3673	5228	18144	16146	17417	2803	9021	8862
PA	PA 19:0_28:7	843.5932	[M+FA-H]-	6.441	24403	21078	32771	68373	39391	38769	40444	70529	48096	69644	34891	37658	40902	32425	24661	44366
PA	PA 19:4_28:7	835.52063	[M-H]-	7.454	13622	16490	19126	16967	17337	14531	14916	18221	18918	19523	17523	16922	15490	18471	14639	16416
PA	PA 22:0_28:5	889.6698	[M-H]-	8.113	231	1494	411	1587	797	119	428	3454	624	817	1493	577	328	1604	663	1834
PA	PA 22:0_28:6	887.65552	[M-H]-	7.75	336	1593	158	1335	737	413	458	2835	586	645	1125	319	70	1296	674	1757
PA	PA 8:0_26:6	663.40997	[M-H]-	4.021	2046	2400	2271	1111	1514	2044	2263	2077	2476	1592	2023	2034	2380	2148	1825	2010
PA	PA 8:0_28:1	701.51263	[M-H]-	6.75	74	30	30	55	81	4504	79	51	37	66	78	120	694	91	64	2335
EtherPC	PC O-30:0	692.55139	[M+H]+	4.798	17189	37050	25962	14869	60367	65774	23868	34026	35082	25691	53411	32929	36883	33613	23354	58985
EtherPC	PC O-32:0|PC O-16:0_16:0	720.58582	[M+H]+	5.58	279230	304477	309890	237230	437942	535526	110106	390538	447716	221937	375625	302013	399995	319284	257425	381605
EtherPC	PC O-32:1|PC O-16:1_16:0	718.56757	[M+H]+	5.366	173629	236408	48037	134832	226936	149536	164464	272583	286996	130661	283067	274578	286077	316189	193902	123476
EtherPC	PC O-32:3	714.53955	[M+H]+	4.135	49330	72527	57188	34985	68134	49205	35054	81131	104429	42129	102313	108151	148691	157047	61262	59814
EtherPC	PC O-33:2|PC O-15:0_18:2	730.56854	[M+H]+	4.645	405732	350788	235441	299375	617053	541553	364836	405414	616442	328302	588342	625288	616904	495466	468731	524095
EtherPC	PC O-33:3|PC O-15:1_18:2	728.55194	[M+H]+	4.453	137019	239579	133560	76507	215546	105438	132993	202239	179972	132677	182888	307618	148715	427374	99417	130104
EtherPC	PC O-34:1	746.60352	[M+H]+	6.311	30753	20936	2731404	19111	28337	29157	18445	20939	8161	19788	29290	7623	13350	4052	45458	15193
EtherPC	PC O-34:1|PC O-16:0_18:1	790.59967	[M+HCOO]-	7.883	182244	74900	65895	39858	112699	95122	69884	55144	85589	57865	82447	68577	36299	27447	61305	50058
EtherPC	PC O-34:1|PC O-18:1_16:0	746.60559	[M+H]+	5.591	7906794	3041470	11092	1272947	2583322	4667156	2289582	2800041	3940346	2095263	3005330	2942184	2904788	2229696	3114148	1216837
EtherPC	PC O-34:2|PC O-16:0_18:2	744.57947	[M+H]+	4.987	42674412	40555452	25121172	32562136	59834096	50546968	27073568	38170108	47618096	29972718	58644960	50439392	46505788	36471668	40696604	37741004
EtherPC	PC O-34:2|PC O-16:1_18:1	744.59015	[M+H]+	5.434	15624641	6585264	5606965	1896486	4820188	3374900	3982953	4091352	3846188	2092077	7195657	1946700	4581506	5901026	2870262	660070
EtherPC	PC O-34:3|PC O-16:1_18:2	742.5741	[M+H]+	4.815	53092772	57937988	248655	49331876	53826988	42860284	47977444	63359252	84907800	45051616	72173424	77312072	72161216	71427984	37883052	46400796
EtherPC	PC O-34:4|PC O-18:4_16:0	740.5528	[M+H]+	4.348	101115	131132	3653	137128	201502	192609	47966	170572	274242	133087	283461	259808	172230	369999	184601	216876
EtherPC	PC O-34:6	736.51068	[M+H]+	5.337	37033	31397	9956	33663	33982	35826	34639	34507	42614	33289	29930	41660	4506	3921	39305	3355
EtherPC	PC O-35:2|PC O-17:0_18:2	758.59521	[M+H]+	5.338	290208	373626	539812	186489	633687	362088	140562	354299	588180	375252	478564	555871	833080	304272	241412	440791
EtherPC	PC O-35:3|PC O-17:1_18:2	756.58673	[M+H]+	5.173	1719138	1695778	203518	927995	1449754	1557340	827447	830528	1713235	543470	2354288	1124988	1796221	2885386	855733	542842

EtherPC	PC O-35:4|PC O-17:2_18:2	754.56647	[M+H]+	4.52	115220	55726	150662	102867	57117	16994	142140	17233	128528	20908	93065	163768	198278	143639	79466	149710
EtherPC	PC O-35:5|PC O-15:1_20:4	752.55548	[M+H]+	4.726	288124	397045	321604	493513	571633	755807	197579	432232	513792	338930	618756	385552	573665	85957	753114	611515
EtherPC	PC O-35:6|PC O-15:4_20:2	794.5329	[M+HCOO]-	7.549	13108	20698	12002	14568	13997	11947	15978	13555	15294	11372	13825	18217	15351	15017	13892	13058
EtherPC	PC O-35:7	748.5199	[M+H]+	4.768	109340	118386	57981	111869	111210	120978	129895	73551	97498	98222	108044	109710	113774	63610	142580	128852
EtherPC	PC O-35:8	746.50409	[M+H]+	4.514	27808	14218	1001	51856	3618	53657	50842	64171	54437	58568	54272	64527	49115	47504	44381	1660
EtherPC	PC O-36:1	774.62915	[M+H]+	6.436	239290	61431	17249	43873	170065	120012	20374	107058	44093	76792	143765	98212	71660	119972	113405	27699
EtherPC	PC O-36:2	772.62085	[M+H]+	6.883	1383707	26860	14563	31095	51673	33625	34508	32321	31701	16163	49911	48884	37128	34808	41939	16425
EtherPC	PC O-36:2|PC O-17:1_19:1	772.60974	[M+H]+	2.319	15166	11819	13203	13982	16689	11387	13351	29475	19369	10975	13806	13612	12602	20526	21265	24824
EtherPC	PC O-36:2|PC O-18:0_18:2	772.61957	[M+H]+	5.825	5324654	2762933	2542747	2396481	6819338	4018266	2282546	3123103	4059076	1997644	4714104	3323778	4093675	3257757	2024099	2437380
EtherPC	PC O-36:3|PC O-18:1_18:2	770.60376	[M+H]+	5.05	20119942	18208008	6444651	12963749	28327828	14852994	12422897	16900212	20987742	12615865	23009252	23242166	19961368	17499914	13279801	13752126
EtherPC	PC O-36:4	768.58289	[M+H]+	5.527	360687	60526	15857993	85928	298070	206614	38404	55534	73543	63061	138824	151895	51792	68813	55020	53653
EtherPC	PC O-36:4|PC O-18:2_18:2	812.58344	[M+HCOO]-	7.255	370606	364014	292606	254413	308383	186540	318001	351089	372408	284656	451984	410469	135458	449170	135350	185983
EtherPC	PC O-36:4|PC O-18:4_18:0	768.58276	[M+H]+	4.87	26596928	23200890	2154119	14134115	27150694	13346036	18076886	24940384	28836774	20770826	34293400	31792262	23953074	35431056	10202273	14515627
EtherPC	PC O-36:5|PC O-16:1_20:4	766.57397	[M+H]+	4.657	7364462	5596150	2931354	4815373	6413192	3684388	3304715	9090747	7929306	5541253	10559254	8588333	5036850	12530551	2450884	3092264
EtherPC	PC O-36:5|PC O-18:3_18:2	810.56531	[M+HCOO]-	6.515	6890	7436	8466	11270	10079	12391	6489	6579	10148	5089	10159	11471	13509	9655	10057	11027
EtherPC	PC O-36:5|PC O-18:4_18:1	766.57257	[M+H]+	4.351	3913770	4046650	7429058	2764126	3640637	1498484	2890946	3127207	4079808	3196410	5052202	5004446	3947564	5684486	803436	1628828
EtherPC	PC O-36:6|PC O-18:4_18:2	764.55017	[M+H]+	4.811	514968	590284	8661	627215	545340	512829	597231	599306	617606	585323	623197	623587	623219	335127	531457	591587
EtherPC	PC O-36:8	760.5213	[M+H]+	5.303	570	1344	507	527	1144	1908	965	1047	510	1247	1017	509	764	498	557	0
EtherPC	PC O-36:9|PC O-18:4_18:5	758.50751	[M+H]+	4.49	1513	757	0	1452	5239	1010	1008	2805	505	11037	4561	7615	6700	757	1261	6182
EtherPC	PC O-37:1	788.66217	[M+H]+	6.495	8301	14322	4546	5692	3643	20166	2612	31118	14960	8720	20654	10283	9884	26296	12319	5662
EtherPC	PC O-37:10	770.51495	[M+H]+	4.711	1014	2348	3189	1145	1678	2045	2352	1166	3707	3933	2506	2938	1264	979	4915	1288
EtherPC	PC O-37:2	786.64752	[M+H]+	6.911	17042	66167	38914	3521	38664	2172	2586	58108	4684	2514	3137	4321	2183	3362	6846	2513
EtherPC	PC O-37:5	780.58081	[M+H]+	4.906	68719	13853	0	136563	108472	44863	17929	77178	72902	83548	77231	104561	132966	166025	7606	22480
EtherPC	PC O-37:5|PC O-18:1_19:4	780.58478	[M+H]+	4.987	75959	98879	0	124852	72780	108840	29200	163152	50623	186175	217998	139601	106541	145629	41649	75541
EtherPC	PC O-37:6|PC O-18:4_19:2	778.5675	[M+H]+	4.428	19127	29775	4362	7207	5062	2015	2748	27645	40243	16888	6289	31228	40424	43060	105491	5490
EtherPC	PC O-37:7|PC O-18:3_19:4	776.55341	[M+H]+	5.056	10014	81328	260701	86940	80810	90649	57972	93202	113549	68883	120084	110553	134248	127143	97359	85961
EtherPC	PC O-37:8	774.53424	[M+H]+	5.219	137169	143650	109736	127467	125007	117575	103379	146605	118408	130034	127820	128188	140425	159106	106095	95028
EtherPC	PC O-37:8|PC O-15:2_22:6	774.53857	[M+H]+	4.467	62734	86039	28742	96433	83575	54279	80040	99058	124971	90897	119843	111130	114928	125275	124756	106211

EtherPC	PC O-37:9	772.52905	[M+H]+	4.764	2626	65713	4006	19964	9742	61172	32265	75090	31557	4179	40344	47902	63650	67638	5581	21974
EtherPC	PC O-37:9|PC O-18:4_19:5	772.52203	[M+H]+	4.664	80326	61046	1300	66409	9742	12977	18535	66335	82844	60762	49754	46315	37048	69729	49289	33630
EtherPC	PC O-38:3|PC O-18:2_20:1	798.63007	[M+H]+	5.801	160763	86336	11259	44732	150754	85358	19018	106446	79845	94053	110404	182210	173160	144746	60926	73287
EtherPC	PC O-38:4|PC O-18:1_20:3	796.61481	[M+H]+	5.213	123912	194328	46325	217371	421046	219509	124547	260204	158328	214962	308198	106759	220176	278315	268271	154447
EtherPC	PC O-38:4|PC O-18:2_20:2	796.61182	[M+H]+	5.336	417299	270813	364422	114800	509127	236427	182223	264139	251028	194668	428882	476234	295370	406708	218026	161276
EtherPC	PC O-38:5	794.60022	[M+H]+	5.375	934688	711247	1918142	452662	850665	152899	374782	354626	759314	688154	1212906	883608	591309	1640698	131000	322423
EtherPC	PC O-38:5|PC O-18:1_20:4	794.59937	[M+H]+	5.063	651134	254957	16779	222905	301004	571597	216130	565304	156051	606393	876821	665440	150691	817146	148381	1502308
EtherPC	PC O-38:5|PC O-18:4_20:1	794.60272	[M+H]+	4.841	2492580	1724680	19508	1546319	3791660	1996708	984826	2402423	2305411	2069042	3050974	2591067	1772841	3315660	1381099	1342984
EtherPC	PC O-38:6|PC O-17:1_21:5	792.58398	[M+H]+	5.584	250848	165328	226012	95388	225872	119548	110665	200297	197813	152282	322928	314574	246710	336660	79641	134268
EtherPC	PC O-38:6|PC O-18:2_20:4	792.5882	[M+H]+	4.713	2822222	2130424	394975	1600776	2583244	1425532	1409185	3745495	3192671	2674581	4242052	3548706	2081478	5432756	930650	1220926
EtherPC	PC O-38:6|PC O-18:4_20:2	792.58551	[M+H]+	4.3	351750	351587	955	279094	476495	124062	144017	371050	322931	259269	547478	417575	394897	588723	165077	176599
EtherPC	PC O-38:7|PC O-18:3_20:4	790.57184	[M+H]+	4.178	390480	313115	370767	209285	297876	129862	154422	400212	330980	341660	630736	482880	282849	933038	36282	133794
EtherPC	PC O-38:7|PC O-18:4_20:3	790.56256	[M+H]+	4.882	313857	276190	125900	235409	303148	64316	265562	290077	283584	185362	237645	286354	191048	347150	155186	205284
EtherPC	PC O-38:8	788.5509	[M+H]+	4.665	133414	125290	194298	123069	101295	92110	92573	176218	90222	148105	193811	160785	109058	162404	32978	23548
EtherPC	PC O-38:8|PC O-18:3_20:5	788.55096	[M+H]+	4.391	67945	91168	121044	70582	103032	77919	69820	84200	71581	74661	99545	119394	98249	118467	32978	72070
EtherPC	PC O-39:10	798.53149	[M+H]+	5.027	31554	71250	31596	33137	22672	24290	26476	34575	20762	19915	34434	54065	31277	36096	40004	25855
EtherPC	PC O-39:2	814.6712	[M+H]+	6.966	17979	82474	5252	149516	213484	42195	186175	219066	37813	167220	61747	129807	79714	39975	17899	2643
EtherPC	PC O-39:3	812.65112	[M+H]+	7.489	28398	25656	24115	18880	27115	50237	20994	26794	46799	28845	30249	77977	38095	34386	53205	46690
EtherPC	PC O-40:11	810.53827	[M+H]+	4.862	1765	4975	9260	7279	9430	2763	3107	4056	4499	7037	4281	8180	7939	4536	2740	4975
EtherPC	PC O-40:2	828.68701	[M+H]+	7.302	222398	184049	19341	188098	201158	216326	211159	197045	264195	208750	197242	219748	195712	201362	195801	220744
EtherPC	PC O-40:5|PC O-18:1_22:4	822.63025	[M+H]+	5.662	133544	73120	108763	45554	134604	48152	12982	110141	128947	99628	15657	14350	53447	36159	12956	77852
EtherPC	PC O-40:6|PC O-18:2_22:4	820.61322	[M+H]+	4.89	236472	131153	1699	190704	297649	170596	130880	201862	213773	177846	217569	157165	210481	244493	188188	174857
EtherPC	PC O-40:7|PC O-18:2_22:5	818.60132	[M+H]+	4.756	237303	135931	124255	141615	234654	136820	104338	192096	266948	203460	243778	253617	189986	312688	98388	34477
EtherPC	PC O-40:8	816.58514	[M+H]+	4.436	20538	6495	5744	4527	7777	3526	25595	17295	55588	3964	10062	16852	5667	63407	2496	1475
EtherPC	PC O-40:9	814.5675	[M+H]+	4.725	64048	41376	69277	37258	58653	28307	38904	74830	62304	69599	91025	68304	43957	92481	27773	29194
EtherPC	PC O-41:11	824.55115	[M+H]+	5.189	94355	86417	82989	119495	141753	187981	82089	105993	163430	105710	165936	111155	144792	89739	201455	119659
EtherPC	PC O-11:0_28:7	848.58563	[M-H]-	7.506	13316	18109	6578	22388	6608	9766	7443	25854	6701	5933	25788	24074	13799	28613	7992	20740
EtherPC	PC O-15:2_22:6	818.52667	[M-H]-	7.455	11769	13259	14481	13955	13691	12228	13261	14025	13860	13945	16789	13498	13638	17060	9902	11029

EtherPC	PC O-15:3_22:6	816.5127	[M-H]-	7.038	12335	11465	12399	10535	10862	10184	10033	12249	12296	10730	12727	11802	10727	12032	9535	9988
EtherPC	PC O-15:4_22:6	814.50281	[M-H]-	6.649	3314	3453	3748	3014	2448	2492	2488	3140	3298	2609	2773	3215	3818	3764	1674	1685
EtherPC	PC O-21:0_28:1	1000.83032	[M-H]-	7.976	4225	3842	3721	3620	3911	3627	3820	3214	3482	3553	4161	3512	1727	4099	3542	3105
EtherPC	PC O-23:0_28:1	1028.85901	[M-H]-	8.071	16817	17074	17556	16977	17670	16733	16491	17062	18298	17440	19524	17864	17039	19255	19470	18327
EtherPC	PC O-9:0_26:2	802.59058	[M-H]-	7.709	15262	3294	0	3817	14247	17686	11812	0	8639	2196	6987	18436	0	0	0	0
EtherPC	PC O-9:0_28:5	824.57507	[M-H]-	7.32	3782	2069	2395	2798	5841	1406	943	2711	2737	4321	3232	4371	1546	9880	998	1389
EtherPC	PC O-9:0_28:7	820.54285	[M-H]-	7.447	423	1110	2011	1576	2805	982	884	1388	1300	2099	1947	1571	1859	2275	965	1799
PC	PC 30:0	706.53662	[M+H]+	4.239	184011	217592	250142	146799	231712	350021	138616	407011	238322	164815	278937	310687	672871	417634	364999	365588
PC	PC 31:0	720.54663	[M+H]+	4.599	53044	65238	82143	64185	58816	87470	55113	156433	75737	53299	145294	125989	188435	91014	74050	126472
PC	PC 32:0|PC 16:0_16:0	734.56659	[M+H]+	4.93	1374065	1997190	5485	1545989	1993873	2648950	1231646	3062322	1981062	1598718	2440986	2307035	4218965	3579708	1950626	2404687
PC	PC 32:1	776.5448	[M+HCOO]-	6.825	26033	28860	38469	20723	26201	33255	22698	31113	27905	17395	22186	26970	31962	27220	38395	29627
PC	PC 32:1|PC 16:0_16:1	732.552	[M+H]+	4.329	967739	1306545	2487727	809364	1256320	1754013	807565	1513121	1257362	681410	997702	1152124	1759710	1196789	2400843	1461213
PC	PC 32:2|PC 16:1_16:1	730.53766	[M+H]+	3.754	481427	902566	943970	669283	934606	1126732	631308	1235047	1232822	807954	1114281	1251834	2096741	1355904	1826045	1552367
PC	PC 32:3	772.52582	[M+HCOO]-	6.677	7571	6765	8670	8983	5637	8410	6718	6140	8340	6703	6807	8901	14070	8606	6746	8720
PC	PC 33:1	746.56378	[M+H]+	4.663	166413	94387	104415	99645	257876	374365	174390	264035	277242	157691	254663	270086	329057	256133	483433	421222
PC	PC 33:2|PC 15:0_18:2	744.55328	[M+H]+	4.114	269319	521473	460864	451225	711322	850961	589255	850610	1083861	758157	910965	1096306	1429364	799697	1217939	1541730
PC	PC 33:3|PC 15:1_18:2	742.5379	[M+H]+	4.41	395118	353938	710540	336037	284406	527450	369415	405639	517458	403311	327068	747980	685637	449566	710427	697797
PC	PC 33:5	782.49231	[M+HCOO]-	6.787	1723	1702	2676	2035	2317	2002	1588	1909	2574	2047	1869	2330	3031	2203	2413	2715
PC	PC 34:0|PC 16:0_18:0	762.59747	[M+H]+	5.742	369058	391445	499918	296440	414290	558116	261110	630617	493601	303271	599358	550514	757829	910253	396695	454564
PC	PC 34:1	804.56818	[M+HCOO]-	7.599	8180	29916	15136	79499	288645	81405	69669	3311	5567	3293	5753	162731	9768	3429	335433	377175
PC	PC 34:1|PC 16:0_18:1	760.58618	[M+H]+	4.987	19225684	18397824	22061652	8323874	17775616	19804830	11082848	20312892	19399284	14212470	19077768	19181252	23461502	23286148	26840794	17657940
PC	PC 34:2	780.54724	[M+Na]+	4.42	649426	855244	52397	1105364	768109	695640	772519	1053233	976682	840847	880182	1011817	767729	896341	1053447	950331
PC	PC 34:2|PC 16:0_18:2	758.57135	[M+H]+	4.418	123724968	204640448	192403136	163880624	178955856	175236800	152126896	211293712	242837040	180462272	210860800	229523104	255778848	217583568	215083072	218410704
PC	PC 34:3	756.55206	[M+H]+	4.292	15094	17093	3913299	12121	19676	21653	22917	17849	14117	20666	14911	11636	21927	26651	34219	26653
PC	PC 34:3|PC 16:1_18:2	756.55261	[M+H]+	3.848	1183257	2392895	1092	1428131	1754248	1681548	1217828	2018714	2693014	584244	2290251	1844648	3628642	1198178	2223034	3970701
PC	PC 34:4	754.53467	[M+H]+	3.583	16421	16381	22753	14227	19007	27894	9692	23341	27371	21880	25937	26013	39174	13110	29403	32754
PC	PC 34:9	744.46521	[M+H]+	7.047	19312	17516	24822	17892	24426	18775	17330	22251	28991	25063	28202	26977	30598	24211	23578	29428
PC	PC 35:1	774.59338	[M+H]+	5.379	207820	91787	17957	75341	178622	288979	97383	232276	238763	167589	224396	255008	277580	154102	303951	274241

PC	PC 35:2	816.57513	[M+HCOO]-	7.211	19418	29288	19610	27645	27634	30771	41161	29240	37505	27231	32974	38508	40331	33776	39904	44282
PC	PC 35:2|PC 17:0_18:2	772.58209	[M+H]+	4.791	886963	1208891	787844	1001825	1347073	757166	1571848	1411605	1801477	1292525	1592158	1770957	2206120	1698754	2008087	2343608
PC	PC 35:3	770.56525	[M+H]+	4.19	95062	145411	109721	95624	140355	228063	146227	136861	213335	99535	165786	186022	234530	152271	329149	372476
PC	PC 35:5	766.55151	[M+H]+	4.984	172337	203665	0	245261	114277	182449	296095	180699	429603	229510	186785	145965	299945	264552	223796	178336
PC	PC 36:1	832.60809	[M+HCOO]-	8.005	109756	58812	52985	38155	66339	77798	59857	63527	68603	48266	85765	73120	71634	95808	59647	57354
PC	PC 36:1|PC 18:0_18:1	788.61554	[M+H]+	5.774	4136039	2230516	2250126	1380227	2680384	3653564	1808205	3435418	2851600	1888466	3669790	2976830	3086440	4255644	3114408	2319217
PC	PC 36:2	808.57776	[M+Na]+	5.151	586751	685906	310832	573419	609818	810819	535338	883011	706540	312434	616518	516455	614269	643732	561039	778457
PC	PC 36:2|PC 18:0_18:2	786.59991	[M+H]+	5.194	59993576	42760280	25659220	33305750	30933234	55301144	30788540	57082596	43007968	17106658	69196928	53592148	64501148	74490256	45858948	39833356
PC	PC 36:3	828.57611	[M+HCOO]-	6.977	222380	193553	183067	145872	180250	230355	169839	215331	244980	152505	208136	272682	269619	263143	286360	247066
PC	PC 36:3|PC 18:1_18:2	784.58264	[M+H]+	4.522	7965839	7491392	8038902	4904762	7381938	10148115	5281668	10503176	11686248	6118355	8834677	12027290	13205867	14619936	13015596	10844306
PC	PC 36:4	804.54694	[M+Na]+	3.96	138143	122465	845	84526	102883	175704	101568	139767	214202	108905	157587	240118	367455	217846	256654	299529
PC	PC 36:4|PC 16:0_20:4	782.56915	[M+H]+	4.292	3681292	2528015	6726018	2567792	2850886	5256394	2423657	4393810	3501042	2744868	3420206	4061246	4254370	3603140	3735538	4002854
PC	PC 36:4|PC 18:2_18:2	782.56854	[M+H]+	3.957	7182282	6136104	974	6818336	6978789	11610367	7707217	7970096	12770122	7116062	8412785	11480743	15501304	10416376	11912169	12904078
PC	PC 36:5|PC 18:2_18:3	780.55231	[M+H]+	3.499	92729	101637	88145	68654	88725	508385	67329	95725	285408	124504	139478	244123	208645	255870	518013	572073
PC	PC 37:2|PC 19:0_18:2	800.61212	[M+H]+	5.583	292898	191361	112691	130919	148172	335002	194787	295533	195442	148358	354810	258690	396875	380801	324239	285206
PC	PC 37:3	798.59302	[M+H]+	4.854	42123	35951	3027	10987	26065	35595	30352	29557	19569	22197	76291	19167	38631	44754	89316	85785
PC	PC 38:2	814.62958	[M+H]+	5.945	286260	112667	131498	52076	180504	179976	116196	205284	179398	130790	208118	214123	292540	208700	245826	172966
PC	PC 38:3	812.6134	[M+H]+	5.354	259908	106092	231818	136481	168156	313062	165950	327442	153992	130236	306972	230033	368854	342496	146704	218966
PC	PC 38:3|PC 20:1_18:2	812.60986	[M+H]+	5.197	373976	104740	151372	114662	220284	361427	163923	272695	147948	172915	274678	214036	385001	380988	276738	232945
PC	PC 38:4	810.59357	[M+H]+	4.628	400740	278666	10273	231542	157089	532818	198972	244565	204539	179820	175283	407112	755175	565852	372308	342690
PC	PC 38:4|PC 18:0_20:4	810.59619	[M+H]+	5	874684	441204	271181	519964	551262	1004202	412787	835905	601579	616959	986961	647235	793288	834147	483071	529985
PC	PC 38:5	808.58301	[M+H]+	4.333	377715	206936	1448	200688	254622	525677	189494	343466	278339	259036	303479	399895	397409	346994	488242	386589
PC	PC 38:6	806.56689	[M+H]+	4.512	169700	141541	262603	109851	212935	176128	88903	107807	93319	89903	192364	276963	101516	266560	305451	252723
PC	PC 38:6|PC 18:2_20:4	806.56812	[M+H]+	3.807	504679	251209	165	190259	199586	485525	165435	327050	319541	206858	325983	401695	383757	324586	339534	405438
PC	PC 38:7	804.547	[M+H]+	4.349	62868	54594	138924	47978	51828	73167	36721	81742	64765	43541	78732	9136	90230	64021	62672	80703
PC	PC 38:8	802.54559	[M+H]+	4.387	2905	2391	1577	1886	2992	2180	4359	1329	2956	1841	4434	1661	3494	3062	5970	3504
PC	PC 40:10	826.53296	[M+H]+	4.471	12176	5866	704	18986	20304	4469	5648	11071	9304	6988	10425	7311	4942	29987	14571	11020

PC	PC 40:5	836.61102	[M+H]+	5.104	71131	8391	35443	15489	10077	62160	15701	11256	8959	14909	11456	15206	65755	7656	21704	45451
PC	PC 40:6	834.59631	[M+H]+	4.397	39710	16772	978	28551	20883	16263	10253	20174	30454	33726	28874	27796	43606	35303	40106	21717
PC	PC 42:10	854.56366	[M+H]+	5.164	8237	3546	476	3421	5687	5023	7181	3810	5949	4938	3044	10163	17118	6201	3225	3698
PC	PC 15:4_24:6	856.51947	[M-H]-	7.288	1481	555	1011	837	1470	1399	887	1492	2655	1792	1515	1574	2967	1634	2551	2264
PC	PC 44:0_7:0	1044.85583	[M-H]-	6.984	226309	252375	254173	244064	252051	255760	243004	242595	241887	258784	254766	257567	239218	252820	266473	240381
EtherPE	PE O-32:1|PE O-16:1_16:0	674.51398	[M-H]-	7.4	5944	5874	3601	4467	3627	7281	4939	5622	5085	3166	4744	5401	6802	4886	3584	5779
EtherPE	PE O-32:2	674.50684	[M+H]+	4.591	20396	11550	14257	7818	22153	34959	14792	29320	11227	9143	14596	9788	22583	9106	25082	22957
EtherPE	PE O-32:2|PE O-16:1_16:1	672.49188	[M-H]-	6.972	2794	3282	2474	2972	3170	4571	3385	4052	1204	1579	2502	2508	2896	2334	4540	3161
EtherPE	PE O-33:3|PE O-15:1_18:2	684.49304	[M-H]-	6.855	2778	3302	1303	2903	1819	4561	3756	3805	4668	1109	3601	4661	6707	2833	6241	5451
EtherPE	PE O-34:1	704.55365	[M+H]+	5.275	132312	125350	13090	60414	144497	173286	79855	106004	122257	83417	102140	132388	150372	125850	145687	151360
EtherPE	PE O-34:1|PE O-18:1_16:0	702.54095	[M-H]-	7.801	7766	4091	101	2578	2217	6488	5994	213	4442	1329	20375	6754	6483	3913	253	4596
EtherPE	PE O-34:2|PE O-16:1_18:1	700.53101	[M-H]-	7.436	140401	125233	76482	67617	91330	109110	87869	95284	90424	51605	88587	81000	92380	82769	104698	83997
EtherPE	PE O-34:3|PE O-16:1_18:2	698.51361	[M-H]-	7.081	482662	499614	493169	476441	454273	618605	477507	472418	643299	310355	410414	580419	684224	315908	741203	623224
EtherPE	PE O-34:4	698.50684	[M+H]+	4.441	6157	702	15638	6923	3583	4211	2326	1884	22226	591	2549	2918	18158	1265	32946	30240
EtherPE	PE O-34:4|PE O-16:2_18:2	696.49445	[M-H]-	6.731	4112	2784	4074	4196	5124	8772	3577	3511	6995	3334	3883	4818	4392	3268	10072	8675
EtherPE	PE O-34:5|PE O-14:1_20:4	694.47906	[M-H]-	6.469	1774	1440	1543	1255	1409	2063	855	1835	1683	1092	1675	1639	2377	1806	2109	1545
EtherPE	PE O-35:1	718.56537	[M+H]+	4.892	74290	42462	1762	33969	88107	124577	87033	64836	56473	21912	38180	18527	38807	12087	79706	69500
EtherPE	PE O-35:2	716.55591	[M+H]+	4.675	81669	95074	84595	11663	38265	58103	25286	70522	47514	12674	65117	31712	62094	35026	48089	17368
EtherPE	PE O-35:2|PE O-17:1_18:1	714.54364	[M-H]-	7.652	7771	8982	3414	3857	7046	6759	5722	5367	4480	2880	6709	3349	4169	7038	6256	5197
EtherPE	PE O-35:3|PE O-17:1_18:2	712.52808	[M-H]-	7.322	42658	43986	29819	38819	39395	48503	50942	33510	44620	26493	41078	48215	55051	33583	59458	52317
EtherPE	PE O-35:4	712.51947	[M+H]+	4.496	6471	8774	20188	17502	16859	10184	9453	4544	28303	7865	9458	21859	12025	5807	27686	38457
EtherPE	PE O-35:5|PE O-15:1_20:4	708.49817	[M-H]-	6.729	1354	1343	1515	1264	1494	3100	1310	1995	2851	1428	2069	2406	2912	2248	2350	3313
EtherPE	PE O-36:2	730.56274	[M+H]+	5.249	9424	480598	19113	6269	460887	9763	10240	357364	20958	12007	502735	507643	14653	396904	455140	369577
EtherPE	PE O-36:2|PE O-18:1_18:1	728.56305	[M-H]-	7.862	60525	72599	30359	26916	44646	32600	37693	38236	37031	24701	46705	30969	33911	44130	39536	28243
EtherPE	PE O-36:3|PE O-18:1_18:2	726.54639	[M-H]-	7.542	1012805	1228495	866392	910027	976992	842789	1016312	837639	1104427	727785	1129184	1211981	1270964	942770	971219	915633
EtherPE	PE O-36:4|PE O-18:2_18:2	724.53125	[M-H]-	7.128	755756	945326	772856	737063	795395	766087	958332	793095	998109	573839	916920	1027271	1077189	758955	925038	921644
EtherPE	PE O-36:5|PE O-16:1_20:4	722.51495	[M-H]-	6.986	428299	404180	509453	384065	412922	509167	293708	501550	538135	328589	415565	502163	528967	393477	469191	443446
EtherPE	PE O-36:5|PE O-18:3_18:2	722.51294	[M-H]-	6.742	132461	194410	142530	147513	105372	87999	156726	95960	154037	87411	136760	157720	221677	113284	80672	124060

EtherPE	PE O-36:6	722.50586	[M+H]+	4.727	88105	84111	63133	84418	82878	123129	86318	78874	94148	67405	64781	92922	104537	54793	140607	110255
EtherPE	PE O-36:6|PE O-16:1_20:5	720.49561	[M-H]-	6.647	3644	3074	4789	5367	3552	12865	2633	5035	5060	3722	4060	5381	7712	4330	14908	10218
EtherPE	PE O-36:6|PE O-18:4_18:2	720.49493	[M-H]-	6.372	2608	2460	3802	4173	3064	5550	3083	2122	3938	2174	2905	3984	6214	2864	5923	6279
EtherPE	PE O-37:1|PE O-19:0_18:1	744.5838	[M-H]-	7.619	2080	1540	353	1902	386	374	1140	1154	1045	501	378	400	478	441	1313	942
EtherPE	PE O-37:5	738.53931	[M+H]+	4.003	9439	17452	279	3331	14719	2221	5753	23752	18608	12100	23503	26003	14241	40357	3466	6402
EtherPE	PE O-37:6|PE O-17:2_20:4	734.50861	[M-H]-	6.872	1141	1639	1929	1656	2166	3188	1795	2315	2590	1819	2637	2166	3861	2814	4267	4187
EtherPE	PE O-38:3|PE O-18:1_20:2	754.57837	[M-H]-	7.919	31456	21510	13503	14847	21920	18681	17656	17758	19735	15389	21472	25256	24368	24587	15879	16517
EtherPE	PE O-38:3|PE O-20:1_18:2	754.57404	[M-H]-	7.399	14256	11599	9976	10907	17104	11589	9707	7784	15432	9192	14448	10974	11627	9753	6215	10173
EtherPE	PE O-38:4|PE O-18:1_20:3	752.55969	[M-H]-	7.66	57287	60291	49026	57984	62288	71954	48923	64204	60310	47655	76299	64159	83767	80280	66183	58894
EtherPE	PE O-38:5|PE O-16:1_22:4	750.5451	[M-H]-	7.321	71911	51084	53037	49100	57853	105151	54580	38619	58945	36784	49815	35507	57031	52624	97811	45842
EtherPE	PE O-38:5|PE O-18:1_20:4	750.54492	[M-H]-	7.451	855787	899948	884198	704523	795539	713579	629224	859666	935549	775742	1073870	971093	937041	1146788	586599	662405
EtherPE	PE O-38:6|PE O-18:2_20:4	748.53271	[M-H]-	7.032	584083	653481	720661	515388	604484	591785	537529	751674	767329	561134	779474	752783	772823	822867	564815	626203
EtherPE	PE O-38:6|PE O-18:3_20:3	748.52594	[M-H]-	6.822	9540	14410	12713	12309	11615	783	10059	9394	3774	5555	4969	1538	22949	3000	7131	6273
EtherPE	PE O-38:6|PE O-20:5_18:1	748.52606	[M-H]-	6.35	3924	2489	4796	2447	3882	3011	3021	3354	3979	2827	2304	4784	3378	2173	2740	2882
EtherPE	PE O-40:4|PE O-18:0_22:4	780.58746	[M-H]-	7.824	6756	8427	5059	6531	8247	6278	5610	5332	7463	8124	13755	2801	4987	4744	6414	3092
EtherPE	PE O-40:5|PE O-18:1_22:4	778.57745	[M-H]-	7.751	67022	46823	35373	40267	52012	38748	47104	32736	47065	42937	44263	37297	39933	39787	33599	29247
EtherPE	PE O-40:6	778.56714	[M+H]+	5.105	143490	102405	9544	89135	152302	140572	100010	108534	148683	126099	138315	125258	147667	117343	132205	134873
EtherPE	PE O-40:6|PE O-18:1_22:5	776.5575	[M-H]-	7.459	56736	41544	47539	57599	19919	65455	46778	47043	60996	49091	52694	64015	73517	16312	63155	56292
EtherPE	PE O-40:7|PE O-18:2_22:5	774.54761	[M-H]-	7.06	44002	31127	40550	38906	36347	51134	39240	36444	48696	35037	41853	52836	59020	47412	49866	46606
EtherPE	PE O-40:8|PE O-18:2_22:6	772.52625	[M-H]-	6.892	16035	21148	18838	19336	15688	17750	17487	23145	27569	12780	22360	24103	32861	27652	22973	21268
EtherPE	PE P-32:0|PE P-18:0_14:0	676.52032	[M+H]+	5.148	25176	32174	4320	25541	21343	38252	4571	29425	19142	10710	28996	34330	39669	30288	7283	33684
EtherPE	PE P-34:1|PE P-16:0_18:1	702.54095	[M+H]+	5.206	443150	300496	202770	159119	298531	412341	155563	391769	320346	151730	368969	320562	397753	348896	379020	224314
EtherPE	PE P-34:2|PE P-16:0_18:2	700.52667	[M+H]+	4.71	2420692	2656714	583	2123063	2526116	3705656	2000319	2642736	3509299	1439133	2289095	3244676	4173165	1582000	4494673	3601555
EtherPE	PE P-35:1|PE P-18:0_17:1	716.55627	[M+H]+	5.539	15616	19256	20078	20149	17255	8164	16958	21494	22885	8413	26165	3006	24021	28163	32921	17602
EtherPE	PE P-35:2|PE P-17:0_18:2	714.53705	[M+H]+	5.046	138711	184085	141314	167223	153260	236654	241118	163501	220669	144006	188584	228245	284942	158038	272736	228452
EtherPE	PE P-36:1|PE P-18:0_18:1	730.57306	[M+H]+	5.824	179402	230648	112867	85568	167368	114493	104083	153768	132158	91652	173958	119640	128014	174096	150560	99829
EtherPE	PE P-36:2|PE P-18:0_18:2	728.55743	[M+H]+	5.359	4434672	6499956	4613842	4164461	5609804	4405904	4472072	3973138	5907325	3669541	5994360	6701528	7275238	4961805	4770182	4748569

EtherPE	PE P-36:3|PE P-18:1_18:2	726.54108	[M+H]+	4.758	3173702	4525632	12507	2968806	4200464	4216840	4276723	3887344	4564128	2717358	4541226	4695108	5778906	3434800	4975137	4839092
EtherPE	PE P-36:4|PE P-16:0_20:4	724.52527	[M+H]+	4.586	1657108	1583556	568215	1383595	1893522	2393018	900479	2264774	2366402	1375826	1799196	2147397	2331482	1752538	2007581	1843675
EtherPE	PE P-36:4|PE P-18:2_18:2	724.52667	[M+H]+	4.261	527357	924243	761	658067	562660	438641	640702	498969	804701	440571	693849	832060	1252311	601027	419147	617751
EtherPE	PE P-37:4|PE P-17:0_20:4	738.53668	[M+H]+	4.911	128454	136962	57410	129244	124702	177871	98798	161813	173726	68534	176394	173589	169395	188728	179492	174733
EtherPE	PE P-38:3|PE P-18:0_20:3	754.57172	[M+H]+	5.532	198742	218409	184198	184197	257383	275108	145313	265765	213585	185079	309215	249866	337263	328840	246283	220190
EtherPE	PE P-38:4|PE P-18:0_20:4	752.55701	[M+H]+	5.228	3000044	3567248	727541	2506995	3764918	2836180	1986392	3724647	3919748	3484668	4831745	4211226	3927024	5309648	2201102	2646167
EtherPE	PE P-38:5|PE P-18:1_20:4	750.5426	[M+H]+	4.652	3229837	3397988	97722	2424526	3905875	3811834	2385709	4169901	4281662	2928582	4799751	4599520	4704280	4716484	3600461	3782531
EtherPE	PE P-38:6|PE P-18:2_20:4	748.52667	[M+H]+	4.141	349416	516710	5758	374375	349367	333486	252557	447970	453327	387092	544161	530521	758722	624354	279933	373723
EtherPE	PE P-40:4|PE P-18:0_22:4	780.58612	[M+H]+	5.644	188362	130298	127773	106499	188803	151547	118249	133056	162722	133051	169363	144300	134798	161620	144967	113157
EtherPE	PE P-40:5|PE P-18:0_22:5	778.56744	[M+H]+	5.262	285368	239305	280438	288676	311799	366995	208474	292586	357783	300018	324593	392891	446971	417699	370236	327687
EtherPE	PE P-40:6|PE P-18:1_22:5	776.5528	[M+H]+	4.671	222285	210271	12288	225318	246039	340092	167053	269456	297939	227892	255193	342573	416008	329032	366897	322474
EtherPE	PE O-11:0_28:0	774.63837	[M-H]-	6.914	28721	28147	18385	27890	27198	27818	20136	20205	19309	28192	19431	18454	18331	19563	18436	15211
EtherPE	PE O-15:3_28:7	810.54614	[M-H]-	6.497	3965	65	699	32	491	37	1077	313	169	583	664	668	1149	370	333	751
EtherPE	PE O-16:2_22:6	744.49487	[M-H]-	6.274	2527	2277	4274	3822	2397	5254	2040	2858	3386	2596	2936	4057	5650	4183	4646	5316
EtherPE	PE O-16:3_24:6	770.51141	[M-H]-	6.498	1371	2020	2328	2704	1402	1768	1720	2210	2414	1774	2247	2327	3382	2946	1866	2305
EtherPE	PE O-8:0_24:3	670.47681	[M-H]-	6.562	2001	1809	1454	1339	1759	2366	1322	1479	2186	1202	1689	2361	3135	1780	3132	2100
EtherPE	PE O-8:0_26:0	704.56134	[M-H]-	7.896	2477	1743	1467	1252	1421	3168	1776	1400	2161	1220	1845	1684	990	1943	1494	2163
EtherPE	PE O-8:0_28:1	730.57074	[M-H]-	7.857	700	131	137	67	158	374	1275	1343	213	747	108	95	447	296	82	341
EtherPE	PE O-8:0_28:2	728.55847	[M-H]-	7.363	19473	13014	12554	13355	16570	15512	11565	10811	17107	10692	17673	19021	14743	12957	10901	7685
EtherPE	PE O-9:0_26:4	710.51074	[M-H]-	6.897	2385	4649	4079	3309	4252	5683	4878	4183	5763	1945	3759	5488	7470	2981	7390	7196
EtherPE	PE O-9:0_28:0	746.60535	[M-H]-	6.483	678770	629604	674151	1087439	1204181	729260	626707	1206622	1207365	608979	644632	735284	708809	834052	785595	702044
EtherPE	PE O-9:0_28:3	740.55664	[M-H]-	7.754	7232	10499	4856	7478	6115	7255	7845	5736	5695	4442	10101	10981	13176	9518	12238	7967
PE	PE 34:1	718.54266	[M+H]+	4.815	474	74997	2804	54074	11768	12060	1746	70430	20321	31110	32027	78909	93165	96931	52522	54131
PE	PE 34:1|PE 16:0_18:1	716.52405	[M-H]-	7.162	14642	14485	18986	10867	11694	19499	10566	13154	12188	7636	11628	17058	19483	18089	17179	17570
PE	PE 34:2|PE 16:0_18:2	716.52747	[M+H]+	4.333	163530	160309	369264	213018	288080	261349	148269	261980	326721	230883	210863	288597	477764	278301	329629	412056
PE	PE 34:3|PE 16:1_18:2	712.487	[M-H]-	6.333	2178	2050	5004	2005	1374	2589	2095	1104	2053	1075	1199	1880	2909	1301	2875	2221
PE	PE 35:2	728.52136	[M-H]-	7.043	8239	6741	9231	7971	9534	12203	14677	7226	13514	9495	9164	14411	13378	8968	12545	20069
PE	PE 36:1|PE 18:0_18:1	744.55835	[M-H]-	7.611	36094	27133	30320	22394	28436	47816	26276	27979	30022	18151	31344	33234	34314	36207	32038	36073
PE	PE 36:2|PE 18:0_18:2	742.54077	[M-H]-	7.275	1556556	1420766	1732373	1460068	1526606	1664750	1598864	1453750	2094274	1344356	1663062	2155382	2049075	1719660	1486018	1940362

PE	PE 36:4|PE 16:0_20:4	738.50671	[M-H]-	6.71	10947	8489	20063	8739	11662	12653	7693	11499	14774	15791	11088	14536	17795	13063	11729	12994
PE	PE 36:4|PE 18:2_18:2	740.52179	[M+H]+	3.9	225815	176863	217668	145892	235276	299550	166607	186847	504011	209560	171887	418280	487113	223105	335374	433081
PE	PE 37:2|PE 19:0_18:2	756.55206	[M-H]-	7.501	11813	11081	9788	10658	10089	13294	17702	11398	12708	9683	13113	17233	18239	13552	17409	23547
PE	PE 37:4	754.53247	[M+H]+	4.336	20427	25068	19828	16472	3051	27652	17877	22625	20602	9736	22897	30631	33412	2951	38244	33667
PE	PE 37:4|PE 17:0_20:4	752.51904	[M-H]-	6.951	3550	1022	3008	302	2094	4260	3264	2563	4549	3261	3006	2399	4039	3051	3281	4008
PE	PE 38:3|PE 18:0_20:3	768.5553	[M-H]-	7.398	46980	29391	32632	32139	32114	61445	33256	35894	43324	29296	42751	46631	55632	46646	38496	43753
PE	PE 38:3|PE 20:1_18:2	768.54987	[M-H]-	6.951	9333	9296	9477	7403	7950	9230	6512	9861	11358	5972	4310	11888	6745	9951	12163	4971
PE	PE 38:4|PE 16:0_22:4	766.53461	[M-H]-	6.886	12522	9920	12049	8268	8687	15330	9432	8875	11867	8297	7851	13685	22475	11491	11547	19231
PE	PE 38:4|PE 18:0_20:4	766.54419	[M-H]-	7.199	976288	395286	639673	360835	386569	761898	489532	385588	725496	660563	661300	700709	711898	681348	421850	475605
PE	PE 38:5	764.52618	[M-H]-	6.759	11280	7290	15524	7123	5722	15391	7935	8065	13164	13240	9942	15541	16185	10332	13322	10852
PE	PE 38:5|PE 18:0_20:5	764.51965	[M-H]-	6.894	8839	5705	9621	6520	6388	20946	6316	6752	10136	9047	10065	10270	15096	10864	15585	14124
PE	PE 38:6	764.53766	[M+H]+	4.382	13997	9189	1099	5856	3758	8470	10189	10672	14503	12690	8656	17689	6365	2946	6754	12832
PE	PE 39:4|PE 19:0_20:4	780.55481	[M-H]-	7.417	13691	8835	9056	8225	8161	12372	12136	8522	10088	10116	11224	11934	14016	11243	11629	11570
PE	PE 39:6	778.54523	[M+H]+	4.477	10251	11703	994	16577	13552	7656	9824	16983	9657	5397	22190	19769	47199	10280	19565	28524
PE	PE 40:4	796.59357	[M+H]+	5.297	15876	5622	37362	26875	9046	15511	12697	7750	8351	5675	0	87306	42716	11025	9777	57150
PE	PE 40:4|PE 20:2_20:2	794.56665	[M-H]-	7.552	5475	7040	10179	8220	4471	4727	6579	13331	10894	8047	10702	14547	11498	3935	7628	3585
PE	PE 40:5|PE 18:0_22:5	792.55481	[M-H]-	7.363	26363	16176	14965	13909	12878	17344	13440	21531	27682	15206	29733	28743	22541	28063	11790	12870
PE	PE 42:0_6:0	914.75934	[M-H]-	9.597	3767	2816	1176	2816	1400	759	2172	1752	287	573	479	122	982	502	127	256
PE	PE 6:0_42:1	912.74371	[M-H]-	9.433	2898	2330	1046	2835	928	617	1391	1295	232	415	500	229	1114	107	160	306
PEtOH	PEtOH 34:2	699.49841	[M-H]-	6.38	70	14	17	9	30	7410	11	14	20	7	66	12	579	34	31	5686
PEtOH	PEtOH 43:10	809.50647	[M-H]-	7.135	14140	18591	16584	16844	16716	15849	18782	16689	17436	13484	17568	17147	17751	16144	18544	18006
PEtOH	PEtOH 27:0_28:0	997.87097	[M-H]-	6.986	5738	5892	3786	6610	5856	5845	5500	3551	6202	6199	5855	5393	5290	5577	2563	5806
PEtOH	PEtOH 28:0_28:0	1011.87286	[M-H]-	8.251	13339	15182	13033	14067	14030	14326	14598	12745	14544	12225	13578	14312	13158	13058	13243	10855
EtherPG	PG O-12:0_28:7	805.53656	[M-H]-	7.266	1635	1835	3106	996	2555	3153	3994	4412	2523	2695	3608	5462	3827	2272	3737	4502
EtherPG	PG O-13:0_28:6	821.56451	[M-H]-	7.371	23450	18693	23254	11038	14977	16597	14835	17824	18257	13881	17131	15283	18835	25044	28527	23570
EtherPG	PG O-13:0_28:7	819.54889	[M+FA-H]-	6.893	89001	129097	127662	114695	109006	110277	113103	122994	133520	119887	122313	124689	138215	117960	126728	129517
EtherPG	PG O-14:0_28:2	843.65222	[M-H]-	8.103	243	1732	589	628	235	952	1195	5772	1296	510	1528	1545	2383	1690	993	994
EtherPG	PG O-15:0_28:3	855.64758	[M-H]-	7.868	56	587	1462	86	1288	237	60	1395	627	1417	1574	2011	22493	10185	1702	8857
EtherPG	PG O-15:0_28:6	849.5976	[M-H]-	8.01	12761	8244	5387	3621	5595	8626	7128	7900	8041	2459	6119	7395	6385	8238	9334	4470

EtherPG	PG O-15:0_28:7	847.58215	[M-H]-	7.511	27627	19020	17250	19298	16510	28218	20988	22529	23150	43203	28321	54403	29110	35298	26719	21051
EtherPG	PG O-16:1_28:7	859.58789	[M-H]-	8.681	11670	12890	8790	8220	12488	12000	11924	8304	12694	13275	11118	12649	6176	12801	11961	10178
EtherPG	PG O-16:4_26:7	825.50208	[M-H]-	6.84	4071	2655	3610	2491	3492	4956	3336	3560	5168	3665	3305	5099	4564	2585	4850	4303
EtherPG	PG O-25:0_28:0	1001.84369	[M-H]-	8.256	3207	3295	2424	3080	2915	3314	3831	3149	3125	3110	3265	3196	2306	2888	2531	2275
EtherPG	PG O-27:0_28:1	1027.86926	[M-H]-	6.984	141814	136560	136953	131115	133792	137806	135233	136139	140639	127935	136722	138848	135174	130653	132435	129214
EtherPG	PG O-9:0_28:0	777.60083	[M-H]-	7.198	2587	10703	7586	8772	7389	7537	7703	16216	10151	6902	8607	10977	11906	12189	7203	8570
PG	PG 34:2	764.55969	[M+NH4]+	6.652	908	458	42181	2651	667	380	723	7150	2981	5175	352	6302	444	435	919	388
PG	PG 36:3|PG 18:1_18:2	771.52051	[M-H]-	5.662	2761	1744	562	1239	1892	1111	1511	1425	1434	815	1765	2400	1741	1716	771	1066
PG	PG 19:0_38:0	1071.89355	[M-H]-	6.945	1457	1801	233	1186	1016	1272	1926	367	1480	1965	1722	1555	762	928	1204	1101
PG	PG 6:0_30:2	773.53424	[M-H]-	6.498	14615	10669	5117	5486	11469	10811	6814	9727	10617	9752	11071	10783	13751	11352	11311	9261
PG	PG 6:0_32:4	797.53503	[M-H]-	6.433	1288	1772	1817	1412	1593	945	868	2513	2125	1529	2151	1829	1713	1819	1034	1397
EtherPI	PI O-8:0_17:0	697.43182	[M-H]-	5.734	2085	2223	2010	1725	1794	1938	1680	1741	2027	1734	1663	2241	1584	1977	1948	1759
EtherPI	PI O-8:0_24:1	793.53528	[M-H]-	6.903	12141	10983	9019	7178	14771	13719	15621	7300	15727	13203	9478	7445	9928	13937	15419	11501
EtherPI	PI O-8:0_28:3	845.55939	[M-H]-	6.966	12612	11644	10887	6616	10665	12332	10446	11867	12286	9094	8410	14312	10915	9019	13541	11549
EtherPI	PI O-9:0_26:7	823.48315	[M-H]-	6.447	2243	2173	2458	1808	2369	2855	2172	2007	4060	2202	1865	3323	3349	2167	2700	3338
EtherPI	PI O-9:0_28:7	851.51532	[M-H]-	7.198	11567	7549	11682	7590	8549	12939	8839	7815	9372	13685	9960	8927	9403	9941	9684	10205
PI	PI 30:0	800.5448	[M+NH4]+	5.168	19641	8255	16945	4644	8016	24145	9407	3042	6995	4438	5339	7728	2867	11239	21460	7308
PI	PI 34:0	837.5517	[M-H]-	6.648	13055	2887	4316	2865	5833	5836	8195	3448	3929	3701	4436	10641	5239	5267	3167	3469
PI	PI 34:1	835.53107	[M-H]-	6.216	2536	1783	2599	1081	2857	3191	3978	1380	985	1487	1455	5352	2831	2402	2550	2899
PI	PI 36:1	863.56897	[M-H]-	6.69	10036	3200	3708	2356	8768	5869	6671	4176	4395	3453	3351	9414	4564	5845	4945	5628
PI	PI 38:2|PI 18:0_20:2	889.58557	[M-H]-	6.76	9978	1584	764	1219	4167	4104	3472	1705	1901	2153	1803	4407	3783	3922	2967	4054
PI	PI 38:3	887.56769	[M-H]-	6.482	18262	3846	5341	3971	8628	8832	7677	6272	3888	4216	5027	8778	9369	7380	5977	6809
PI	PI 38:4	885.55304	[M-H]-	6.294	139404	54183	51103	24044	70214	57081	72312	53499	33095	43980	58742	87509	73133	76303	46695	54561
PI	PI 40:4	913.58405	[M-H]-	6.622	4279	1148	804	1037	2400	2149	2282	1889	1231	1382	1123	2716	2008	2169	1572	1799
PI	PI 40:5	911.56677	[M-H]-	6.307	3032	866	1667	1344	2069	1976	1882	1960	1430	1664	1685	3440	3197	3116	2193	1948
PI	PI 18:0_38:2	1141.84448	[M-H]-	7	5375	3870	2881	7178	3115	6195	6017	4766	2604	3959	4501	4401	4107	3409	5049	3183
PI	PI 6:0_34:3	915.59808	[M-H]-	5.293	3444	3308	3318	3260	3173	3209	3001	3150	3643	2844	3429	3422	3282	3530	3652	3383
EtherPS	PS O-8:0_16:2	604.35938	[M-H]-	4.033	392	165	156	46	237	29	134	132	24	641	890	3446	276	976	588	976
PS	PS 36:0	790.55945	[M-H]-	7.096	4055	4728	2865	2602	2941	8134	5008	4656	4884	2948	5288	4587	6425	5254	8058	8900
PS	PS 36:0|PS 18:0_18:0	790.54297	[M-H]-	7.143	6937	2190	6672	1702	3205	10125	4431	5711	9320	4267	6250	9032	9508	8450	10071	10677

	PS	PS 36:1|PS 18:0_18:1	788.54248	[M-H]-	6.642	10057	20851	17465	16347	23258	25327	25967	25138	30044	22597	26397	32072	37774	26362	33166	39820
	PS	PS 36:2|PS 18:0_18:2	786.53192	[M-H]-	6.569	65068	54330	37777	21632	65969	36670	62577	50969	36869	35170	51052	59206	72410	58340	48201	44916
	PS	PS 36:2|PS 18:1_18:1	788.53845	[M+H]+	4.025	150733	133675	158	58640	197296	114813	119326	148555	115328	102416	156991	174767	115053	187751	148170	144581
	PS	PS 38:2	816.5827	[M+H]+	4.832	38230	28872	13775	48901	86340	48793	15985	74011	35375	39360	71408	37181	26279	81018	36156	21555
	PS	PS 38:4	812.53961	[M+H]+	4.297	17744	1137	9935	1496	1450	1211	1686	7354	5235	1613	991	2115	2396	11771	4754	1327
	PS	PS 40:4	840.56824	[M+H]+	4.446	11098	17477	161	4177	33690	19612	13396	23710	11965	9585	28956	8772	24892	11383	19026	11732
	PS	PS 40:5	836.54266	[M-H]-	6.508	1851	1732	1272	1842	2421	2578	2593	3696	3265	2353	2387	5615	5454	5210	4162	3267
	PS	PS 40:6	834.53058	[M-H]-	7.197	15078	7727	10994	7588	7420	13873	8364	6966	10804	12832	9262	10256	10520	10317	8003	8697
	PS	PS 16:4_24:6	826.4704	[M-H]-	7.546	3015	2789	1606	2050	1846	2145	2414	1872	2816	1281	2240	2861	3102	2430	2155	1887
	PS	PS 18:5_22:6	824.45374	[M-H]-	7.131	1292	2347	1605	1198	1573	1409	2505	1680	2515	853	2142	2550	3148	1203	1861	2321
**Fatty acyls**	CAR	CAR 20:0	456.39984	[M+H]+	2.446	3061	28172	51065	34682	24898	1496	1206	13302	48355	7468	8818	69416	6539	41903	1008	3182
	CAR	CAR 22:1	482.41589	[M+H]+	2.478	1788	41546	81086	47129	14168	609	1190	20796	58630	8199	3594	35222	3453	20989	407	2370
	FA	FA 14:0	227.20117	[M-H]-	2.968	19450	19530	16237	16925	22399	15435	18878	15303	13209	8762	10362	10942	17073	12182	10445	10040
	FA	FA 15:0	241.21648	[M-H]-	3.507	8234	4804	6522	7900	7525	7870	7304	7371	7592	5976	7153	6296	8321	6723	6836	6295
	FA	FA 16:0	255.2338	[M-H]-	4.02	1806081	1808094	1781058	1664426	1950866	1468360	1484602	1625549	1808072	1179240	1416577	1437405	1822299	1623326	1410361	1341359
	FA	FA 16:1	253.21716	[M-H]-	3.176	96581	85542	70180	76052	124688	46126	85790	56435	33680	25701	16786	29867	70954	28218	13875	17461
	FA	FA 17:0	269.24783	[M-H]-	4.535	11882	11567	10092	11531	9808	10840	10791	9109	11936	8278	9273	8539	10792	10601	9605	6531
	FA	FA 17:1	267.23248	[M-H]-	3.685	6520	7638	5316	4502	6507	2139	2838	3738	3628	1037	3950	2195	4192	3420	1329	2419
	FA	FA 18:0	283.26544	[M-H]-	5.026	1460108	1359210	1487489	1415435	1284177	1270842	1135854	1470715	1848004	1067406	1424693	1299003	1801964	1635428	1331674	1241317
	FA	FA 18:1	281.2496	[M-H]-	4.19	1960206	2294494	1863782	1624873	2690209	1348997	1499866	1464267	1284084	861353	1156231	1124154	1899794	1183038	889344	743716
	FA	FA 18:2	279.23407	[M-H]-	3.44	1195632	1042489	936201	801064	835254	592916	570036	561502	759150	320677	514317	819792	1478918	575313	358606	343016
	FA	FA 18:3	277.21698	[M-H]-	2.792	14155	22211	19658	25348	25205	25136	14672	15540	20401	9291	12635	16019	25651	17635	7481	13882
	FA	FA 19:0	297.27792	[M-H]-	5.501	591	1195	1086	726	594	1465	757	1530	1330	445	527	819	2227	466	699	368
	FA	FA 20:0	311.29449	[M-H]-	5.912	11462	10448	11255	10605	10085	11378	9930	11447	13610	8458	9703	10345	13664	11182	10430	10247
	FA	FA 20:1	309.27994	[M-H]-	5.118	17249	13116	11321	11427	13755	12058	5892	10056	10361	7080	8791	11501	16381	8976	9482	7684
	FA	FA 20:2	307.26309	[M-H]-	4.398	15901	11305	14050	14396	14514	16382	8643	4673	14361	6217	8530	13156	25689	9511	8475	6968
	FA	FA 20:3	305.24786	[M-H]-	3.754	9448	7389	6397	15712	7218	8728	8271	12400	15513	3922	10558	7268	19161	8779	6254	5079
	FA	FA 20:4	303.23343	[M-H]-	3.266	150755	70827	113808	103528	71099	94561	25334	127610	135234	52132	112985	123195	188865	94077	60364	49884
	FA	FA 22:0	339.32559	[M-H]-	6.658	10223	6111	6981	6813	6605	9257	10408	8416	8277	5548	11075	6639	7304	6488	9232	9825
	FA	FA 22:1	337.30893	[M-H]-	5.941	3659	3658	2201	2520	1371	4214	2218	2707	3183	2073	2382	2797	3958	2603	2692	2655

FA	FA 22:4	331.26279	[M-H]-	4.065	15325	8535	13667	12147	12291	14646	10854	14466	19716	9451	11889	13749	20286	14192	11121	7953
FA	FA 22:5	329.2478	[M-H]-	3.439	6577	4705	10379	12837	6742	8847	4354	9467	14253	5789	8393	12611	24523	11729	8112	6130
FA	FA 24:0	367.35632	[M-H]-	7.288	22009	31791	29527	28298	24112	36938	17479	42638	26914	26826	23807	23634	32292	24146	30037	28675
FA	FA 24:1	365.33994	[M-H]-	6.656	2043	1959	1425	3084	1663	2104	1796	1894	1790	1270	1235	2215	2816	1890	1544	1191
FA	FA 25:0	381.37183	[M-H]-	7.507	16272	11416	25871	14215	16036	27413	13366	13460	28858	10979	26169	12625	17738	15245	24135	16195
FA	FA 26:0	395.38754	[M-H]-	7.799	45599	52136	55071	49044	48138	51505	44547	44648	51369	51249	49130	52040	53199	48683	53407	52925
FA	FA 27:0	409.40329	[M-H]-	8.014	9988	10905	8481	10478	11428	10022	8070	5412	11833	12024	11585	10715	11395	11755	11438	9964
FA	FA 28:0	423.42035	[M-H]-	8.244	19005	24341	22049	23784	21046	23406	17959	19108	23739	22075	22771	22365	24251	22915	21022	23550
FA	FA 29:0	437.4339	[M-H]-	8.447	4272	5104	5191	2811	5416	2811	3972	3847	5419	4194	3516	4580	4520	4029	5071	4641
FA	FA 30:0	451.45062	[M-H]-	8.629	9644	9448	8642	9864	9796	9685	9726	9748	9808	10822	10463	10138	9442	9233	11013	10817
FA	FA 32:0	479.48135	[M-H]-	8.955	2721	2727	2708	3137	2837	2618	3061	2666	3028	3279	3084	3325	2532	2941	3012	2569
FA	FA 32:1	477.4668	[M-H]-	8.581	3275	3063	2976	3471	2703	3312	3065	2975	2969	3220	3008	3018	2969	3411	3394	3142
FA	FA 40:5	581.52167	[M-H]-	7.369	4026	4330	5491	6497	5307	4334	5456	4773	5124	4666	4831	6347	4306	4795	5567	4280
FA	FA 42:5	609.5545	[M-H]-	7.798	5207	5608	5738	6939	6706	6164	6064	6888	6289	6063	7789	7984	5804	7235	6223	5638
FA	FA 44:5	637.59161	[M-H]-	8.17	3679	4512	3055	4200	4968	3679	3542	3885	4147	3299	4096	5609	3900	3205	3051	2007
FA	FA 12:0	199.16927	[M-H]-	10.045	236	206	124	178	208	205	205	115	103	219	132	103	3886	196	114	111
FA	FA 22:6	327.22977	[M-H]-	3.004	1406	1134	1202	2194	1173	1633	685	1860	2307	164	1651	2593	4023	2234	1614	1303
FAHFA	AAHFA 18:5/15:4;O	505.33136	[M-H]-	5.613	11777	8127	9326	8931	11391	7801	6689	12496	12058	7037	7363	9656	7288	10863	8694	11318
FAHFA	AAHFA 22:0/8:0;O	481.42395	[M-H]-	7.467	10808	12324	18917	15399	12053	17626	17724	10517	17273	18566	8800	15996	15002	11892	17201	17222
FAHFA	AAHFA 22:6/19:5;O	613.43372	[M-H]-	7.202	2601	2179	1984	1860	2180	2100	1687	1349	2461	2097	1665	2394	2303	2063	2045	1971
FAHFA	AAHFA 28:3/10:0;O	587.50574	[M-H]-	4.024	7793	8355	6865	6953	8942	6132	6322	7122	8172	5182	5995	6670	7311	6858	5835	6167
FAHFA	AAHFA 28:3/13:0;O	629.54773	[M-H]-	7.719	1697	2493	3205	2839	2516	3465	1754	1927	2915	1775	3111	1901	2704	2176	2670	2204
FAHFA	AAHFA 28:3/8:0;O	559.47302	[M-H]-	3.442	4761	3847	2645	2387	2207	1523	1381	1232	2011	605	988	2076	4940	1174	590	631
FAHFA	AAHFA 28:4/13:0;O	627.53143	[M-H]-	7.584	62771	105080	158796	104705	138455	86063	79919	67798	107577	83862	83183	121454	101417	71930	73117	61709
FAHFA	AAHFA 28:4/15:0;O	655.56366	[M-H]-	7.97	28285	7783	7270	6805	8283	16217	5563	6824	8759	8108	10696	11652	10562	7581	6659	5294
FAHFA	AAHFA 28:4/16:0;O	669.57904	[M-H]-	8.156	4089	2134	2109	2294	2527	2734	1494	2392	2298	2035	2408	2636	2705	2599	2138	1983
FAHFA	AAHFA 28:4/18:0;O	697.60986	[M-H]-	8.473	11256	9424	5393	6614	8827	10367	6640	9883	8104	7065	9033	11169	10224	10519	11251	6820
FAHFA	AAHFA 28:4/19:0;O	711.62488	[M-H]-	8.624	14077	10848	6777	9143	12123	15996	9238	11373	11024	9117	10019	12676	13180	13503	12640	8209
FAHFA	AAHFA 28:5/19:0;O	709.61053	[M-H]-	8.302	32101	27407	21789	22062	31479	31331	17876	27223	27445	19508	24119	35601	30327	28260	21721	16084
FAHFA	AAHFA 28:6/10:0;O	581.45013	[M-H]-	3.446	1924	1808	1365	1035	1155	687	631	682	1045	159	563	1033	3191	725	344	337

	FAHFA	AAHFA 28:6/22:2;O	745.60907	[M-H]-	4.041	81513	84320	78342	80807	78488	72668	70261	71135	82859	70719	72459	73198	78042	79568	50288	50589
	FAHFA	AAHFA 28:6/24:2;O	773.63629	[M-H]-	6.893	3456	1656	4036	3656	4435	3222	4108	3982	4794	4020	3714	3659	3031	4614	3570	2698
	FAHFA	AAHFA 6:0/15:2;O	351.24985	[M-H]-	5.031	13143	12173	13761	12697	11829	11797	10968	13521	16577	10003	13290	12075	15787	13814	12182	11327
	NAE	NAE 28:0	468.46875	[M+H]+	4.72	6957	8231	4868	7360	8139	6723	5623	5967	4516	7613	5060	9270	5317	6224	8462	7326
	NAGly	NAGly 11:0	242.17548	[M-H]-	3.983	1543	1686	599	3149	1286	772	1009	1627	1423	911	1259	1135	686	1529	1282	1640
	NAGly	NAGly 15:4;O(FA 15:4)	522.3219	[M-H]-	5.611	17861	14796	17723	15684	17752	14573	12000	20666	18936	13574	13627	14912	14949	17202	17208	18284
	NAGly	NAGly 28:2;O(FA 28:6)	886.73175	[M-H]-	9.412	5571	4421	386	5354	1088	1115	3587	1863	450	420	810	175	571	193	217	307
	NAGlySer	NAGlySer 10:0;O(FA 28:1)	735.58057	[M-H]-	6.822	40933	33801	29287	34442	31904	36106	37655	34259	37447	25503	32256	37281	31283	34703	30753	29994
	NAGlySer	NAGlySer 10:0;O(FA 28:6)	725.51154	[M-H]-	6.438	318	30	106	159	105	6745	180	95	132	119	129	169	584	184	175	3668
	NAGlySer	NAGlySer 14:0;O(FA 28:2)	789.63025	[M-H]-	6.905	2946	2582	3514	3595	3646	3470	3131	3577	4632	3606	4212	3805	2280	3869	3099	3582
	NAGlySer	NAGlySer 15:3;O(FA 28:6)	789.53668	[M-H]-	7.553	835	889	18	11	86	48	29	46	115	134	811	2305	1405	574	29	79
	NAGlySer	NAGlySer 16:1;O(FA 28:6)	807.58881	[M-H]-	7.918	11725	16058	9391	3226	4479	8925	6683	14101	10418	5543	3297	6742	6978	10242	13625	3590
	NAGlySer	NAGlySer 20:0;O(FA 28:1)	875.73511	[M-H]-	9.544	2642	2586	1381	3112	2569	1369	2231	2046	414	698	634	349	1152	844	945	848
	NAGlySer	NAGlySer 8:0;O(FA 24:1)	651.48828	[M-H]-	5.031	2753	2442	2665	2519	2561	2527	1944	2978	3832	2015	2762	2402	3421	3199	2439	2561
	NAGlySer	NAGlySer 8:0;O(FA 25:0)	667.51715	[M-H]-	7.137	14278	16409	15093	19940	17778	18479	23110	19415	17951	20423	21177	14992	18635	18737	19478	18581
	OxFA	FA 16:1;O	269.21121	[M-H]-	4.021	3739	3242	2962	3411	3258	2679	3283	2794	2721	1260	2814	2258	2230	2693	2758	2435
	OxFA	FA 18:1;O	297.24213	[M-H]-	4.193	5891	5706	4708	4263	6598	3768	4633	4067	3009	2421	2973	2866	4666	3137	2305	2220
	OxFA	FA 18:2;(2OH)	295.22668	[M-H]-	3.443	4174	2674	2403	2414	2146	1605	1812	1954	2494	811	1535	2310	2982	1911	1026	502
	OxFA	FA 20:0;O4	375.27371	[M-H]-	3.545	18033	18493	17002	17419	17950	17985	15175	20230	20519	14449	18685	17809	17225	20030	17621	18915
	OxFA	FA 22:0;O4	403.30579	[M-H]-	4.518	52658	49702	48961	48756	50086	48256	40634	51964	58104	40362	49150	46548	43351	52103	51994	50748
**Sphingolipids**	Cer	Cer 12:0;O3/21:0(2OH)	602.5119	[M-H]-	7.583	7143	10591	15280	11296	13879	8645	8256	7402	10917	8574	7928	11085	8882	7972	7572	6039
	Cer	Cer 12:0;O3/23:0(2OH)	584.52356	[M-H]-	7.297	2799	2887	3308	4033	3255	3270	2135	2260	1997	2089	1968	2103	3261	2102	2047	2780
	Cer	Cer 12:0;O3/30:0(2OH)	682.63019	[M-H]-	8.577	2779	2707	2398	2780	2675	3374	1991	2815	3004	2560	3145	3246	2984	3143	3263	2708
	Cer	Cer 12:0;O3/31:0(2OH)	696.64508	[M-H]-	8.722	3219	3416	3017	3474	3158	3986	3369	3238	3425	3376	2582	3773	3595	3863	3971	3581
	Cer	Cer 12:0;O3/33:0(2OH)	724.68286	[M-H]-	8.96	2127	2324	1101	2694	2861	2565	2460	2397	2910	2188	2819	2467	2678	2352	2920	2566

Cer	Cer 12:1;O3/22:0(2OH)	568.49274	[M-H]-	6.881	1812	1793	2046	2120	1892	1774	1460	1697	1196	1500	1180	1762	2642	1326	813	1065
Cer	Cer 12:1;O3/24:0(2OH)	596.52496	[M-H]-	7.371	5364	7640	10323	8309	8890	5874	6344	5366	8863	6729	6466	9750	8693	6565	4720	5172
Cer	Cer 12:1;O3/25:0(2OH)	656.55914	[M-H]-	8.536	2368	1158	504	1235	508	1006	1766	1453	227	486	573	367	556	651	523	537
Cer	Cer 12:1;O3/26:0(2OH)	624.55756	[M-H]-	7.782	10061	4971	4962	4562	5372	7585	4777	4384	5049	4795	6917	6561	5589	4627	4367	3759
Cer	Cer 12:1;O3/28:0(2OH)	652.58722	[M-H]-	8.154	4111	2253	1626	2031	1928	2900	2005	1908	2108	1765	2135	2478	2400	2248	1906	1638
Cer	Cer 12:1;O3/30:0(2OH)	726.63629	[M-H]-	6.51	75956	106417	76976	91511	103880	113808	104179	168009	122762	162551	134749	128085	111859	173572	163840	94109
Cer	Cer 12:1;O3/31:1(2OH)	692.61975	[M+FA-H]-	8.301	51625	42904	33271	36328	46997	49753	27391	42601	40574	29841	34578	53918	44367	41326	33556	24269
Cer	Cer 12:1;O3/32:0(2OH)	754.65912	[M-H]-	8.248	4117	3536	4266	3637	3831	3596	3341	3407	3944	3901	3833	3643	4079	3690	3502	3062
Cer	Cer 12:1;O3/32:3(2OH)	748.60748	[M-H]-	7.143	2277	3771	2708	4361	3915	3119	5156	2209	1335	4174	4004	4422	5291	3810	2523	4036
Cer	Cer 12:1;O3/32:5(2OH)	744.57239	[M-H]-	7.547	547	1130	663	342	566	2560	1424	620	2874	1898	958	1755	1421	1426	2194	2655
Cer	Cer 13:1;O3/26:3(2OH)	678.52594	[M-H]-	7.577	2412	2941	3786	3284	3676	2721	2437	2296	3050	2211	2429	3242	2767	2456	2238	1960
Cer	Cer 16:1;O3/38:6(2OH)	836.7135	[M-H]-	9.531	4180	1834	688	3074	1545	1574	4032	1971	345	555	162	180	361	697	730	412
Cer	Cer 16:2;O3/38:6(2OH)	834.69611	[M-H]-	9.366	4377	1998	862	3284	1286	1110	3892	2023	191	195	840	61	934	501	711	510
Cer	Cer 18:1;O3/38:6(2OH)	864.74402	[M-H]-	9.711	6945	3187	1430	5435	2606	2749	6663	3216	544	1116	1531	769	1661	1165	1264	1249
Cer	Cer 18:2;O3/38:6(2OH)	862.72937	[M-H]-	9.547	11780	5041	1780	8412	3723	3445	10164	5048	609	1229	292	864	1916	1183	1604	1353
Cer	Cer 20:1;O3/38:6(2OH)	892.77466	[M-H]-	9.878	3256	2334	614	3275	1782	1772	3137	1611	134	759	572	104	734	542	650	747
Cer	Cer 20:2;O3/38:6(2OH)	890.75952	[M-H]-	9.718	16362	10211	3864	12489	5691	6011	13371	7204	1107	2127	3480	1096	3366	2053	2219	2925
Cer	Cer 26:1;O3/38:5(2OH)	1024.89062	[M-H]-	6.994	27539	32858	34072	27905	33668	31217	28580	32255	33900	31689	31116	27457	29331	30106	29682	29674

Cer	Cer 37:2;O3|Cer 16:1;O2/21:1;O	576.55109	[M+H-H2O]+	6.028	51665	37029	490	13205	43606	54067	7213	46751	48495	48209	42511	50255	47998	51087	8928	3027
Cer	Cer 42:1;O3|Cer 18:1;O2/24:0;O	648.62622	[M+H-H2O]+	6.543	83637	80451	58483	61450	101802	97566	44859	98956	88094	60581	70320	119261	92537	84378	70249	47972
Cer	Cer 47:3;O3|Cer 29:3;O2/18:0;O	714.66815	[M+H-H2O]+	8.756	11652	8210	3029	11568	9060	12004	11140	11903	20629	20347	16422	21541	8388	13855	18028	16534
Cer	Cer 36:0;O2|Cer 18:0;O2/18:0	612.5553	[M+HCOO]-	7.728	2338	3132	3428	3555	3534	4084	2087	2506	3605	2503	4144	3089	3180	2891	3362	2370
Cer	Cer 36:1;O2|Cer 17:0;O2/19:1	588.53076	[M+Na]+	5.438	130866	224382	505670	238201	373449	231168	175254	217436	284903	238582	259169	329613	276381	222738	234646	191259
Cer	Cer 42:1;O2|Cer 17:0;O2/25:1	672.6203	[M+Na]+	7.045	20292	14454	2331	15222	18308	25264	12463	21133	19056	21096	15603	24660	21685	21538	22711	16619
Cer	Cer 48:0;O2|Cer 22:0;O2/26:0	736.74365	[M+H]+	8.292	11295	12141	314	7127	4239	9367	8673	13708	14468	12920	10545	14692	14715	14876	16322	9321
Cer	Cer 56:5;O2|Cer 18:0;O2/38:5	860.79736	[M+Na]+	9.147	40358	82605	138626	76810	80265	18311	38637	52911	20642	32051	31509	13476	27291	31651	5011	27130
Cer	Cer 56:6;O2|Cer 18:0;O2/38:6	858.78229	[M+Na]+	8.938	47601	83637	62604	83382	77527	21327	48234	56911	28177	36273	31569	17975	36642	32731	10874	29637
Cer	Cer 13:0;O2/26:5	598.51691	[M-H]-	7.374	2850	2807	2829	3595	2888	2911	2826	2780	2906	3628	2629	3140	2076	2876	2622	2921
Cer	Cer 13:0;O2/28:5	626.54645	[M-H]-	7.797	4443	4846	5056	6157	5837	5815	4904	5180	5406	5485	5407	6433	5394	5250	5344	4689
Cer	Cer 13:0;O2/30:5	654.58179	[M-H]-	8.161	3092	2410	2944	3009	2859	2300	1698	3317	2832	2764	3661	3212	2452	2958	2372	2152
Cer	Cer 13:0;O2/34:5	710.64209	[M-H]-	7.943	7640	7831	7738	6903	8815	7523	8429	7345	8302	8134	7672	8468	8779	6605	8139	8785
Cer	Cer 34:1;O2|Cer 16:1;O2/18:0	520.50452	[M+H-H2O]+	4.808	58623	65790	68598	71324	61712	58676	29769	33253	41376	36233	36320	44444	73902	38531	37816	31627
Cer	Cer 34:1;O2|Cer 18:1;O2/16:0	538.51288	[M+H]+	4.831	21620	26489	30809	33367	25011	26153	10893	22744	17466	18097	14475	14395	36350	18365	14780	10706
Cer	Cer 35:1;O2|Cer 17:1;O2/18:0	534.51947	[M+H-H2O]+	5.14	15894	24748	36412	27242	30764	12957	19744	22083	30788	25833	21992	31239	28329	20694	16242	20259
Cer	Cer 36:1;O2|Cer 18:1;O2/18:0	548.53894	[M+H-H2O]+	5.436	343466	588896	650436	533066	868223	505358	360401	421220	698565	460201	490351	758664	551062	441216	395320	292182
Cer	Cer 37:1;O2|Cer 17:1;O2/20:0	562.55188	[M+H-H2O]+	5.738	25964	14831	12360	12527	18232	22547	11971	14009	15721	13387	18561	22053	19309	14109	13689	10832
Cer	Cer 38:1;O2|Cer 18:1;O2/20:0	594.57764	[M+H]+	6.002	44900	12028	201	13606	11026	33379	10838	14203	14883	12043	19537	19065	18113	12901	12367	7644
Cer	Cer 40:1;O2|Cer 17:1;O2/23:0	644.58936	[M+Na]+	6.563	28235	19823	22539	16146	23998	25472	14331	26795	19503	17433	23314	26425	29406	26990	25239	20463
Cer	Cer 40:1;O2|Cer 18:1;O2/22:0	604.59918	[M+H-H2O]+	6.551	60454	42444	480	32964	46521	51386	29985	45215	38157	29178	40248	47138	51127	43581	40970	27605
Cer	Cer 40:1;O2|Cer 24:1;O2/16:0	666.60437	[M+HCOO]-	8.316	20793	13478	8458	11627	12863	15951	11476	12482	11528	8976	12108	13859	14943	12419	11441	8806
Cer	Cer 40:2;O2|Cer 26:1;O2/14:1	642.58185	[M+Na]+	5.89	6965	36533	3908	10172	45297	26038	30789	22364	22520	24831	25157	18825	10001	9170	15486	3846
Cer	Cer 41:1;O2|Cer 17:1;O2/24:0	618.61456	[M+H-H2O]+	6.803	53812	50711	5298	28883	45463	59949	30626	61978	39509	34512	45760	62249	57193	55768	61836	31897
Cer	Cer 41:1;O2|Cer 18:1;O2/23:0	680.62012	[M+HCOO]-	8.476	18465	14602	7473	10142	13060	16307	10075	14183	11733	9318	12814	16535	14875	15392	16583	9569
Cer	Cer 41:2;O2|Cer 17:1;O2/24:1	616.59747	[M+H-H2O]+	6.298	8621	8554	7749	7543	8166	9040	5031	9604	8435	7122	7630	11443	10482	9508	6544	6324
Cer	Cer 42:1;O2|Cer 18:1;O2/24:0	632.62915	[M+H-H2O]+	7.043	144444	110758	83536	87693	136292	166774	86144	137067	133313	103364	121417	150831	164063	151541	143745	94783
Cer	Cer 42:2;O2|Cer 18:1;O2/24:1	630.61554	[M+H-H2O]+	6.543	176059	152060	108499	117314	197066	208588	81573	178863	160646	113989	137549	230216	192275	161045	137597	86843

Cer	Cer 42:2;O2|Cer 26:2;O2/16:0	646.61102	[M-H]-	8.303	2001	1706	1374	1666	1984	1971	1307	1905	1658	1401	1546	2083	1885	1720	1405	1159
Cer	Cer 42:3;O2|Cer 18:1;O2/24:2	646.60645	[M+H]+	6.107	17054	19510	26806	18387	22431	21930	11672	21106	17533	17984	16001	23600	29596	21158	17570	17860
Cer	Cer 44:4;O2|Cer 20:1;O2/24:3	694.6048	[M+Na]+	8.548	7159	5252	25707	4088	5644	30740	5114	7514	18961	23779	10511	48059	18122	24274	15223	17820
Cer	Cer 44:4;O2|Cer 28:2;O2/16:2	694.60815	[M+Na]+	8.228	20226	21595	1718	19379	18684	22511	13424	25695	31356	14644	24709	26484	28448	32003	42995	32447
Cer	Cer 44:6;O2|Cer 20:1;O2/24:5	690.57605	[M+Na]+	7.797	37099	41511	38613	33391	38772	35829	26562	34837	49278	42990	46196	49336	42725	46935	45591	41754
Cer	Cer 45:2;O2|Cer 18:1;O2/27:1	712.67065	[M+Na]+	6.826	887	5150	1182	2019	5241	1762	819	5399	3317	3896	8699	3285	6835	5896	6124	4999
Cer	Cer 45:2;O2|Cer 20:1;O2/25:1	712.67377	[M+Na]+	7.025	4080	1935	1182	1052	2160	1762	756	4464	4491	2176	2555	2260	2092	2556	1563	1664
Cer	Cer 47:4;O2|Cer 29:3;O2/18:1	736.67102	[M+Na]+	7.037	791	859	307	0	506	1251	686	2362	700	468	909	1003	2445	786	654	927
Cer	Cer 48:1;O2|Cer 22:1;O2/26:0	716.72101	[M+H-H2O]+	8.222	2379	3893	5162	5520	5550	8046	6473	6956	5905	5223	3686	3210	9916	2463	3783	5538
Cer	Cer 51:1;O2|Cer 15:1;O2/36:0	798.75873	[M+Na]+	8.732	2752	3839	2390	3246	4580	1352	2259	2732	1643	2393	1785	5239	1588	3570	1842	751
Cer	Cer 52:6;O2|Cer 18:2;O2/34:4	802.69147	[M+Na]+	8.585	3393	1466	25355	5354	3860	8597	4689	7531	12462	5261	12151	13188	12778	11856	9932	7965
Cer	Cer 15:3;O2/32:6	702.57843	[M-H]-	7.598	12086	13021	17323	17897	16562	14666	12645	14433	17268	16835	17244	17068	17009	13836	12689	17080
Cer	Cer 15:3;O2/34:6	730.61066	[M-H]-	7.949	72456	81919	83206	73541	78654	76712	78319	76940	80102	85175	89268	84041	86364	86633	81102	87378
Cer	Cer 15:3;O2/36:6	758.6402	[M-H]-	8.258	3153	4087	3544	3006	4135	3702	3560	3413	3783	3401	3991	3922	3315	3928	3683	3844
Hex2Cer	Hex2Cer 36:1;O2|Hex2Cer 17:0;O2/19:1	890.64319	[M+H]+	4.469	7562	665	19280	1773	2623	2733	1847	7967	1625	7107	1182	4026	1551	2661	2512	3048
Hex2Cer	Hex2Cer 31:6;O2	854.48938	[M-H]-	7.135	4604	4829	4150	4562	4880	4246	5990	4435	5064	3530	4298	4630	5491	3717	5228	5476
HexCer	HexCer 16:1;O3/25:0(2OH)	874.66486	[M-H]-	8.358	1672	3817	1780	3731	3307	6690	1858	11228	6649	1788	6281	8478	5144	7430	4340	2624
HexCer	HexCer 16:1;O3/27:0(2OH)	856.68738	[M-H]-	8.21	444	1177	165	1711	811	299	530	3220	334	757	1831	224	47	1568	730	1124
HexCer	HexCer 17:0;O3/38:0(2OH)	1072.89441	[M-H]-	7.268	3302	3610	193	3518	1108	1900	2289	547	2628	1626	265	301	309	968	198	370
HexCer	HexCer 16:0;O2/17:1;O	746.54144	[M-H]-	6.651	108	46	19	107	16	25	36	36	230	44	81	291	26	24	82	40
HexCer	HexCer 17:0;O2/20:5;O	794.54303	[M-H]-	7.494	14807	823	11993	10692	4858	13383	313	10186	16052	14288	8214	16604	8606	9883	7705	7948
HexCer	HexCer 17:0;O2/22:6;O	820.56281	[M-H]-	7.524	6913	9018	7505	3308	6014	9473	4213	10008	7072	3804	5285	5732	6063	5914	5785	5876
HexCer	HexCer 42:1;O3|HexCer 18:1;O2/24:0;O	872.67944	[M+HCOO]-	8.113	252	2146	192	1743	1063	64	532	3646	700	972	1464	470	147	1487	914	2003
HexCer	HexCer 16:1;O2/26:6;O	860.58752	[M-H]-	6.769	8472	6367	13673	6913	6468	7153	4583	8477	5816	8113	6577	6311	12960	7789	6344	6084
HexCer	HexCer 16:2;O2/15:1;O	668.48132	[M-H]-	5.032	1599	1749	2172	1798	1846	1638	1407	1898	2495	1439	1886	1714	1962	1966	1832	1639

HexCer	HexCer 16:3;O2/22:6;O	800.49768	[M-H]-	7.292	2011	1513	2643	2568	2166	2508	2670	2186	2474	2369	1966	2630	2029	1210	2541	2852
HexCer	HexCer 17:1;O2/20:5;O	792.52576	[M+FA-H]-	6.901	20763	27966	24543	24545	22979	21415	24245	22986	27761	23899	22447	24098	27411	22053	24215	25717
HexCer	HexCer 17:2;O2/22:6;O	816.5329	[M+FA-H]-	6.577	2807	2427	452	2009	1943	2734	2596	2002	3601	2037	993	2784	3141	2446	3081	2825
SHex	SG 27:0;O;Hex	595.42493	[M-H]-	4.024	4678	4930	5060	4462	5353	3836	4383	3916	4736	3304	3676	3780	4954	4707	3426	3285
SHex	SG 29:1;O;Hex	621.43597	[M-H]-	5.688	2277	2216	2882	1868	2127	2038	1945	3170	3782	1924	1825	2561	2688	2752	2340	2433
SL	SL 13:1;O/28:3;O	708.52563	[M-H]-	6.799	2137	2050	1820	2385	2160	2086	1822	2180	2092	1866	2053	1746	1652	2062	1989	1866
SM	SM 33:1;O2	689.55359	[M+H]+	3.828	15732	28754	37306	24039	13344	37461	22535	66958	33751	26001	33041	48590	76560	60405	26727	42942
SM	SM 34:0;O2	705.5863	[M+H]+	4.373	39276	50128	51457	37506	39986	97862	15391	86909	47064	44008	64061	73561	101074	97067	58831	62944
SM	SM 34:1;O2|SM 16:1;O2/18:0	703.57574	[M+H]+	4.121	320575	700984	735691	474671	419514	912930	428677	1893589	661580	439242	711426	1049578	1999753	1379253	511130	834045
SM	SM 35:1;O2	717.58844	[M+H]+	4.49	65203	144937	81220	89253	78215	67899	94347	201778	154430	109828	155584	157332	179749	170956	49854	101186
SM	SM 35:3;O2	729.55688	[M+H]+	4.449	59654	66672	1110	25280	54732	23370	54616	57364	30674	41890	141505	71592	96922	71328	31304	28862
SM	SM 35:7;O2	721.50043	[M+H]+	5.371	99990	123959	1932	132281	127554	130085	102369	136057	142053	125753	125767	135374	151136	134282	150288	154652
SM	SM 36:1;O2	775.59692	[M+HCOO]-	7.2	28365	119574	97934	78559	73893	65980	74174	191518	102555	74083	94393	138280	138665	126928	71029	86940
SM	SM 36:1;O2|SM 18:1;O2/18:0	731.60461	[M+H]+	4.82	718058	2909924	2861430	1813784	2080798	1987579	1632545	5607990	2914554	2023826	2862771	3877266	4462127	4058854	2142005	2509781
SM	SM 37:4;O2	755.56641	[M+H]+	4.461	17497	29433	823	49634	17264	22462	13720	39544	11737	25701	37563	31731	88451	25996	28498	87392
SM	SM 37:5;O2|SM 19:0;O2/18:5	737.57068	[M+H]+	4.977	7620	18640	734	13766	26825	26909	13549	62537	51960	29937	13205	15404	21633	23508	7958	29707
SM	SM 37:8;O2	747.51025	[M+H]+	4.562	42396	34832	340	43459	31485	39341	28930	36488	33175	37253	10919	32797	26132	11621	30698	33982
SM	SM 37:9;O2	745.50372	[M+H]+	5.377	926	1109	3602	7594	5860	7849	3318	10033	8229	6787	7525	5631	3383	5054	11135	12394
SM	SM 38:0;O2	761.64996	[M+H]+	6.923	6709	48456	1533	46300	6870	2107	7582	33689	3440	0	6733	3923	12823	2709	2809	2371
SM	SM 38:1;O2	759.62988	[M+H]+	5.571	208893	122273	152821	116528	7955	390712	142870	477820	218746	180390	295937	303131	361956	408040	93129	226334
SM	SM 38:7;O2	747.53076	[M+H]+	5.278	885	3134	1177	1262	1547	1802	4056	3452	707	15119	2821	30736	2433	1958	15297	592
SM	SM 38:9;O2	759.51697	[M+H]+	5.4	28604	12226	1436	12400	1245	10650	1317	876	3767	4557	1168	0	0	847	584	810
SM	SM 39:8;O2	759.53497	[M+H]+	6.246	1020	0	39677	0	1245	0	0	0	0	887	0	0	3102	1658	0	0
SM	SM 39:9;O2	773.53156	[M+H]+	5.392	39717	46334	48575	54278	48734	45394	31886	47789	47624	39721	48494	50469	45367	46192	42421	47909
SM	SM 40:1;O2	787.66156	[M+H]+	6.334	6204	68712	19421	34567	41416	87441	51864	301608	104542	52765	85412	154910	177859	171631	82434	104047
SM	SM 40:9;O2	787.52734	[M+H]+	4.813	3906	2658	1171	739	2291	1176	594	630	1474	1478	1469	1477	5112	3837	886	348
SM	SM 41:1;O2	801.6803	[M+H]+	6.68	49432	75869	2886	48290	53311	92077	54940	198701	71762	54711	92021	119163	126305	157153	98193	82916
SM	SM 41:2;O2|SM 22:0;O2/19:2	799.6626	[M+H]+	5.929	3124	50114	30739	31252	957	48330	30591	115331	54262	38308	68782	81376	85882	91321	46388	53143
SM	SM 41:7;O2	789.60449	[M+H]+	5.169	616494	290374	2656	365064	433416	608894	360637	574525	549669	359852	660827	533590	678853	701882	509269	438322

	SM	SM 41:8;O2	787.58899	[M+H]+	4.546	163732	104984	181395	30576	80643	122699	116746	33850	192154	86618	128179	198428	51141	258062	229346	184380
	SM	SM 41:9;O2	785.57434	[M+H]+	4.709	43721	158618	30111	95838	63659	53801	136515	46325	51026	119645	73230	125535	83026	149068	59622	271627
	SM	SM 42:1;O2	815.68787	[M+H]+	6.412	11119	22318	10598	5796	29644	52255	3439	53860	36935	23808	14050	42441	20528	42847	42994	22933
	SM	SM 42:2;O2|SM 18:1;O2/24:1	813.68488	[M+H]+	6.263	379994	714410	375187	447225	541830	1030654	359452	2091674	807509	582072	846057	1173871	1362062	1552510	663550	723296
	SM	SM 42:3;O2	833.64038	[M+Na]+	5.651	3504	5698	679	4995	7130	18267	7363	17771	15624	3773	18568	24181	36291	27307	10383	8128
	SM	SM 42:3;O2|SM 20:0;O2/22:3	811.66229	[M+H]+	5.668	71772	165825	182183	125220	112463	252216	104171	402352	185340	138072	171502	283738	495940	334037	165364	191703
	SM	SM 43:5;O2	821.65485	[M+H]+	4.926	4158	226	89	221	159	2648	1918	2347	1179	1180	3162	1713	1257	1475	2656	2952
	SM	SM 12:1;O2/29:0	845.67267	[M-H]-	8.739	2375	2012	953	2027	1231	2239	639	7957	2052	1651	1318	2053	3248	3112	988	1776
	SM	SM 12:1;O2/30:1	857.67242	[M-H]-	8.356	19704	25825	18114	10393	21996	36718	20746	61293	32618	22235	14090	42917	44013	52920	26170	26459
	SM	SM 12:1;O2/35:0	929.77985	[M-H]-	9.725	3192	4003	1962	3426	3052	1503	1977	2213	451	831	1122	398	1491	659	716	1041
	SM	SM 37:6;O3	795.52936	[M-H]-	6.877	1754	2460	5308	5147	4854	2446	2111	5091	7005	2237	4855	3544	2442	3468	5195	2392
	SM	SM 39:4;O3	827.59741	[M-H]-	7.95	4430	5263	4646	5664	4837	5373	5305	4624	5097	6161	4703	5324	3431	5699	5241	5340
	SM	SM 42:2;O3	873.67603	[M-H]-	8.207	412	1064	435	1597	779	548	144	3065	602	670	1746	527	508	1450	773	1301
	SM	SM 42:3;O3	871.65778	[M-H]-	7.857	158	1946	279	2097	878	262	347	4304	734	732	2202	427	188	1802	534	1407
	SM	SM 43:0;O3	891.72247	[M-H]-	9.562	3764	3402	2897	2009	3344	2260	3252	2936	971	1478	1372	731	2706	1620	1181	1816
	SM	SM 45:0;O3	919.76038	[M-H]-	8.814	1609	2483	266	4304	552	1335	1218	735	40	86	338	6	1004	51	444	105
	SM	SM 45:1;O3	917.73749	[M-H]-	9.593	2677	3059	2252	2750	2260	1020	1787	2029	592	1098	1239	178	1891	916	523	1121
**Glycerolipids**	DG	DG 23:3	459.30017	[M+Na]+	2.802	60176	71881	58222	63514	74319	70230	53648	74619	60081	49676	79230	59540	73296	69991	61493	61684
	DG	DG 30:0	563.46191	[M+Na]+	5.293	32407	55548	101938	70046	75261	47725	54264	54954	34267	35394	33071	27863	55491	33514	42289	42389
	DG	DG 30:1	561.44495	[M+Na]+	4.731	18636	26125	20421	32580	39829	11704	35790	18335	4143	7382	6318	4727	19735	9378	8853	5679
	DG	DG 30:1|DG 12:0_18:1	556.4873	[M+NH4]+	4.726	42775	56886	1007	53211	79971	24777	60622	31615	19870	11628	15743	8058	61078	13259	19237	15980
	DG	DG 31:4	569.42462	[M+Na]+	6.062	11479	7864	18915	12011	11719	13316	7510	16268	12275	12094	13923	16982	16785	15306	17864	19182
	DG	DG 32:0	591.49451	[M+Na]+	5.842	1330090	1546002	10298	1467876	1695583	1549517	1229941	1752722	1620718	1605120	1678614	1780213	1711303	1840579	1980299	1951635
	DG	DG 32:0|DG 16:0_16:0	586.53949	[M+NH4]+	5.872	616201	726384	768703	676244	960832	710149	521468	779941	761406	566512	718687	774733	840264	766490	682739	583888
	DG	DG 32:1	589.47748	[M+Na]+	5.35	114313	158084	5349	216124	306306	97032	228104	158802	49359	66911	66416	44423	153267	59555	74977	63778
	DG	DG 32:1|DG 16:0_16:1	584.52319	[M+NH4]+	5.356	237331	338048	253029	327766	610691	192394	402561	231387	104801	89558	132815	90264	303628	108085	103243	85269
	DG	DG 32:2	587.45557	[M+Na]+	4.822	10672	26230	591	26967	19285	16230	30799	6305	3239	14606	3796	1847	20135	1883	9564	1650
	DG	DG 32:2|DG 16:1_16:1	582.50568	[M+NH4]+	4.832	87369	120229	886	110426	162492	52547	111912	78719	50065	33751	46762	33550	131138	50395	50996	37149
	DG	DG 33:0	605.50336	[M+Na]+	6.195	4820	19135	1093	20848	16568	3375	22765	23057	24410	30611	28034	26535	28144	25744	21912	30294
	DG	DG 33:1	603.49298	[M+Na]+	5.665	2960	16312	28804	13061	22939	12490	8331	17368	11335	9900	14129	11239	21585	15637	13849	9818

DG	DG 33:1|DG 15:0_18:1	598.53558	[M+NH4]+	5.615	18566	20907	19615	17613	25396	17622	18168	17007	16752	10789	19507	16669	27456	16486	13038	4025
DG	DG 33:2|DG 15:0_18:2	596.52222	[M+NH4]+	5.174	5563	15515	16138	7490	6707	11646	10682	8070	12358	5884	8304	3768	19380	7205	1365	12447
DG	DG 34:0	619.52576	[M+Na]+	6.378	2241146	2457674	45973	2277902	2573238	2464598	1996808	2584184	2554996	2486756	2823838	2905832	2754527	2815927	2853040	2971394
DG	DG 34:0|DG 16:0_18:0	614.56952	[M+NH4]+	6.401	1367723	1634897	65513	1345206	1571854	1553044	1046513	1613669	2018102	1434616	1692374	1775699	1544972	1856914	1603630	1367110
DG	DG 34:1	617.51044	[M+Na]+	5.889	747184	1107020	60692	1005324	1810542	906262	1079392	798000	693429	594613	759412	830550	1417305	795572	642220	486616
DG	DG 34:1|DG 16:0_18:1	612.55634	[M+NH4]+	5.911	1027063	1866158	73971	1451174	3366266	1151091	1513650	1219825	959076	641278	1065751	985908	2011542	1033156	600528	407446
DG	DG 34:2	615.49255	[M+Na]+	5.449	391812	841949	11758	677240	1016963	425715	668988	586014	406244	371131	486028	448584	1036327	522860	389511	406768
DG	DG 34:2|DG 16:0_18:2	610.53888	[M+NH4]+	5.474	669250	1419583	1158558	927762	1756152	625646	1000695	735819	607372	425768	670008	589943	1630644	692544	374009	364062
DG	DG 34:3	613.4743	[M+Na]+	5.022	10014	14856	81279	21240	19819	3177	13249	6539	8004	7718	7320	2315	14403	11011	10860	3134
DG	DG 34:3|DG 16:1_18:2	608.5202	[M+NH4]+	4.936	92837	114310	886	86333	82655	27137	81229	40458	48685	31409	30588	30871	108550	29115	15444	15333
DG	DG 34:4|DG 16:2_18:2	606.50299	[M+NH4]+	4.647	8907	15344	16373	16453	18911	2423	16074	4227	5983	4285	248	10763	12934	5141	4269	1454
DG	DG 35:0	633.53381	[M+Na]+	7.072	1877	628	14806	315	4393	421	853	2476	735	2922	433	16252	895	723	913	1204
DG	DG 35:2|DG 17:1_18:1	624.54846	[M+NH4]+	5.477	1514	4352	651	2265	29276	3819	1516	2308	4269	2573	4701	2713	3532	4725	1685	877
DG	DG 35:3	627.48523	[M+Na]+	5.238	1908	7545	278	1612	4901	591	6842	662	367	475	523	571	2939	271	1372	259
DG	DG 36:0	647.55432	[M+Na]+	6.355	1629	1644	4303	552	1467	1161	599	24402	15646	2641	18257	1841	20305	2223	621	6313
DG	DG 36:0|DG 18:0_18:0	642.60059	[M+NH4]+	6.892	689439	793830	381112	647913	789593	733401	531683	795862	935716	673289	824779	923107	731939	915326	737014	747640
DG	DG 36:1	645.54022	[M+Na]+	6.466	65271	118367	173519	108619	179818	85616	79494	136675	153656	92364	159728	123645	174838	125660	87602	78062
DG	DG 36:1|DG 18:0_18:1	640.58411	[M+NH4]+	6.442	102003	177218	282632	156386	238735	136905	124484	184995	243917	125446	209664	179930	229623	190422	115535	98139
DG	DG 36:2	643.52661	[M+Na]+	6.021	387493	918667	1068816	409901	1258860	412154	768781	640160	521997	230837	701174	303600	817571	571648	241883	340254
DG	DG 36:2|DG 18:1_18:1	638.57025	[M+NH4]+	6.012	525438	1372265	875125	487761	1912743	508193	1105177	519688	454603	270364	625688	297134	855172	452009	216432	192237
DG	DG 36:3	641.51129	[M+Na]+	5.543	210994	482870	841567	289968	550074	143846	285956	228241	194557	178917	239561	231090	652509	231097	124921	147141
DG	DG 36:3|DG 18:1_18:2	636.55505	[M+NH4]+	5.523	329213	771258	7170	351226	794569	178259	401881	250424	255403	195566	276632	265109	850709	275058	116533	129359
DG	DG 36:4	639.49188	[M+Na]+	5.077	27873	58063	155263	60188	51750	21468	46194	25726	27869	30834	26109	22507	67123	26756	20764	27639
DG	DG 36:4|DG 16:0_20:4	634.53595	[M+NH4]+	5.361	17932	28079	224176	19753	22147	23666	16361	27609	23341	15559	23596	24722	33172	23122	18294	2814
DG	DG 36:4|DG 18:2_18:2	634.53534	[M+NH4]+	5.047	130392	240678	4549	157485	179795	83894	134298	97423	106601	80311	119680	115024	306969	123890	67197	81527
DG	DG 36:5	637.47668	[M+Na]+	4.642	8415	2288	14257	17954	11598	8746	12416	4728	1708	4619	2209	1626	14794	8657	1495	9060
DG	DG 37:0|DG 16:0_21:0	656.61053	[M+NH4]+	8.173	56438	49645	40979	18228	38504	54131	48325	56221	55589	64358	32993	40333	46915	32074	80680	38397

DG	DG 37:1|DG 16:0_21:1	654.59338	[M+NH4]+	6	8253	9223	14101	9512	15552	535	8209	7802	3543	3687	6997	7678	14122	3474	2522	2392
DG	DG 37:7	647.45679	[M+Na]+	6.861	12809772	14729562	14126655	11329502	15398570	14172616	10718988	14166106	15781611	13303203	14593541	17602526	14712951	16005603	14284448	14436369
DG	DG 38:0	675.58148	[M+Na]+	7.398	8301	7306	11696	568	8388	8498	7054	9741	8489	9222	8921	13138	9073	11352	9433	8966
DG	DG 38:0|DG 18:0_20:0	670.62921	[M+NH4]+	6.907	149249	194840	204228	164205	192813	197846	142519	182666	241863	198324	200564	234584	187618	231864	191323	194362
DG	DG 38:2	671.56555	[M+Na]+	6.477	3247	7415	6792	18920	22942	1619	9120	3680	18005	8956	14453	8714	24250	22789	2952	3573
DG	DG 38:2|DG 18:1_20:1	666.6004	[M+NH4]+	5.997	135144	260006	31803	114779	341757	157788	216931	115736	129694	71746	192391	72987	163903	182540	61632	27418
DG	DG 38:3	669.55151	[M+Na]+	6.175	8100	19243	30977	29123	21668	12381	1348	40611	40763	15731	34807	22454	47570	32558	18535	12693
DG	DG 38:3|DG 18:0_20:3	664.58258	[M+NH4]+	6.157	15810	47809	75131	49607	34879	17352	11613	70372	46128	29663	60160	30180	67082	49708	25058	33722
DG	DG 38:4	667.52704	[M+Na]+	5.955	10047	108201	328840	127498	65798	31930	17219	361068	206195	167888	467077	163132	245505	262219	61743	73618
DG	DG 38:4|DG 18:0_20:4	662.5708	[M+NH4]+	5.959	31685	332460	43083	263016	197710	70100	45300	625746	338886	238194	701005	245220	440400	396654	95318	103218
DG	DG 38:8	659.46954	[M+Na]+	5.734	1477	454	232	273	878	263	255	419	813	228	756	1272	915	1470	830	260
DG	DG 40:4	695.55469	[M+Na]+	6.347	2733	8754	19655	13033	10588	1882	3561	23377	18036	13787	25826	14563	19320	20906	8943	8047
DG	DG 40:4|DG 18:0_22:4	690.59943	[M+NH4]+	5.96	11192	45351	51063	42431	28718	17199	11258	77764	56084	43301	89576	42993	60537	59959	19673	21008
DG	DG 40:5	693.53912	[M+Na]+	6.26	1063	2683	2067	21368	892	3411	657	26687	24542	1096	26479	23995	29104	27905	1528	991
DG	DG 40:5|DG 18:0_22:5	688.58411	[M+NH4]+	5.963	1059	7231	5891	25378	13507	1596	0	30400	25858	12998	30818	22495	34442	30765	5483	3439
DG	DG 42:4	723.59015	[M+Na]+	7.209	110724	115339	70552	123419	70118	108487	65295	128016	111713	122214	127734	127351	120735	133305	148967	130248
DG	DG 43:1	743.66412	[M+Na]+	7.568	23343	16526	23466	11213	25084	19112	21480	24169	13264	13131	16181	20127	6582	20038	16197	12844
DG	DG 43:6	733.57062	[M+Na]+	7.38	1869	3427	1609	21696	2445	3580	6934	27754	5093	5285	2471	3188	2623	3257	5604	1854
DG	DG 43:7	731.5567	[M+Na]+	7.127	22993	17007	904	25848	20394	15275	18879	20742	1659	1780	4644	2275	15662	2134	12132	836
DG	DG 44:6	747.59998	[M+Na]+	6.106	43626	33050	855774	17074	22896	37157	25230	19204	15286	23382	18521	36429	27425	19805	28046	16841
DG	DG 45:10	753.55701	[M+Na]+	4.714	140613	133951	11236	121581	242135	208408	90786	67070	207383	110078	240870	168949	230993	110673	286625	252443
DG	DG 45:12	749.50684	[M+Na]+	3.036	11552	15077	539	11359	14464	11779	8275	11790	12146	12164	13104	14945	10351	13900	14451	902
DG	DG 45:6	761.59766	[M+Na]+	7.856	29183	25025	2509	24588	42480	32805	32077	42793	35399	19191	15056	22646	29114	25345	15865	40491
DG	DG 45:7	759.59369	[M+Na]+	7.645	2258	1433	1707	1731	42727	5524	22844	43242	805	1942	2177	1457	8472	1031	2167	0
DG	DG 45:8	757.57074	[M+Na]+	7.193	33874	39026	516	35842	33519	18673	29322	44353	3303	8341	17104	5780	27959	9684	16597	12479
DG	DG 46:1|DG 15:0_31:1	780.75781	[M+NH4]+	8.354	2230	4165	4106	3544	3619	3237	3430	5950	2397	4665	4067	5394	4182	7476	1186	5849
DG	DG 46:11	765.5528	[M+Na]+	4.769	242023	293353	7286	288393	95588	10551	265415	281260	214831	174236	214649	301591	271993	197317	257255	283821

DG	DG 46:8	771.59521	[M+Na]+	4.428	53166	42868	2638	34309	58346	11071	9729	16288	20973	19902	43386	29183	30459	27442	2774	11458
DG	DG 46:9	769.58881	[M+Na]+	6.446	15080	10052	8011	7822	16050	5850	9232	5917	11368	4997	24314	18364	10291	12859	4125	3111
DG	DG 47:0	801.74585	[M+Na]+	8.341	1177	898	900	1181	1505	2026	893	1191	1198	1194	596	1597	1788	1492	598	2766
DG	DG 47:1|DG 14:0_33:1	794.76147	[M+NH4]+	8.761	1494	889	1696	5350	1197	2333	4264	1188	1500	2623	2506	2434	993	1510	2704	1401
DG	DG 47:6	789.63184	[M+Na]+	8.189	33583	28826	30679	38200	40209	49950	39378	49328	39945	43474	44949	51436	36951	52072	52180	43801
DG	DG 47:7	787.61664	[M+Na]+	7.89	91862	92136	72081	103235	119739	86812	87897	121627	52100	67526	81605	49164	100662	78080	84978	67797
DG	DG 47:8	785.60022	[M+Na]+	7.591	84731	111631	74445	108919	112005	52626	82324	125428	23581	32466	58514	23470	87132	45381	50259	46398
DG	DG 47:9	783.58551	[M+Na]+	7.227	23696	35900	896	35003	30568	3123	22510	38491	4647	4845	16448	4410	25973	7295	13513	13786
DG	DG 48:0	815.75891	[M+Na]+	8.934	199	2392	945	1069	1508	1794	1988	348	2479	1669	1144	1694	2967	600	1607	599
DG	DG 48:1	813.75128	[M+Na]+	8.92	4100	2692	30920	1143	1075	3495	4929	2451	839	6807	1967	3151	1983	4868	4332	3610
DG	DG 48:13	789.54791	[M+Na]+	4.555	8756	13937	14255	54057	77127	7557	21154	3897	6726	44387	95001	61289	10307	124845	5175	5632
DG	DG 48:6	803.64728	[M+Na]+	8.538	1701	7856	1928	1830	1013	6296	1038	930	9994	1190	733	1190	436	892	3870	972
DG	DG 48:9	797.61749	[M+Na]+	5.654	183540	9683	102892	86229	379747	136164	88544	226408	104761	182715	248916	149100	111701	143802	9324	111248
DG	DG 49:0	829.77686	[M+Na]+	8.836	2113	1272	3206	2100	8862	2389	2621	2398	925	2415	5321	2412	7443	4488	2924	3909
DG	DG 49:10	809.60059	[M+Na]+	7.098	1664	21786	435	19437	16888	1765	1340	21577	627	1038	334	872	1432	598	553	644
DG	DG 49:12	805.57239	[M+Na]+	5.218	8817	6259	14185	6830	7775	2749	13039	10339	12484	3287	18230	8099	18038	10633	12046	4845
DG	DG 49:6	817.66302	[M+Na]+	8.49	30904	24465	24248	31711	39137	43162	33881	35905	51476	42693	32747	61625	40925	60768	37949	37488
DG	DG 49:7	815.65076	[M+Na]+	8.21	97795	86045	89783	115223	122523	100828	113342	138350	67315	92702	91984	84992	104551	103295	106199	90007
DG	DG 49:8	813.63196	[M+Na]+	7.92	157773	149979	123845	162306	175716	106078	145688	195773	60423	78755	112740	61571	137089	92773	106809	96375
DG	DG 49:9	811.61438	[M+Na]+	7.623	68296	90219	66522	86066	79500	30248	56499	104088	18331	24510	39347	19048	68587	31407	25438	33976
DG	DG 50:0	843.76489	[M+Na]+	9.151	1044	596	1545	787	6565	1790	3887	3277	1202	1069	4411	1368	7594	6977	3688	1302
DG	DG 50:1	841.78174	[M+Na]+	8.931	29218	7802	3085	30458	40964	30792	30536	32061	3449	17371	26435	32140	4583	20236	18537	29224
DG	DG 50:10	823.63324	[M+Na]+	5.812	5174	20546	38588	3611	7738	7400	6413	28867	9720	8307	6732	5306	57617	4613	56944	6934
DG	DG 50:11	821.61523	[M+Na]+	5.448	51086	24138	16559	9625	28185	42172	18194	18222	34206	33332	40364	37067	47258	4241	23169	7756
DG	DG 50:14	815.56903	[M+Na]+	4.745	28266	5798	14115	9262	30155	22905	22025	10407	34010	8722	6262	41512	23165	47070	11337	10283
DG	DG 50:2	839.74554	[M+Na]+	8.558	19612	28165	3734	38581	26308	15566	27441	33222	9568	16097	17442	7926	32858	20058	12286	21503
DG	DG 50:2|DG 16:1_34:1	834.77966	[M+NH4]+	8.998	68206	96173	118847	102570	120949	29114	50175	54106	50990	44852	30372	53267	50802	22887	2152	17916
DG	DG 50:3	837.72742	[M+Na]+	8.315	18568	25798	1745	25317	18375	2308	14368	22108	2871	5505	10211	1878	23882	4791	5811	7365

DG	DG 50:3|DG 18:1_32:2	832.76562	[M+NH4]+	8.921	29931	49692	40806	51334	52417	15361	35980	45173	13810	26347	24243	12182	25211	22841	10448	22471
DG	DG 50:4	835.71655	[M+Na]+	7.514	1413	1298	14390	46201	30199	1552	13717	33646	7052	10530	11332	3704	24703	9449	2483	1610
DG	DG 50:6	831.67621	[M+Na]+	8.65	13670	6694	892	15008	9014	11097	7997	17440	24560	19389	15639	20716	13174	7038	10932	17806
DG	DG 51:10	837.63147	[M+Na]+	7.681	50828	85866	65573	81159	69999	22236	40848	92421	15137	19353	31538	16083	72206	27508	22371	28692
DG	DG 51:2	853.7699	[M+Na]+	8.294	2194	6254	2442	990	2198	4260	1484	4245	1604	5030	3259	1697	899	3491	6508	1681
DG	DG 51:3	851.75165	[M+Na]+	8.024	1388	1381	4714	1887	1300	2082	1286	1682	1746	1003	1090	1513	6851	7651	993	3553
DG	DG 51:4	849.73871	[M+Na]+	7.689	1490	949	3564	1043	11988	5493	6079	10573	2275	1237	3211	2691	12892	1132	934	7317
DG	DG 51:6	845.69141	[M+Na]+	8.76	30963	23878	1823	36473	39772	31057	41094	38342	50231	27704	37115	57708	35829	61495	30156	28368
DG	DG 51:7	843.6778	[M+Na]+	8.503	120768	99501	97910	124936	128546	117202	118265	133493	99363	112634	114618	105628	127113	129261	141664	113568
DG	DG 51:8	841.66132	[M+Na]+	8.238	231578	205453	188842	240855	249669	208673	244237	242648	137174	170359	212855	151349	229919	185676	216544	205339
DG	DG 51:9	839.64807	[M+Na]+	7.959	185638	207627	164681	221255	213983	100219	174411	229421	65778	87411	132217	66694	180015	109285	112218	116044
DG	DG 52:1	869.78491	[M+Na]+	9.321	289	14097	3440	13681	28833	6818	2317	2608	1358	15121	2368	3725	805	1142	920	5145
DG	DG 52:6	859.7074	[M+Na]+	8.936	18671	3810	4727	7408	15572	9375	14039	6752	13535	15949	13281	19708	17051	17420	11649	9046
DG	DG 52:7	857.703	[M+Na]+	8.568	6334	14536	1804	10800	8699	12205	5794	13286	3527	0	5132	3639	2737	7279	7792	5226
EtherDG	DG O-27:1|DG O-13:0_14:1	500.46112	[M+NH4]+	3.963	9535	10319	11533	8355	7699	7417	10194	12430	8266	7755	8168	10411	11376	9711	12825	9921
EtherDG	DG O-29:1|DG O-15:0_14:1	528.49109	[M+NH4]+	4.609	15631	16171	13523	17916	10883	8241	10449	28299	16302	9745	19981	7634	17216	14687	17284	10519
EtherDG	DG O-31:1|DG O-17:0_14:1	556.52618	[M+NH4]+	5.236	358697	400941	74963	370058	406542	357687	276374	364696	414207	413811	347079	405504	342585	394661	448326	450613
EtherDG	DG O-35:1|DG O-17:0_18:1	612.58508	[M+NH4]+	6.741	365	1495	49570	700	455	465	298	1269	489	249	311	200	652	1878	660	300
EtherDG	DG O-35:4|DG O-19:3_16:1	606.54626	[M+NH4]+	5.081	2723	8624	1598	2391	578	13888	4394	865	4791	1935	10680	1222	1735	7095	2232	13126
EtherDG	DG O-36:2|DG O-20:2_16:0	624.59729	[M+NH4]+	5.438	11917	14803	25562	15144	19171	19129	12242	8833	19292	17293	15612	20397	16854	14392	14263	14436
EtherDG	DG O-37:1|DG O-23:0_14:1	640.6192	[M+NH4]+	6.951	16521	24072	32447	17621	32931	22809	19131	27901	30088	24405	35166	24618	27512	27550	29738	27254
EtherDG	DG O-37:3|DG O-19:2_18:1	636.60114	[M+NH4]+	5.564	1005	1277	1475	1024	8241	1709	746	815	930	1309	805	575	548	1118	1291	836
EtherDG	DG O-45:1|DG O-17:1_28:0	752.7583	[M+NH4]+	8.107	4653	3297	2288	2973	1270	506	750	1443	499	1550	851	831	2095	898	1552	869
MG	MG 18:0	376.34006	[M+NH4]+	2.25	67731	75969	77111	67218	76762	76616	48179	82669	87495	63189	79740	73699	72496	89202	82682	67555
MG	MG 29:0	530.52344	[M+NH4]+	5.453	7317	13693	87	14315	22842	15305	11487	14397	17843	9292	12062	16836	15659	12061	12453	9427
MG	MG 31:1	556.52283	[M+NH4]+	4.52	74872	77556	1018	47818	69077	70101	53381	71841	75884	75237	63608	85100	61308	76779	63032	58819
MG	MG 33:1	584.55646	[M+NH4]+	5.245	48695	49897	585	49903	47011	53295	35589	51955	53167	51533	51038	56208	46363	56872	53899	66494

MG	MG 35:0	614.61328	[M+NH4]+	6.962	5468	1757	965	2988	479	1502	243	1661	2154	1306	422	1550	3259	509	901	3622
MG	MG 35:1	612.58838	[M+NH4]+	6.417	42216	48601	1468	43858	51474	47321	35177	54508	52965	45738	51393	52897	55757	58073	52437	49470
MG	MG 36:2	624.5979	[M+NH4]+	6.021	356	14917	610	89	553	977	12242	1510	530	1331	3479	5800	637	14293	428	333
OxTG	TG 46:2;O3|TG 14:0_14:0_18:2;O3	840.68512	[M+NH4]+	7.395	1339	1902	5307	854	4027	28834	1550	4942	25027	12229	9848	38322	1386	28524	18984	36242
OxTG	TG 48:1;O|TG 16:0_16:0_16:1;O	838.74255	[M+NH4]+	6.938	9357	7801	701	11728	11844	7253	7258	10043	15861	4881	4845	3615	17624	4358	6483	30667
OxTG	TG 48:1;O|TG 18:0_18:1_12:0;O	838.74097	[M+NH4]+	7.055	24282	53475	2294	75724	56966	23072	22472	29791	7618	10222	14766	3944	31167	12609	38474	18565
OxTG	TG 48:1;O3|TG 16:0_16:0_16:1;O3	870.73694	[M+NH4]+	7.854	961137	1266324	1069174	1416183	1038465	748670	823401	1070188	740833	718979	790530	668810	1037972	742219	687143	825104
OxTG	TG 48:2;O|TG 15:0_15:0_18:2;O	836.72827	[M+NH4]+	7.833	2435	8370	12521	85466	67313	19956	29887	49172	2218	25323	33496	7845	46701	21642	11585	2384
OxTG	TG 48:2;O|TG 16:0_17:0_15:2;O	836.72656	[M+NH4]+	7.059	68565	93064	2932	98044	129939	69226	65838	66582	17795	16204	44802	11505	46133	38791	31100	28081
OxTG	TG 48:3;O|TG 18:1_17:2_13:0;O	834.71136	[M+NH4]+	7.577	24058	44207	1036	76810	54040	15827	21373	50204	9719	17875	23914	11746	45731	16272	16443	7528
OxTG	TG 49:3;O|TG 17:1_17:1_15:1;O	848.73175	[M+NH4]+	6.897	5931	8366	1805	13306	8947	1903	3794	11412	1519	4051	3033	4560	22101	12891	5602	3698
OxTG	TG 49:4;O|TG 16:0_16:1_17:3;O	846.70001	[M+NH4]+	8.728	10819	4518	1565	5347	25709	11561	16698	2871	7242	5748	14624	14507	8542	14583	5374	767
OxTG	TG 49:4;O|TG 16:1_16:1_17:2;O	846.73206	[M+NH4]+	7.498	16071	40695	788	1059	47381	10949	13378	1230	3290	1928	11638	4312	34831	3482	6046	531
OxTG	TG 49:4;O2|TG 16:2_18:2_15:0;O2	862.72064	[M+NH4]+	8.165	2744	1898	10610	1686	0	1190	891	891	623	1737	1492	743	2631	595	993	1782
OxTG	TG 49:6;O|TG 14:1_18:2_17:3;O	842.70117	[M+NH4]+	7.516	475581	451302	476473	478272	439696	465163	475908	472060	527632	455800	449377	491134	447774	458077	440077	453152
OxTG	TG 50:1;O|TG 16:0_18:1_16:0;O	866.77277	[M+NH4]+	7.346	111537	102285	892	184782	105506	133747	78438	83132	5716	4117	44762	5202	96404	29828	31216	47417
OxTG	TG 50:2;O|TG 16:0_16:1_18:1;O	864.75983	[M+NH4]+	7.535	153479	203341	100150	573074	389597	113665	131535	249321	4489	42851	90961	6061	179340	87696	79387	66231
OxTG	TG 50:2;O|TG 16:0_18:1_16:1;O	864.75934	[M+NH4]+	7.225	137393	204621	42705	226892	233864	169919	142766	159224	10260	22180	66716	6678	135083	33146	86372	74521
OxTG	TG 50:2;O|TG 16:1_16:1_18:0;O	864.75879	[M+NH4]+	7.029	102011	105371	297	182144	158385	74011	97064	110695	13072	21497	60152	12274	71760	29495	62011	30726

OxTG	TG 50:3;O|TG 16:0_16:1_18:2;O	862.74646	[M+NH4]+	7.156	106767	321106	56684	468979	176290	50686	73843	217676	10727	35761	52774	8909	236597	70663	59466	37231
OxTG	TG 50:4;O|TG 15:0_17:1_18:3;O	860.72766	[M+NH4]+	6.919	26103	86299	2021	80512	65834	18273	14339	45698	11577	9198	27369	4285	81945	18845	9490	28535
OxTG	TG 50:4;O|TG 17:0_18:2_15:2;O	860.73175	[M+NH4]+	6.598	7034	905	211	24504	18692	7481	15484	20192	1404	7993	5691	366	18386	606	9395	7695
OxTG	TG 50:4;O|TG 17:1_18:2_15:1;O	860.72864	[M+NH4]+	7.324	1102	2046	2713	2602	1467	585	1176	23648	463	7945	418	1251	773	542	970	6995
OxTG	TG 50:5;O|TG 15:0_18:2_17:3;O	858.73322	[M+NH4]+	8.1	4849	4995	9795	9684	22927	4528	3601	45014	3137	6430	14890	6622	9123	22643	10580	6121
OxTG	TG 51:0;O|TG 17:0_17:0_17:0;O	882.82367	[M+NH4]+	9.053	131256	180446	2916	177273	188242	140631	128631	153879	67731	87249	104238	56845	128436	113220	88502	119571
OxTG	TG 51:5;O|TG 16:0_18:2_17:3;O	872.72174	[M+NH4]+	8.733	26138	72739	2200	34390	23636	31893	34650	87493	49711	2598	89623	66792	64965	37696	60201	56325
OxTG	TG 51:5;O|TG 18:2_18:2_15:1;O	872.74371	[M+NH4]+	7.652	8779	3645	0	44370	1915	13379	87516	8341	3427	3883	49188	6088	37258	5162	4838	101979
OxTG	TG 52:2;O|TG 16:0_16:0_20:2;O	892.79205	[M+NH4]+	7.091	18633	53113	403	456003	23695	35638	6372	44340	3473	1734	4725	1248	32074	2755	6962	23336
OxTG	TG 52:2;O|TG 19:0_18:1_15:1;O	892.79102	[M+NH4]+	7.789	211585	419383	118165	469926	571759	60542	170310	349107	26610	63248	154998	24710	243338	113317	157286	104089
OxTG	TG 52:3;O|TG 16:0_18:1_18:2;O	890.77704	[M+NH4]+	7.328	608942	1096766	2489	511432	927697	777194	553102	675717	41107	71291	240441	13788	503656	110562	367214	313502
OxTG	TG 52:3;O|TG 16:0_19:1_17:2;O	890.77966	[M+NH4]+	7.513	430112	1065179	6455	1466424	882089	373147	344879	661562	52831	71877	269005	29301	725977	118431	231737	239622
OxTG	TG 52:4;O|TG 16:0_18:2_18:2;O	888.76215	[M+NH4]+	7.021	196915	418759	409	341327	269170	132476	150142	249965	14460	28254	81730	6907	156431	38704	97200	111419
OxTG	TG 52:4;O|TG 17:0_18:2_17:2;O	888.76178	[M+NH4]+	7.253	100011	385931	262429	892524	181726	50639	66204	194038	13861	26110	57133	7300	332449	44618	25684	57305
OxTG	TG 52:5;O|TG 17:1_18:2_17:2;O	886.74323	[M+NH4]+	6.945	18972	52078	57827	93240	27193	9339	9892	38019	10586	9387	14583	4781	60191	16221	7183	21000
OxTG	TG 52:5;O|TG 18:2_18:2_16:1;O	886.74261	[M+NH4]+	6.61	12021	29172	304	1699	28398	7093	6580	16447	1307	1486	4195	888	21598	1270	7131	11791
OxTG	TG 52:6;O|TG 18:1_17:3_17:2;O	884.74744	[M+NH4]+	7.733	1008	69851	919	50885	187745	267470	172913	234964	316349	327284	285188	311312	4813	31205	222594	24589
TG	TG 24:0|TG 8:0_8:0_8:0	488.38705	[M+NH4]+	8.643	23020	22457	21838	6390	19544	9121	20478	11497	14453	14814	9056	13536	16095	18475	22225	19675
TG	TG 36:0|TG 12:0_12:0_12:0	656.58551	[M+NH4]+	6.359	32921	15531	1341	31652	19773	37229	9897	85480	19191	31707	35039	23145	20939	16123	33986	32966

<table>
<tr><td>TG</td><td>TG 38:2|TG 10:0_10:0_18:2</td><td>680.57507</td><td>[M+NH4]+</td><td>6.428</td><td>47032</td><td>37275</td><td>25462</td><td>50528</td><td>36919</td><td>43836</td><td>41986</td><td>31452</td><td>47877</td><td>37630</td><td>56612</td><td>29998</td><td>51809</td><td>27285</td><td>58285</td><td>42437</td></tr>
<tr><td>TG</td><td>TG 39:0|TG 9:0_13:0_17:0</td><td>698.6214</td><td>[M+NH4]+</td><td>6.899</td><td>19553</td><td>24886</td><td>3873</td><td>22588</td><td>27425</td><td>19961</td><td>24487</td><td>32387</td><td>15748</td><td>25442</td><td>30788</td><td>22909</td><td>24507</td><td>29252</td><td>19764</td><td>28773</td></tr>
<tr><td>TG</td><td>TG 40:0|TG 10:0_14:0_16:0</td><td>712.6438</td><td>[M+NH4]+</td><td>7.496</td><td>404832</td><td>504048</td><td>4477</td><td>619236</td><td>608146</td><td>357903</td><td>456871</td><td>695457</td><td>238815</td><td>259169</td><td>311818</td><td>219968</td><td>462291</td><td>339608</td><td>288246</td><td>294347</td></tr>
<tr><td>TG</td><td>TG 40:0|TG 12:0_12:0_16:0</td><td>712.63824</td><td>[M+NH4]+</td><td>8.394</td><td>16301</td><td>16364</td><td>342954</td><td>5218</td><td>8845</td><td>5682</td><td>4610</td><td>13637</td><td>6649</td><td>15453</td><td>9251</td><td>19463</td><td>12052</td><td>16646</td><td>11801</td><td>8446</td></tr>
<tr><td>TG</td><td>TG 40:0|TG 12:0_14:0_14:0</td><td>717.59943</td><td>[M+Na]+</td><td>7.482</td><td>75680</td><td>70869</td><td>25966</td><td>78632</td><td>84076</td><td>58991</td><td>79255</td><td>90130</td><td>48941</td><td>53337</td><td>78285</td><td>83172</td><td>79230</td><td>76931</td><td>88110</td><td>87357</td></tr>
<tr><td>TG</td><td>TG 40:0|TG 8:0_14:0_18:0</td><td>712.63556</td><td>[M+NH4]+</td><td>9.001</td><td>8164</td><td>8760</td><td>10208</td><td>19813</td><td>4747</td><td>11501</td><td>5021</td><td>14854</td><td>13818</td><td>9296</td><td>5009</td><td>7391</td><td>3240</td><td>10803</td><td>8197</td><td>7946</td></tr>
<tr><td>TG</td><td>TG 40:0|TG 9:0_9:0_22:0</td><td>712.63489</td><td>[M+NH4]+</td><td>6.898</td><td>2132</td><td>12548</td><td>7762</td><td>7959</td><td>4510</td><td>14625</td><td>11217</td><td>7899</td><td>9221</td><td>4053</td><td>4168</td><td>14564</td><td>5263</td><td>13622</td><td>8444</td><td>6703</td></tr>
<tr><td>TG</td><td>TG 40:1|TG 12:0_12:0_16:1</td><td>715.5824</td><td>[M+Na]+</td><td>7.13</td><td>67530</td><td>68848</td><td>8004</td><td>72188</td><td>70749</td><td>42196</td><td>64982</td><td>68773</td><td>14084</td><td>15792</td><td>31912</td><td>11246</td><td>53821</td><td>28237</td><td>44981</td><td>7322</td></tr>
<tr><td>TG</td><td>TG 40:1|TG 8:0_14:0_18:1</td><td>710.62714</td><td>[M+NH4]+</td><td>7.14</td><td>482778</td><td>426642</td><td>154863</td><td>561259</td><td>416287</td><td>285167</td><td>379873</td><td>440889</td><td>85700</td><td>92836</td><td>172897</td><td>77507</td><td>305397</td><td>141173</td><td>233988</td><td>161482</td></tr>
<tr><td>TG</td><td>TG 40:2|TG 8:0_16:1_16:1</td><td>708.60828</td><td>[M+NH4]+</td><td>6.805</td><td>163057</td><td>159373</td><td>74173</td><td>181774</td><td>74517</td><td>57402</td><td>86819</td><td>118078</td><td>12849</td><td>41159</td><td>45359</td><td>13681</td><td>69405</td><td>7517</td><td>41565</td><td>9894</td></tr>
<tr><td>TG</td><td>TG 41:0|TG 12:0_13:0_16:0</td><td>726.65564</td><td>[M+NH4]+</td><td>7.663</td><td>156521</td><td>63211</td><td>143168</td><td>76995</td><td>107450</td><td>91030</td><td>152385</td><td>83812</td><td>86032</td><td>153261</td><td>155425</td><td>159478</td><td>93125</td><td>157640</td><td>153626</td><td>154758</td></tr>
<tr><td>TG</td><td>TG 42:0|TG 12:0_14:0_16:0</td><td>740.67456</td><td>[M+NH4]+</td><td>7.859</td><td>1116725</td><td>1308784</td><td>37905</td><td>1603442</td><td>1717512</td><td>1125474</td><td>1312810</td><td>1931227</td><td>701562</td><td>760530</td><td>955078</td><td>699179</td><td>1260708</td><td>1140404</td><td>886696</td><td>893683</td></tr>
<tr><td>TG</td><td>TG 42:0|TG 14:0_14:0_14:0</td><td>745.62689</td><td>[M+Na]+</td><td>7.865</td><td>70319</td><td>52560</td><td>91346</td><td>67485</td><td>96844</td><td>95050</td><td>92636</td><td>96407</td><td>79149</td><td>85878</td><td>95166</td><td>92705</td><td>92425</td><td>114607</td><td>102016</td><td>98227</td></tr>
<tr><td>TG</td><td>TG 42:1|TG 10:0_16:0_16:1</td><td>738.6593</td><td>[M+NH4]+</td><td>7.538</td><td>2063363</td><td>2246678</td><td>929740</td><td>2721968</td><td>2311568</td><td>1175290</td><td>1790696</td><td>2458970</td><td>296323</td><td>411820</td><td>771700</td><td>257574</td><td>1394859</td><td>575367</td><td>826101</td><td>619490</td></tr>
<tr><td>TG</td><td>TG 42:1|TG 12:0_12:0_18:1</td><td>743.61664</td><td>[M+Na]+</td><td>7.533</td><td>144819</td><td>165276</td><td>114240</td><td>163250</td><td>179794</td><td>96848</td><td>141616</td><td>204528</td><td>2952</td><td>29467</td><td>75077</td><td>2621</td><td>130773</td><td>56341</td><td>88714</td><td>67737</td></tr>
<tr><td>TG</td><td>TG 42:2|TG 10:0_16:1_16:1</td><td>741.59827</td><td>[M+Na]+</td><td>7.195</td><td>93273</td><td>113739</td><td>69854</td><td>115178</td><td>95250</td><td>51522</td><td>82227</td><td>134141</td><td>4770</td><td>18611</td><td>43650</td><td>11057</td><td>81828</td><td>29183</td><td>47568</td><td>34987</td></tr>
<tr><td>TG</td><td>TG 42:2|TG 8:0_16:1_18:1</td><td>736.6438</td><td>[M+NH4]+</td><td>7.208</td><td>770238</td><td>1007625</td><td>312019</td><td>1081838</td><td>712612</td><td>344952</td><td>625110</td><td>960463</td><td>80166</td><td>108224</td><td>233182</td><td>58834</td><td>521583</td><td>150028</td><td>244595</td><td>189964</td></tr>
<tr><td>TG</td><td>TG 42:3|TG 8:0_10:0_24:3</td><td>734.62421</td><td>[M+NH4]+</td><td>6.873</td><td>79718</td><td>113100</td><td>40713</td><td>114712</td><td>68262</td><td>48372</td><td>57696</td><td>95382</td><td>12910</td><td>20537</td><td>36006</td><td>4545</td><td>70782</td><td>20533</td><td>31594</td><td>28532</td></tr>
<tr><td>TG</td><td>TG 43:0|TG 13:0_14:0_16:0</td><td>754.68787</td><td>[M+NH4]+</td><td>8.006</td><td>486506</td><td>440925</td><td>451547</td><td>445961</td><td>441916</td><td>573720</td><td>518612</td><td>501741</td><td>607051</td><td>566289</td><td>521312</td><td>531613</td><td>442596</td><td>558227</td><td>585120</td><td>530519</td></tr>
<tr><td>TG</td><td>TG 43:1|TG 13:0_13:0_17:1</td><td>752.66791</td><td>[M+NH4]+</td><td>8.067</td><td>15222</td><td>6815</td><td>234110</td><td>14556</td><td>19288</td><td>4612</td><td>8955</td><td>21428</td><td>8361</td><td>10760</td><td>15083</td><td>12870</td><td>22295</td><td>6940</td><td>7532</td><td>16105</td></tr>
<tr><td>TG</td><td>TG 43:1|TG 14:0_15:0_14:1</td><td>752.67242</td><td>[M+NH4]+</td><td>7.713</td><td>247794</td><td>301278</td><td>3376</td><td>311257</td><td>297499</td><td>256852</td><td>241027</td><td>276801</td><td>197364</td><td>211626</td><td>231794</td><td>199632</td><td>232858</td><td>236251</td><td>223911</td><td>212089</td></tr>
<tr><td>TG</td><td>TG 43:2|TG 9:0_16:0_18:2</td><td>750.65649</td><td>[M+NH4]+</td><td>7.403</td><td>63839</td><td>109068</td><td>72020</td><td>116314</td><td>89766</td><td>51920</td><td>74330</td><td>84925</td><td>39092</td><td>54827</td><td>51386</td><td>48714</td><td>75604</td><td>66824</td><td>57581</td><td>46423</td></tr>
<tr><td>TG</td><td>TG 44:0|TG 14:0_14:0_16:0</td><td>768.70514</td><td>[M+NH4]+</td><td>8.182</td><td>3061956</td><td>3562542</td><td>96749</td><td>4168564</td><td>5005359</td><td>2985107</td><td>3473976</td><td>5549296</td><td>2147010</td><td>2232034</td><td>2673454</td><td>2554708</td><td>3628181</td><td>3644690</td><td>2353478</td><td>2537376</td></tr>
<tr><td>TG</td><td>TG 44:1|TG 10:0_16:0_18:1</td><td>766.6911</td><td>[M+NH4]+</td><td>7.89</td><td>6415454</td><td>7581680</td><td>4058650</td><td>9089954</td><td>8473755</td><td>4247192</td><td>5852486</td><td>8960455</td><td>1522216</td><td>2291311</td><td>3339102</td><td>1504977</td><td>5194932</td><td>2952499</td><td>3124081</td><td>2906952</td></tr>
<tr><td>TG</td><td>TG 44:1|TG 13:0_13:0_18:1</td><td>771.64386</td><td>[M+Na]+</td><td>7.887</td><td>273644</td><td>276523</td><td>248321</td><td>279659</td><td>346285</td><td>265544</td><td>300716</td><td>367712</td><td>146293</td><td>198072</td><td>244417</td><td>157903</td><td>274550</td><td>246222</td><td>260609</td><td>234082</td></tr>
<tr><td>TG</td><td>TG 44:2|TG 10:0_16:0_18:2</td><td>764.6745</td><td>[M+NH4]+</td><td>7.589</td><td>4274624</td><td>5552469</td><td>2285090</td><td>5952326</td><td>4884596</td><td>1863861</td><td>3445228</td><td>5596812</td><td>525122</td><td>766792</td><td>1665613</td><td>484261</td><td>3204354</td><td>1206034</td><td>1299756</td><td>1241796</td></tr>
<tr><td>TG</td><td>TG 44:2|TG 10:0_16:1_18:1</td><td>764.66949</td><td>[M+NH4]+</td><td>6.934</td><td>3669</td><td>679</td><td>277</td><td>1480</td><td>1908</td><td>4656</td><td>3896</td><td>464</td><td>898</td><td>626</td><td>63203</td><td>2007</td><td>4232</td><td>29759</td><td>54238</td><td>1207</td></tr>
</table>

TG	TG 44:2|TG 16:0_14:1_14:1	769.62836	[M+Na]+	7.59	291985	352367	308021	348282	364309	159068	268992	418820	62172	103595	177094	64613	286668	146677	150419	151959
TG	TG 44:3|TG 10:0_16:1_18:2	762.65771	[M+NH4]+	7.273	586093	965554	356243	947618	656919	214979	442438	857428	74752	92223	192685	75340	502354	101499	141890	161879
TG	TG 44:3|TG 8:0_18:1_18:2	767.61267	[M+Na]+	7.264	59873	102840	68548	97383	80131	24847	51490	111230	3537	13197	28126	9893	70148	21582	23950	25255
TG	TG 44:4|TG 8:0_18:2_18:2	760.6463	[M+NH4]+	6.878	8017	63555	8292	125827	70617	0	0	44009	0	0	0	0	58027	0	0	0
TG	TG 45:0|TG 13:0_15:0_17:0	782.71399	[M+NH4]+	8.692	58608	69513	699553	92828	59487	146401	70921	72969	88709	103178	70188	80697	59465	82349	84290	66608
TG	TG 45:0|TG 14:0_15:0_16:0	782.72034	[M+NH4]+	8.355	706524	592823	3081	589776	726444	1043852	884527	720088	742684	1049059	923356	1226551	450505	1075677	1118325	979581
TG	TG 45:0|TG 15:0_15:0_15:0	782.7149	[M+NH4]+	9.031	45322	54825	12300	22551	68577	21580	57547	68863	26246	24960	61212	25485	23196	23746	52305	74562
TG	TG 45:1|TG 14:0_14:0_17:1	780.69672	[M+NH4]+	8.812	5454	7301	44116	3921	18225	8509	24862	3291	19710	9641	8699	9741	16523	31804	38346	8433
TG	TG 45:1|TG 14:0_15:0_16:1	780.70441	[M+NH4]+	8.054	585913	580755	544388	552992	652636	683084	636803	642178	626201	598154	579497	607199	400045	625212	619771	556347
TG	TG 45:2|TG 11:0_16:1_18:1	778.68652	[M+NH4]+	7.765	323621	350495	277384	346487	285467	263692	298105	380191	211443	215945	261218	214934	301336	240324	234997	244552
TG	TG 45:2|TG 13:0_16:1_16:1	783.64154	[M+Na]+	7.763	29342	26981	32415	31483	35426	30054	24403	38767	29247	29384	30095	29829	33163	26565	33178	30788
TG	TG 45:3|TG 11:0_18:1_16:2	776.67114	[M+NH4]+	7.427	31338	48322	1450	46325	39230	20613	24696	48081	4962	11744	18477	9251	7022	16655	15901	12895
TG	TG 45:3|TG 14:1_14:1_17:1	776.67145	[M+NH4]+	7.538	33911	30975	47577	31809	45554	18020	24471	38326	13254	19890	24380	13760	39238	24620	6164	8328
TG	TG 46:0|TG 14:0_14:0_18:0	801.68927	[M+Na]+	7.959	84282	99888	62147	115690	66536	9959	36325	61041	1247	9937	31919	5313	46164	33337	11644	31351
TG	TG 46:0|TG 14:0_16:0_16:0	796.73627	[M+NH4]+	8.486	6212866	5945794	5698410	7725410	9899977	6476900	7254222	8267996	5204896	4783751	5402924	5481098	7136621	8486062	4775761	5488769
TG	TG 46:1|TG 14:0_14:0_18:1	799.67456	[M+Na]+	7.261	3003	36788	2169	1721	3422	4384	3103	5492	2626	12856	2843	3062	30828	3630	13787	2613
TG	TG 46:1|TG 14:0_16:0_16:1	794.72235	[M+NH4]+	8.204	21726564	20503406	105782	27231456	28449534	12596717	19610574	27373812	5559012	7220444	11089024	5821958	16197499	10367885	10013494	9068324
TG	TG 46:2|TG 14:0_16:1_16:1	797.66156	[M+Na]+	7.917	517859	486326	470180	556024	561372	351616	487600	643874	216275	278632	386700	227085	476571	338778	404278	342149
TG	TG 46:2|TG 16:0_14:1_16:1	792.70667	[M+NH4]+	7.922	14588665	15307211	8568308	18999474	15937509	5959992	11633638	17938644	2489363	3461337	5973896	2339334	10180619	4318652	5038884	4529476
TG	TG 46:2|TG 16:0_15:1_15:1	792.70551	[M+NH4]+	9.118	15705	28853	12997	14072	11554	38338	23108	26581	14575	9970	39996	27093	20073	21892	10558	22839
TG	TG 46:3|TG 14:1_14:1_18:1	795.64551	[M+Na]+	7.628	218740	307795	256749	278938	254205	84371	188087	351908	47732	68285	127354	41208	229125	84415	89450	91926
TG	TG 46:3|TG 14:1_16:1_16:1	790.68958	[M+NH4]+	7.623	2598793	3953291	1678381	3894727	2907333	716778	1907959	3789844	303777	411367	861537	287622	2028468	540546	530429	614736
TG	TG 46:4|TG 10:0_18:1_18:3	788.67426	[M+NH4]+	7.31	231273	530988	192950	494193	295631	75209	177140	467009	32512	38361	96830	32380	309188	64884	51499	74727
TG	TG 46:4|TG 14:1_14:1_18:2	793.63037	[M+Na]+	7.348	29730	54978	45579	52574	38926	10315	18981	55774	1423	8754	16447	3331	39910	3413	1713	15059
TG	TG 47:0|TG 15:0_15:0_17:0	815.70331	[M+Na]+	8.682	30858	18433	1648	31793	34543	44828	51421	39538	78626	66383	50319	82233	6469	66036	72311	62710
TG	TG 47:0|TG 15:0_16:0_16:0	810.75189	[M+NH4]+	8.658	913915	815421	52190	919052	1079547	1204473	1116726	1036616	1599539	1318484	1120666	1594154	1075143	1399185	1320341	1199626
TG	TG 47:1|TG 15:0_15:0_17:1	813.69104	[M+Na]+	8.41	41379	2840	3774	41286	46895	60281	53134	47303	64336	54031	54278	75954	39420	52637	61418	45288

TG	TG 47:1|TG 15:0_16:0_16:1	808.73572	[M+NH4]+	8.39	1101682	988414	809652	1080392	1243024	1199387	1222205	1264092	1006172	978409	1014845	1023273	959938	1042977	1061383	999826
TG	TG 47:1|TG 15:0_17:0_15:1	808.755	[M+NH4]+	8.085	0	747	832	1013	1502	0	0	4463	4454	0	0	0	0	0	0	0
TG	TG 47:2|TG 14:0_16:1_17:1	806.71826	[M+NH4]+	8.1	768203	835798	641447	830623	955248	650275	733658	923356	549199	577230	630606	545363	722642	604728	598928	322210
TG	TG 47:2|TG 15:0_16:1_16:1	811.67169	[M+Na]+	8.118	48079	12450	2677	26359	33856	28196	22732	45133	33541	24107	26703	37468	27564	37727	18489	14362
TG	TG 47:3|TG 14:1_16:1_17:1	804.70233	[M+NH4]+	7.816	185342	259007	151856	228326	159523	108244	140633	162512	102979	62844	62554	103940	183818	56672	96715	113309
TG	TG 48:0|TG 16:0_16:0_16:0	824.76965	[M+NH4]+	8.771	7934322	6409070	6824676	9194855	13964936	7430062	9589894	7963126	7438686	5329888	6713880	8216963	8209052	11034608	6016668	5035220
TG	TG 48:1|TG 14:0_14:0_20:1	827.70227	[M+Na]+	8.069	189802	270046	430247	299122	220868	69550	109671	221446	40983	53478	85306	31397	194127	70074	49847	72818
TG	TG 48:1|TG 14:0_16:0_18:1	822.75262	[M+NH4]+	8.505	49620064	45724612	10628	58246320	63489868	39626624	49182944	53854228	18204574	25637516	35300464	16329451	41731184	33638120	32677634	30847460
TG	TG 48:2|TG 14:0_16:1_18:1	820.73602	[M+NH4]+	8.244	70336232	68138584	45194580	81959840	75951408	33510110	58108516	75493328	13747421	19094670	34376748	12621315	47977844	24280426	27944278	25104020
TG	TG 48:2|TG 16:0_16:1_16:1	825.69318	[M+Na]+	8.224	973635	892462	32534	1090449	1118453	740239	961908	1125132	471114	561514	740928	506362	858607	656153	739430	655820
TG	TG 48:3|TG 14:0_16:1_18:2	818.72125	[M+NH4]+	7.961	18815086	23512882	13134621	25363152	19690994	5268630	12966302	21709620	2598814	3828032	7010739	2401253	13402560	4620148	4510688	5485903
TG	TG 48:3|TG 16:1_16:1_16:1	823.67358	[M+Na]+	7.959	657619	674193	617819	727944	725642	360769	579353	756539	223466	307830	435718	224268	593759	359766	376860	415246
TG	TG 48:4|TG 14:0_14:0_20:4	821.65912	[M+Na]+	7.681	162263	276960	256065	269843	214209	55446	123008	273647	33869	45155	87992	42019	227608	79636	51986	81368
TG	TG 48:4|TG 14:1_16:1_18:2	816.70648	[M+NH4]+	7.68	1796849	3590330	1763857	3694850	2382521	409527	1205978	2794820	235672	277091	588243	193851	2088035	472172	324485	493183
TG	TG 48:5|TG 12:0_12:0_24:5	819.63983	[M+Na]+	7.442	7789	27109	34345	34883	28679	11098	8709	17033	9890	12494	7428	11997	3749	2085	11678	17412
TG	TG 48:5|TG 12:0_18:2_18:3	814.68115	[M+NH4]+	7.474	69071	104797	105850	107058	62037	14677	52514	106044	11166	40349	71829	15727	64034	42714	10847	73992
TG	TG 48:5|TG 14:1_16:1_18:3	814.68726	[M+NH4]+	7.359	109241	195871	70494	244069	151602	51442	87680	205347	29370	23380	58657	18039	164834	67107	32574	62865
TG	TG 48:6|TG 14:0_14:1_20:5	812.68927	[M+NH4]+	6.912	3463	1666	888	29195	2919	9551	14459	16875	25970	6973	10221	15031	4670	14446	9428	11330
TG	TG 49:0|TG 15:0_16:0_18:0	838.80438	[M+NH4]+	9.461	0	0	18641	7780	34830	0	8052	0	45153	0	0	40026	0	0	0	0
TG	TG 49:0|TG 16:0_16:0_17:0	838.78247	[M+NH4]+	8.922	894604	996161	808270	990904	1315298	933713	1020238	979606	1142016	906988	958193	1095473	1021544	1217657	903937	893303
TG	TG 49:1|TG 15:0_16:0_18:1	836.76752	[M+NH4]+	8.673	3010465	3422033	2090618	3787065	4335392	2647066	3039865	3413660	1546299	1650640	2259368	1451515	2446939	2163187	1739879	1895659
TG	TG 49:1|TG 16:0_16:0_17:1	841.71674	[M+Na]+	8.675	84843	81815	63599	85888	101296	92494	95649	97684	70214	83003	89650	75819	81174	95377	84727	83124
TG	TG 49:2|TG 15:0_17:1_17:1	839.70721	[M+Na]+	8.415	102268	98459	73954	91519	109604	91403	107368	115527	65760	71401	90976	65365	91354	89291	89317	77032
TG	TG 49:2|TG 16:0_16:1_17:1	834.75018	[M+NH4]+	8.415	3171736	3679071	2006212	3596576	3805958	1925619	2833012	3561396	1096571	1262079	1886586	1047919	2522528	1608992	1407648	1550937
TG	TG 49:3|TG 16:1_16:1_17:1	832.7334	[M+NH4]+	8.126	739955	1134783	2087	1080712	976489	383222	629823	926369	259925	316243	462824	246346	762336	380708	246091	419147
TG	TG 49:4|TG 16:1_18:1_15:2	830.71661	[M+NH4]+	7.844	110520	170420	108724	152545	137192	38218	69537	142865	30886	37394	56368	22064	144134	47269	65298	50156
TG	TG 50:0|TG 16:0_16:0_18:0	852.79742	[M+NH4]+	9.047	8837683	9947078	6875827	11991377	15723116	8249982	9516434	8679914	6451688	4150860	6788964	6161598	7633019	9902254	5178685	4444080

TG	TG 50:1|TG 14:0_18:0_18:1	855.72552	[M+Na]+	8.262	14667	26738	3690	14002	33452	62291	4188	13600	3725	49846	36199	32404	10732	9298	59428	5599
TG	TG 50:1|TG 16:0_16:0_18:1	850.78485	[M+NH4]+	8.779	76819640	70105840	59317408	89386832	98874928	64267744	74553624	74828776	34107784	46256336	56180680	31079806	61307088	57463508	52058336	52504532
TG	TG 50:2|TG 14:0_18:1_18:1	853.72192	[M+Na]+	8.516	1423446	1225247	340282	1373521	1309222	1396395	1448886	1367564	779220	959315	1294510	748706	1264474	1055922	1358814	1151624
TG	TG 50:2|TG 16:0_16:0_18:2	848.75916	[M+NH4]+	8.969	179266	98910	144912	94788	89191	91240	73289	60712	124904	50232	101299	41977	82628	76077	60326	89362
TG	TG 50:2|TG 16:0_16:1_18:1	848.76685	[M+NH4]+	8.538	128809920	129502328	98677632	144888592	147075888	97932424	119725216	128800488	42513912	60720500	85004128	37254972	96222744	66143932	78944616	75090240
TG	TG 50:3|TG 16:0_16:0_18:3	851.70581	[M+Na]+	8.262	1303215	1240521	47294	1419547	1434870	774949	1072086	1367970	519813	630055	857934	533808	1008999	673906	708993	831689
TG	TG 50:3|TG 16:0_16:1_18:2	846.75336	[M+NH4]+	8.278	86834064	96287664	68524360	99368840	92480824	31866778	61170552	83871440	16296687	23042850	38971580	14427336	60615784	25301988	23615452	31557430
TG	TG 50:3|TG 16:1_16:1_18:1	851.70477	[M+Na]+	7.823	30169	73992	1060307	111035	48200	15959	17716	52384	6663	9258	16509	6530	51686	15806	8717	11094
TG	TG 50:4|TG 16:0_16:0_18:4	849.69049	[M+Na]+	8.007	610564	749568	686921	751304	663781	314765	481715	679170	227642	310158	431882	217120	656632	354391	315875	422584
TG	TG 50:4|TG 16:1_16:1_18:2	844.73639	[M+NH4]+	8.003	17722916	29214888	11421	29450912	19916490	4813951	10429140	20478680	2972722	4369378	7309924	2560499	17510338	5182096	3674438	6562660
TG	TG 50:5|TG 14:0_18:2_18:3	842.72089	[M+NH4]+	7.711	1742096	3944438	2337394	4631592	2421777	511051	1021206	2726995	334947	472879	741543	319680	2392554	702238	360796	848723
TG	TG 50:5|TG 16:1_16:1_18:3	847.67554	[M+Na]+	7.737	180706	318251	309877	301413	218772	86487	140004	262327	52324	86066	107955	58530	262262	112672	65849	135752
TG	TG 50:6|TG 14:0_18:3_18:3	840.70648	[M+NH4]+	7.48	72418	173638	122744	294046	66758	13911	40865	178155	13545	16316	24055	10992	148976	22712	13186	29656
TG	TG 51:0|TG 16:0_17:0_18:0	866.81274	[M+NH4]+	9.177	461362	561315	396637	562650	668662	426493	464959	463325	464882	371231	441867	400589	465388	508019	380316	392368
TG	TG 51:1|TG 16:0_17:0_18:1	864.79803	[M+NH4]+	8.937	3375180	5232403	2620268	4655514	5705662	2770794	2845188	3790653	1325660	1620173	2528121	1174508	2906356	2278580	1451363	2234145
TG	TG 51:2|TG 15:0_18:1_18:1	867.73511	[M+Na]+	8.688	252576	297451	204998	282466	270921	224620	237099	267720	120705	131427	209347	112161	221617	168101	172318	191090
TG	TG 51:2|TG 16:0_16:0_19:2	867.72534	[M+Na]+	7.229	1892	1485	1218	114380	0	3729	952	2929	1708	4169	1577	3053	888	2380	1804	1899
TG	TG 51:2|TG 16:0_17:1_18:1	862.78357	[M+NH4]+	8.685	10657934	16813090	8117816	14057948	14101958	7590186	8711014	11292797	2481240	3356373	6132578	2069611	7677303	4224289	3663336	5133904
TG	TG 51:3|TG 16:0_17:1_18:2	860.76831	[M+NH4]+	8.436	5115894	9061875	4385826	6736501	6460430	1981062	3635168	5573241	1127331	1452158	2814876	940217	5185600	1870963	1164424	2308378
TG	TG 51:3|TG 17:1_17:1_17:1	865.71918	[M+Na]+	8.439	164556	219878	159332	192766	185299	96750	133930	174412	70277	81682	141591	71329	181530	102705	75320	113661
TG	TG 51:4|TG 15:0_18:2_18:2	858.75189	[M+NH4]+	8.174	737839	1837854	939989	1393489	1094176	271702	494876	929704	209858	273957	482095	166506	1329728	372026	168624	425708
TG	TG 51:5|TG 15:0_18:2_18:3	856.7301	[M+NH4]+	7.908	72466	267974	9306	266483	180811	88057	95709	184890	76215	81975	127618	42624	256969	108797	56192	117902
TG	TG 52:0|TG 16:0_18:0_18:0	880.82892	[M+NH4]+	9.293	3987042	6270236	3577952	5900882	7873582	4541880	3939822	4333912	3329017	2398930	3396263	3101693	4090274	4544941	2573708	2623009
TG	TG 52:1|TG 12:0_22:0_18:1	883.76788	[M+Na]+	9.044	806042	958020	317288	1013861	1186064	880691	814612	977714	475027	679052	808899	499290	797929	771067	756701	806718
TG	TG 52:1|TG 16:0_18:0_18:1	878.81372	[M+NH4]+	9.06	52710792	69525160	42730312	73164288	79019864	56201884	44555400	55286228	19364258	30518964	40290792	16283781	40637960	37645248	30279326	38944012
TG	TG 52:2|TG 16:0_18:1_18:1	876.80048	[M+NH4]+	8.806	193551952	240465760	165691776	230687248	235337936	171473664	168655808	198041264	69787832	101150560	142439360	57932448	155468784	104143496	122465632	137190768

	TG	TG 52:3|TG 16:0_18:1_18:2	874.78455	[M+NH4]+	8.568	155651552	205746416	1690569	183435984	180471152	88362560	112121432	157328288	52190804	75046448	106202736	45963364	140952288	79746192	61867236	94813176
	TG	TG 52:3|TG 17:1_17:1_18:1	879.73846	[M+Na]+	8.097	248210	566758	3219	520931	293632	82448	136712	298951	73051	94332	159696	58889	458090	107133	51134	125355
	TG	TG 52:4|TG 16:0_18:2_18:2	877.72058	[M+Na]+	8.325	1484655	1996536	1773773	1926305	1756150	832100	1086637	1710285	723578	860621	1232800	703308	1764691	1059084	725960	1119528
	TG	TG 52:4|TG 16:1_18:1_18:2	872.76898	[M+NH4]+	8.327	85586056	131605856	95528392	118439456	99481888	31377636	52981884	89971720	23211564	31990076	52268372	19494568	95154416	37821800	19271680	44015940
	TG	TG 52:4|TG 8:0_22:2_22:2	877.72009	[M+Na]+	7.702	42744	124690	445	144659	1200	20122	519	64363	8612	3307	28762	2552	96519	21114	4956	509
	TG	TG 52:5|TG 16:0_16:0_20:5	875.70306	[M+Na]+	8.062	641850	915678	814631	970186	849381	395703	504510	790668	303149	382996	542612	280095	751471	480826	340747	573207
	TG	TG 52:5|TG 16:1_18:2_18:2	870.75006	[M+NH4]+	8.074	18708458	39376672	25446958	41230736	26201994	7114787	11451833	23314300	4949870	6371618	10960445	3978668	23037346	8549844	4683278	10960147
	TG	TG 52:6|TG 16:0_18:3_18:3	873.69	[M+Na]+	7.786	144879	259734	294861	329844	201744	62031	75086	205888	39309	50353	98596	37292	249443	73969	52057	133594
	TG	TG 52:6|TG 16:1_18:2_18:3	868.73737	[M+NH4]+	7.789	1679144	4117751	2667570	5773926	2534017	611213	962852	2593274	365164	453888	875306	277348	2714516	667876	365220	1058100
	TG	TG 52:6|TG 18:1_16:2_18:3	868.72394	[M+NH4]+	7.985	18465	88960	20598	45204	36393	28794	88025	22418	10824	23927	183756	20174	24003	17004	19052	9067
	TG	TG 53:1|TG 17:0_18:0_18:1	892.8291	[M+NH4]+	9.182	687017	1280920	607528	1076494	1397653	689889	519509	824542	357260	463550	558203	294761	693893	536989	338680	603114
	TG	TG 53:2|TG 17:0_18:1_18:1	890.81494	[M+NH4]+	8.945	5286714	10404123	4539936	7271512	8693996	3827976	3617131	5737331	1269626	1980621	3364820	1002883	4186968	2200361	1691556	3023224
	TG	TG 53:3|TG 17:1_18:1_18:1	888.79956	[M+NH4]+	8.709	5505168	11046572	5512027	7205304	7981942	2750715	3494888	5997610	1284052	1964160	3323108	993682	5137620	2069676	1179885	2895988
	TG	TG 53:4|TG 17:0_17:0_19:4	891.73639	[M+Na]+	8.456	75611	156314	110955	120841	96922	36094	55313	85987	39211	52226	73511	34471	140576	56682	19729	72921
	TG	TG 53:4|TG 17:1_18:1_18:2	886.78333	[M+NH4]+	8.457	2022934	5071118	2517254	3235180	2679346	592247	1153223	2127158	552537	735657	1351306	425140	3198261	825631	329865	1120845
	TG	TG 53:5|TG 17:1_18:2_18:2	884.76782	[M+NH4]+	8.195	333319	1042290	590968	718775	444762	189470	218090	397357	158877	189924	285583	137835	772537	214770	120352	289683
	TG	TG 53:6|TG 17:1_18:2_18:3	882.74591	[M+NH4]+	7.955	48571	145062	104049	168775	97528	35678	50692	82246	29658	31360	49460	25156	127063	39749	26795	59043
**Prenol lipids**	VAE	VAE 21:0	617.5387	[M+Na]+	5.286	2796	5777	836	2523	6472	9912	9153	8934	8331	4649	6063	12730	14902	8033	12944	12284
	VAE	VAE 22:4	623.49573	[M+Na]+	8.249	8148	14382	4157	27848	14515	13955	7866	24593	7766	4574	17977	2567	7057	14619	1601	6981
**Others**	Others	NATau 15:0;O	364.2088	[M-H]-	3.44	6502	5401	4706	4616	4813	4107	3552	3569	4304	2584	3402	5316	7204	3618	2826	2471
	Others	13-Docosenamide	338.34256	[M+H]+	2.862	13978416	13717906	12961020	12553574	22311396	23389574	13507032	20122198	13091348	15479452	28690088	22521286	19741676	15281916	21190580	18797858

Table S3. Significantly changed lipid molecules between the low and high intramuscular fat groups (n=8).

Lipid molecule	Ion mode	*P*-value	FDR	VIP
ASG 28:1;O;Hex;FA 21:0	Negative	0.0092	0.0417	1.64
Cer 12:1;O3/32:5(2OH)	Negative	0.0045	0.0300	1.75
Cer 16:1;O3/38:6(2OH)	Negative	0.0003	0.0074	2.25
Cer 16:2;O3/38:6(2OH)	Negative	0.0012	0.0161	2.28
Cer 18:1;O3/38:6(2OH)	Negative	0.0007	0.0124	1.43
Cer 18:2;O3/38:6(2OH)	Negative	0.0005	0.0090	2.22
Cer 20:1;O3/38:6(2OH)	Negative	0.0015	0.0173	2.00
Cer 20:2;O3/38:6(2OH)	Negative	0.0001	0.0055	1.77
Cer 44:4;O2|Cer 20:1;O2/24:3	Positive	0.0094	0.0421	1.86
Cer 44:4;O2|Cer 28:2;O2/16:2	Positive	0.0061	0.0354	1.51
Cer 45:2;O2|Cer 18:1;O2/27:1	Positive	0.0004	0.0087	1.86
Cer 47:3;O3|Cer 29:3;O2/18:0;O	Positive	0.0016	0.0180	1.40
CL 12:0_15:0_18:0_28:0	Negative	0.0094	0.0421	1.33
CL 12:0_28:0_28:0_28:0	Negative	0.0011	0.0161	1.22
CL 16:0_28:0_28:0_28:0	Negative	0.0003	0.0072	1.59
CL 28:0_16:2_28:0_28:0	Negative	0.0069	0.0367	1.32
DG 30:1	Positive	0.0004	0.0082	1.42
DG 32:1|DG 16:0_16:1	Positive	0.0006	0.0103	1.09
DG 33:0	Positive	0.0023	0.0224	2.15
DG 40:4	Positive	0.0124	0.0460	1.59
DG 45:10	Positive	0.0109	0.0441	1.55
DG 49:10	Positive	0.0075	0.0381	2.62
DG 52:6	Positive	0.0057	0.0337	1.26
FA 16:1	Negative	0.0008	0.0136	1.41
LPG O-24:6	Negative	0.0001	0.0055	1.75
NAGly 28:2;O(FA 28:6)	Negative	0.0006	0.0110	2.46
NAGlySer 20:0;O(FA 28:1)	Negative	0.0000	0.0021	1.38
PC 15:4_24:6	Negative	0.0002	0.0062	1.50
PC 17:0_18:1;O4	Negative	0.0109	0.0441	2.19
PC 30:0	Positive	0.0037	0.0267	1.06
PC 31:0	Positive	0.0039	0.0277	1.00
PC 32:2|PC 16:1_16:1	Positive	0.0003	0.0068	1.25
PC 33:1	Positive	0.0036	0.0267	1.32
PC 33:2|PC 15:0_18:2	Positive	0.0001	0.0055	1.53
PC 34:4	Positive	0.0035	0.0267	1.06
PC 35:1	Positive	0.0064	0.0356	1.59
PC 35:2|PC 17:0_18:2	Positive	0.0000	0.0016	1.29
PC 35:3	Positive	0.0033	0.0260	1.15
PC 36:3|PC 18:1_18:2	Positive	0.0009	0.0141	1.08
PC 36:5|PC 18:2_18:3	Positive	0.0037	0.0267	1.88
PC 37:2|PC 19:0_18:2	Positive	0.0031	0.0255	1.02
PC 38:2	Positive	0.0095	0.0421	1.02
PC 40:6	Positive	0.0077	0.0388	1.82
PC O-32:1|PC O-16:1_16:0	Positive	0.0046	0.0305	1.02
PC O-32:3	Positive	0.0004	0.0087	1.29

PC O-34:4|PC O-18:4_16:0	Positive	0.0107	0.0441	2.09
PC O-37:6|PC O-18:4_19:2	Positive	0.0118	0.0452	2.31
PC O-37:8|PC O-15:2_22:6	Positive	0.0000	0.0016	1.27
PC O-39:3	Positive	0.0028	0.0239	1.21
PE 34:1	Positive	0.0111	0.0441	3.08
PE 36:4|PE 18:2_18:2	Positive	0.0025	0.0226	1.22
PE 38:5|PE 18:0_20:5	Negative	0.0089	0.0410	1.06
PE 42:0_6:0	Negative	0.0001	0.0061	2.30
PE 6:0_42:1	Negative	0.0005	0.0093	2.09
PE O-16:0_28:0;O1	Negative	0.0000	0.0006	4.11
PE O-18:0_28:0;O1	Negative	0.0001	0.0055	1.74
PE O-35:5|PE O-15:1_20:4	Negative	0.0014	0.0170	1.11
PE O-37:6|PE O-17:2_20:4	Negative	0.0010	0.0148	1.15
PE P-38:4|PE P-18:0_20:4	Positive	0.0019	0.0196	1.09
PG O-15:0_28:3	Negative	0.0013	0.0164	4.15
PG O-15:0_28:7	Negative	0.0026	0.0226	1.10
PS 36:0|PS 18:0_18:0	Negative	0.0083	0.0402	1.45
PS 36:1|PS 18:0_18:1	Negative	0.0004	0.0088	1.17
PS 40:5	Negative	0.0002	0.0062	1.37
PS O-8:0_16:2	Negative	0.0100	0.0425	2.83
SM 12:1;O2/35:0	Negative	0.0000	0.0021	1.46
SM 33:1;O2	Positive	0.0121	0.0454	1.11
SM 34:0;O2	Positive	0.0121	0.0454	1.04
SM 40:1;O2	Positive	0.0131	0.0476	1.89
SM 42:2;O2|SM 18:1;O2/24:1	Positive	0.0110	0.0441	1.11
SM 42:3;O2	Positive	0.0097	0.0422	1.99
SM 45:0;O3	Negative	0.0036	0.0267	3.36
SM 45:1;O3	Negative	0.0109	0.0441	1.14
TG 40:1|TG 8:0_14:0_18:1	Positive	0.0009	0.0136	1.07
TG 40:2|TG 8:0_16:1_16:1	Positive	0.0011	0.0161	2.01
TG 42:1|TG 10:0_16:0_16:1	Positive	0.0001	0.0055	1.46
TG 42:2|TG 10:0_16:1_16:1	Positive	0.0037	0.0267	1.59
TG 42:2|TG 8:0_16:1_18:1	Positive	0.0002	0.0061	1.83
TG 42:3|TG 8:0_10:0_24:3	Positive	0.0033	0.0260	1.45
TG 44:2|TG 10:0_16:0_18:2	Positive	0.0001	0.0055	1.60
TG 44:3|TG 10:0_16:1_18:2	Positive	0.0001	0.0055	1.82
TG 44:3|TG 8:0_18:1_18:2	Positive	0.0034	0.0267	1.66
TG 46:2;O3|TG 14:0_14:0_18:2;O3	Positive	0.0086	0.0410	3.01
TG 46:2|TG 16:0_14:1_16:1	Positive	0.0001	0.0055	1.28
TG 46:3|TG 14:1_14:1_18:1	Positive	0.0025	0.0226	1.11
TG 46:3|TG 14:1_16:1_16:1	Positive	0.0002	0.0062	1.83
TG 46:4|TG 10:0_18:1_18:3	Positive	0.0012	0.0161	1.78
TG 46:4|TG 14:1_14:1_18:2	Positive	0.0051	0.0318	2.24
TG 48:1|TG 14:0_14:0_20:1	Positive	0.0037	0.0267	1.36
TG 48:2|TG 14:0_16:1_18:1	Positive	0.0001	0.0055	1.08
TG 48:3|TG 14:0_16:1_18:2	Positive	0.0003	0.0069	1.49
TG 48:4|TG 14:0_14:0_20:4	Positive	0.0086	0.0410	1.14

TG 48:4|TG 14:1_16:1_18:2	Positive	0.0013	0.0164	1.88
TG 48:5|TG 14:1_16:1_18:3	Positive	0.0088	0.0410	1.16
TG 50:2;O|TG 16:0_18:1_16:1;O	Positive	0.0078	0.0390	1.82
TG 50:3|TG 16:0_16:1_18:2	Positive	0.0002	0.0062	1.18
TG 50:5|TG 14:0_18:2_18:3	Positive	0.0066	0.0360	1.45
TG 51:2|TG 16:0_17:1_18:1	Positive	0.0000	0.0021	1.17
TG 51:3|TG 16:0_17:1_18:2	Positive	0.0016	0.0184	1.16
TG 52:4;O|TG 17:0_18:2_17:2;O	Positive	0.0137	0.0494	2.09
TG 52:5|TG 16:1_18:2_18:2	Positive	0.0088	0.0410	1.13
TG 52:6|TG 16:1_18:2_18:3	Positive	0.0099	0.0422	1.46
TG 53:2|TG 17:0_18:1_18:1	Positive	0.0003	0.0069	1.16
TG 53:3|TG 17:1_18:1_18:1	Positive	0.0015	0.0174	1.19
VAE 21:0	Positive	0.0073	0.0380	1.62

Abbreviations: FDR, false discovery rate; VIP, variable importance in projection.

Table S4. Significantly changed and FC>2/FC<0.5 lipid molecules between the low and high intramuscular fat groups (n=8).

Metabolite name	FC (H/L)	log2(FC)	FDR	VIP
PE O-16:0_28:0;O1	12.06	3.59	0.0006	1.46
Cer 18:2;O3/38:6(2OH)	4.07	2.02	0.0090	1.06
Cer 16:1;O3/38:6(2OH)	3.99	2.00	0.0074	1.02
PE 42:0_6:0	3.86	1.95	0.0061	1.46
PE 6:0_42:1	3.41	1.77	0.0093	1.46
Cer 20:2;O3/38:6(2OH)	3.15	1.65	0.0055	1.06
PE O-18:0_28:0;O1	3.04	1.61	0.0055	1.49
TG 46:3|TG 14:1_16:1_16:1	3.01	1.59	0.0062	2.01
TG 44:3|TG 10:0_16:1_18:2	2.93	1.55	0.0055	1.88
TG 42:2|TG 8:0_16:1_18:1	2.85	1.51	0.0061	1.83
LPG O-24:6	2.73	1.45	0.0055	1.16
CL 16:0_28:0_28:0_28:0	2.60	1.38	0.0072	1.11
TG 44:2|TG 10:0_16:0_18:2	2.53	1.34	0.0055	1.86
TG 48:3|TG 14:0_16:1_18:2	2.48	1.31	0.0069	2.19
SM 12:1;O2/35:0	2.41	1.27	0.0021	1.62
DG 30:1	2.40	1.26	0.0082	1.11
TG 42:1|TG 10:0_16:0_16:1	2.34	1.22	0.0055	1.82
NAGlySer 20:0;O(FA 28:1)	2.28	1.19	0.0021	1.17
TG 46:2|TG 16:0_14:1_16:1	2.17	1.12	0.0055	1.99
TG 50:3|TG 16:0_16:1_18:2	2.06	1.04	0.0062	2.30
TG 51:2|TG 16:0_17:1_18:1	2.01	1.01	0.0021	2.46
PC 32:2|PC 16:1_16:1	0.48	-1.06	0.0068	1.22
PC O-37:8|PC O-15:2_22:6	0.46	-1.11	0.0016	1.42
PC 35:2|PC 17:0_18:2	0.45	-1.14	0.0016	1.28
PC O-32:3	0.44	-1.19	0.0087	1.38
PS 40:5	0.43	-1.23	0.0062	1.59
PC 15:4_24:6	0.41	-1.29	0.0062	1.17
PC 33:2|PC 15:0_18:2	0.41	-1.30	0.0055	1.25
Cer 45:2;O2|Cer 18:1;O2/27:1	0.37	-1.43	0.0087	1.08

Abbreviations: H, high intramuscular fat group; L, low intramuscular fat group.

Table S5. Aroma compounds identified in donkey meats (n=8).

Compound class	LRI		[3]Concentration (μg/kg)		VIP
	[1]Calculated RI	[2]Library RI	HIMF	LIMF	
Aldehydes					
(*E*)-2-Undecenal	1750	1766	3.01±0.37	3.22±0.39	0.03
2,4-Decadienal, (*E,E*)-	1763	1812	4.55±0.76[b]	8.26±0.63[a]	0.75
2,4-Heptadienal, (*E,E*)-	1459	1463	0.44±0.07	0.57±0.05	0.12
2-Butenal, 3-methyl-	1197	1215	2.19±0.22	2.42±0.14	0.02
2-Decenal, (*E*)-	1642	1659	3.84±0.44	3.88±0.44	0.1
2-Heptenal, (*E*)-	1323	1326	60.26±9.73	56.76±4.75	0.32
2-Hexenal, (*E*)-	1217	1230	0.53±0.05[a]	0.33±0.02[b]	0.32
2-Nonenal, (*E*)-	1534	1536	3.33±0.49	3.65±0.37	0.03
2-Octenal, (*E*)-	1428	1430	41.34±6.92	42.68±4.17	0.06
2-Octenal, 2-butyl-	1666	1682	2.24±0.75[b]	18.19±3.36[a]	3.05
2-Pentenal, (*E*)-	1127	1127	3.76±0.41[a]	2.45±0.43[b]	0.81
5-Ethyl-2-furaldehyde	1637	1645	0.27±0.05	0.34±0.04	0.09
Benzaldehyde	1526	1531	397.68±48.22	527.61±61.23	0.96
Benzaldehyde, 2-hydroxy-	1683	1672	3.32±0.30	3.45±0.20	0.06
Benzaldehyde, 2-methyl-	1622	1632	0.32±0.03	0.33±0.01	0.02
Benzaldehyde, 3,5-dimethyl-	1818	1837	0.14±0.01	0.12±0.01	0.1
Benzaldehyde, 4-ethyl-	1711	1745	10.5±1.66[b]	15.63±1.60[a]	0.61
Benzaldehyde, 4-pentyl-	2008	2003	1.64±0.33[b]	4.23±0.48[a]	0.96
Benzeneacetaldehyde	1645	1651	0.84±0.07[a]	0.63±0.06[b]	0.27
Butanal	860	883	2.85±0.29	2.33±0.14	0.29
Decanal	1496	1500	4.53±0.41[b]	5.93±0.18[a]	0.23
Dodecanal	1707	1710	3.34±0.50[b]	7.8±0.81[a]	1.01
E-Citral	1729	1744	0.4±0.07[b]	1.12±0.10[a]	0.65
Furfural	1463	1465	1.17±0.11	0.99±0.07	0.2
Heptanal	1182	1185	111.73±17.50	116.4±10.28	0.51
Hexadecanal	2130	2149	1.15±0.08	1.59±0.28	0.14
Hexanal	1083	1086	2344.96±274.77	2235.94±134.23	1.12
Nonanal	1392	1396	366.76±36.15	447.64±21.26	0.66
Octanal	1287	1289	112.17±6.44[a]	89.13±9.67[b]	1.24
Pentanal	1008	969	431.81±30.76[a]	338.9±18.5[b]	1.82
Trans,trans-Nona-2,4-dienal	1700	1713	0.99±0.19	1.32±0.17	0.18
Undecanal	1601	1616	5.82±1.10[b]	8.68±0.49[a]	0.54
3-Cyclohexene-1-carboxaldehyde	1459	1490	3.56±1.13	3.18±0.84	0.15
5-Ethylcyclopent-1-enecarboxaldehyde	1418	1410	31.79±4.32	27.18±2.09	0.52
Ketones					
1-Octen-3-one	1298	1310	3.65±0.30[a]	2.33±0.15[b]	0.58
1-Propanone, 1-(1-cyclohexen-1-yl)-	1590	1620	1.26±0.21[b]	3.45±0.34[a]	0.92
2(3H)-Furanone, 5-butyldihydro-	1920	1938	0.03±0.01	0.03±0.01	0.07
2(3H)-Furanone, 5-ethyldihydro-	1706	1723	0.032±0.00[a]	0.025±0.00[b]	0.08
2(3H)-Furanone, dihydro-5-pentyl-	2033	2051	0.06±0.01	0.05±0.00	0.07
2(3H)-Furanone, dihydro-5-propyl-	1808	1817	0.02±0.00	0.02±0.00	0.09
2,3-Octanedione	1320	1323	724.27±160.99	980.71±61.07	2.4
2,3-Pentanedione	1063	1065	2.45±0.79	4.00±0.45	1.06

2,5-Hexanedione	1502	1500	0.14±0.09	0.03±0.00	0.32
2-Butanone	889	908	5.11±2.29[a]	0.18±0.03[b]	1.96
2-Heptanone	1178	1181	21.18±1.91	22.77±1.4	0.02
2-Heptanone, 4,6-dimethyl-	1257	1262	16.26±1.26[b]	18.14±0.74[a]	0.85
2-Heptanone, 6-methyl-	1234	1245	0.51±0.15	1.10±0.14	0.04
2-Nonanone	1386	1397	0.73±0.05	0.66±0.10	0.58
2-Octanone	1282	1293	1.48±0.25[b]	2.2±0.11[a]	0.33
2-Pyrrolidinone	2036	2043	0.48±0.14	0.28±0.04	0.27
2-Pyrrolidinone, 1-methyl-	1672	1665	0.06±0.02[a]	0.02±0.00[b]	0.27
3(2H)-Furanone, 4-hydroxy-5-methyl-	2112	2120	0.27±0.26	0.005±0.00	0.42
3,5-Octadien-2-one	1516	1522	6.15±1.28[b]	9.56±0.78[a]	0.68
3-Octanone	1251	1264	3.51±0.56	4.77±0.75	0.01
3-Octen-2-one	1405	1407	8.04±2.77	11.41±1.89	0.66
5,9-Undecadien-2-one, 6,10-dimethyl-, (Z)-	1849	1863	2.39±0.21[b]	4.88±0.30[a]	0.04
6-Methyl-5-hepten-2-one	1334	1335	12.85±12.31	13.91±13.00	0.25
6-Undecanone	1524	1528	0.14±0.03[b]	0.6±0.06[a]	0.73
Acetoin	1283	1298	0.49±0.29	0.47±0.14	0.27
Cyclohexanone	1293	1291	0.25±0.02	0.3±0.08	0.01
Ethanone, 1,1'-(1,3-phenylene)bis-	2338	2333	0.03±0.01	0.03±0.01	0.04
Isophorone	1595	1591	0.13±0.05	0.05±0.01	0.3
2,6-Di-tert-butyl-4-hydroxy-4-methylcyclohexa-2,5-dien-1-one	2086	2116	15±6.40	9.32±1.97	0.46
Alcohols					
1-Pentanol	1240	1253	219.82±22.28[a]	157.22±8.62[b]	1.73
1-Octen-3-ol	1439	1451	137.01±20.37	150.08±6.28	0.21
(S)-(+)-3-Methyl-1-pentanol	1535	1557	9.36±2.45	7.6±0.52	0.21
1-Hexanol	1343	1355	9.00±1.20	8.63±0.37	0.15
1-Decanol	1763	1760	0.98±0.21[b]	2.01±0.20[a]	0.6
1-Dodecanol	1955	1969	0.98±0.18	1.34±0.23	0.16
Benzenemethanol	1872	1890	0.8±0.07	0.66±0.09	0.21
Ethanol, 2-phenoxy-	2140	2121	0.14±0.03	0.12±0.02	0.1
5-Octen-1-ol, (Z)-	1604	1615	1.34±0.27	1.86±0.11	0.28
1,6-Heptadien-4-ol	1312	1330	1.96±0.31[a]	1.00±0.05[b]	0.62
Nerol	1834	1850	0.31±0.03[b]	0.41±0.03[a]	0.1
Acids					
Hexanoic acid	1841	1864	18.97±1.72	22.97±1.83	0.2
Acetic acid	1456	1449	3.60±1.22	1.89±0.53	0.72
Decanoic acid	2265	2289	2.91±0.28[a]	1.8±0.12[b]	0.57
Octanoic acid	2053	2065	2.03±0.15[a]	1.45±0.10[b]	0.4
Heptanoic acid	1947	1970	1.45±0.17	1.08±0.07	0.32
Pentanoic acid	1735	1733	0.76±0.04[a]	0.64±0.03[b]	0.18
Butanoic acid	1627	1649	0.56±0.15	0.29±0.04[b]	0.37
Hexanoic acid, 2-ethyl-	1942	1964	0.20±0.03[a]	0.25±0.07[b]	0
Dodecanoic acid	2476	2497	0.18±0.04[a]	0.09±0.01[b]	0.29
Esters					
Formic acid, octyl ester	1444	1428	29.03±2.97[a]	20.13±1.12[b]	0.94

1,2-Benzenedicarboxylic acid, bis(2-methylpropyl) ester	2536	2536	15.12±2.4	13.51±1.74	0.37
n-Caproic acid vinyl ester	1222	1233	0.76±0.21	1.19±0.97	0.34
Decanoic acid, ethyl ester	1633	1644	0.21±0.08	0.12±0.03	0.24
Hexadecanoic acid, methyl ester	2210	2208	0.14±0.01	0.2±0.03	0.08
Nonanoic acid, ethyl ester	1531	1531	0.37±0.33	0.1±0.08	0.38
Pentanoic acid, pentyl ester	1410	1401	0.06±0.01[b]	0.2±0.02[a]	0.42
Acetic acid, cyclohexyl ester	1352	1361	0.07±0.02[b]	0.2±0.01[a]	0.54
Benzoic acid, 2-hydroxy-, methyl ester	1781	1796	0.15±0.01	0.13±0.01	0.08
Octanoic acid, ethyl ester	1431	1440	0.19±0.15	0.08±0.05	0.23
Nonanoic acid, methyl ester	1488	1491	0.14±0.07	0.18±0.12	0.01
Propanoic acid, 2-methyl-, butyl ester	1151	1148	0.27±0.12	0.04±0.02	0.72
Decanoic acid, methyl ester	1590	1593	0.07±0.03	0.06±0.02	0.08
Hexanedioic acid, bis(2-methylpropyl) ester	2119	2126	0.04±0.01	0.03±0.00	0.06
Butanoic acid, butyl ester	1214	1220	0.09±0.03[a]	0.02±0.01[b]	0.52
Dodecanoic acid, ethyl ester	1837	1841	0.02±0.01	0.02±0.01	0.07
Trans-Methyl Dihydrojasmonate	2281	2264	0.05±0.02	0.05±0.01	0.01
1-Methoxy-2-propyl acetate	1217	1233	0.12±0.06	0.08±0.05	0.3
Triacetin	2059	2077	0.11±0.03	0.04±0.00	0.29
Diethyl Phthalate	2369	2366	0.16±0.05	0.13±0.02	0.1
Anisyl propionate	2168	2190	0.18±0.04	0.2±0.03	0.02
Tributyl phosphate	2108	2114	0.23±0.05	0.22±0.10	0.15
Dimethyl phthalate	2299	2303	0.6±0.17	0.51±0.07	0.11
Hexanoic acid, thio-, S-butyl ester	1578	1608	1.45±0.33[b]	3.64±0.34[a]	0.91
Hexanethioic acid, S-methyl ester	1402	1412	0.34±0.06[b]	0.52±0.05[a]	0.34
Furans					
Furan, 3-methyl-	854	853	0.03±0.02[b]	0.07±0.01[a]	0.47
Furan, 2-ethyl-	978	960	5.83±0.70	6.17±0.45	0.02
Furan, 2-pentyl-	1230	1232	118.4±28.79[b]	287.83±51.79[a]	3.46
trans-Linalool oxide (furanoid)	1466	1452	1.95±0.40[b]	4.52±0.31[a]	0.92
1-Propanone, 1-(5-methyl-2-furanyl)-	1712	1670	0.25±0.05[b]	0.38±0.03[a]	0.19
1-Pentanone, 1-(2-furanyl)-	1760	1747	0.17±0.04[b]	0.49±0.05[a]	0.53
2-Butylfuran	1128	1142	4.81±2.97	0.15±0.01	1.43
Furan, 2-hexyl-	1330	1321	0.12±0.03	0.24±0.18	0.08
Sulfur-containing compounds					
Dimethyl sulfone	1902	1913	2.65±0.61[a]	1.08±0.17[b]	0.85
Thiophene, 2-pentyl-	1460	1473	3.59±0.71[b]	11.06±1.13[a]	1.54
5-Methyl-2-thiophenecarboxaldehyde	1732	1767	3.88±0.56	2.63±0.54	0.65
2-Thiophenecarboxaldehyde	1697	1684	0.22±0.02	0.21±0.01	0.05
Thiophene, 2-methyl-	1117	1122	0.04±0.01	0.04±0.01	0.01
Propanal, 3-(methylthio)-	1455	1457	1.38±0.35[a]	0.58±0.05[b]	0.65
2-Acetyl-2-thiazoline	1761	1763	18.48±2.90[a]	10.9±1.43[b]	1.2
Benzothiazole	1963	1967	0.21±0.02	0.2±0.02	0.07
Nitrogen-containing compounds					
Ethanone, 1-(2-pyridinyl)-	1603	1606	2.92±2.03[b]	110.24±8.89[a]	7.98
Pyridine, 2-pentyl-	1570	1584	2.26±0.30[b]	3.68±0.22[a]	0.45

Formamide, N,N-dibutyl-	1767	1757	1.41±0.22	2.26±0.39	0.35
Pyridine, 3-ethyl-	1377	1377	0.4±0.04	0.34±0.02	0.12
Pyrazine, 2,3-dimethyl-	1318	1325	0.36±0.15[a]	0.02±0.00[b]	0.81
Pyridine, 2-ethyl-	1278	1278	0.09±0.01	0.09±0.01	0
Hydrocarbons					
(3Z,5E)-1,3,5-Undecatriene	1404	1408	3.36±0.89[b]	7.3±0.80[a]	1.01
4-Octyne	980	1000	5.17±0.63	6.17±0.42	0.15
Benzene	959	949	2.37±0.33	2.33±0.15	0.07
Benzene, 1,2,3,5-tetramethyl-	1414	1422	0.23±0.03[a]	0.16±0.01[b]	0.21
Benzene, 1,2,3-trimethyl-	1340	1340	0.46±0.06	0.32±0.04	0.26
Benzene, 1,3-bis(1,1-dimethylethyl)-	1427	1427	2.13±0.67	1.4±0.64	0.53
Benzene, 1,3-dimethyl-	1186	1143	11.72±2.49[a]	5.02±0.96[b]	1.32
Benzene, 1-ethyl-2,3-dimethyl-	1371	1369	0.84±0.10[a]	0.51±0.05[b]	0.4
Benzene, 1-ethyl-2,4-dimethyl-	1355	1348	0.18±0.03[a]	0.1±0.01[b]	0.27
Benzene, 1-ethyl-2-methyl-	1263	1258	1.17±0.25	0.96±0.10	0.19
Benzene, 1-ethyl-3,5-dimethyl-	1327	1319	0.59±0.09[a]	0.32±0.04[b]	0.4
Benzene, 1-methyl-3-propyl-	1305	1297	1.46±0.29[a]	0.5±0.08[b]	0.87
Benzene, 2-ethyl-1,3-dimethyl-	1362	1359	1.34±0.18[a]	0.86±0.03[b]	0.41
Decane	1021	1000	4.78±1.42[a]	0.96±0.11[b]	1.52
Decane, 5-methyl-	1055	1049	0.26±0.11	0.05±0.01	0.63
Dodecane	1197	1200	9.67±2.96	5.85±0.97	0.52
Ethylbenzene	1132	1129	13.6±2.38[a]	6.16±1.73[b]	1.43
Mesitylene	1283	1251	9.65±1.94	7.8±1.17	0.45
Octane, 2,4,6-trimethyl-	1043	1058	14.43±5.35[a]	2.57±0.35[b]	2.23
o-Xylene	1184	1186	2.94±0.26[a]	1.31±0.22[b]	0.89
p-Xylene	1135	1138	27.52±6.47[a]	5.21±2.01[b]	2.98
Styrene	1257	1270	10.32±2.23[b]	17.18±1.84[a]	0.84
Toluene	1052	1049	38.62±4.75	36.21±4.21	0.4
Tridecane	1292	1300	1.74±0.15	1.45±0.12	0.26
Others					
1,8-Cineole	1207	1213	0.1±0.02	0.15±0.02	0.11
Butylated Hydroxytoluene	1908	1909	2.36±0.67	1.39±0.19	0.47
p-Cresol	2079	2089	1.07±0.41	0.62±0.05	0.31
Phenol	2003	2014	0.39±0.04[a]	0.27±0.01[b]	0.21

[1] Calculated data based on a homologous series of n-alkanes (C7-C40).

[2] Data obtained from the online database: http://www.flavornet.org/, https://pubchem.ncbi.nlm.nih.gov/.

[3] Different letters in the same row represent significant differences.

HIMF, high intramuscular fat group; LIMF, low intramuscular fat group; LRI, Linear retention index; VIP, variable importance in projection.

Table S6. Odor thresholds and odor active values of odorants in low and high intramuscular fat groups (n=8).

Class and Component Name	Concentration (μg/kg)		Odor threshold[1]	Odor active values	
	HIMF	LIMF		HIMF	LIMF
Aldehydes					
Pentanal	431.81	338.90	12	36	28
Octanal	112.17	89.13	3.4	33	26
2,4-Heptadienal,(*E*,*E*)-	0.44	0.57	N/A	N/A	N/A
2,4-Decadienal, (*E*,*E*)-	4.55	8.26	0.027	169	306
2-Butenal, 3-methyl-	2.19	2.42	N/A	N/A	N/A
2-Decenal, (*E*)-	3.84	3.88	0.3	13	13
2-Hexenal, (*E*)-	0.53	0.33	82	0	0
2-Octenal, (*E*)-	41.34	42.68	4	10	11
2-Nonenal, (*E*)-	3.33	3.65	0.09	37	41
Hexanal	2344.96	2235.94	5	469	447
Nonanal	366.76	447.64	1.1	333	407
Trans,trans-Nona-2,4-dienal	0.99	1.32	0.1	10	13
2-Octenal, 2-butyl-	2.24	18.19	N/A	N/A	N/A
2-Pentenal, (*E*)-	3.76	2.45	310	0	0
5-Ethyl-2-furaldehyde	0.27	0.34	N/A	N/A	N/A
Benzaldehyde	397.68	527.61	350	1	2
Benzaldehyde, 2-hydroxy-	3.32	3.45	30	0	0
Benzaldehyde, 2-methyl-	0.32	0.33	N/A	N/A	N/A
Benzaldehyde, 3,5-dimethyl-	0.14	0.12	N/A	N/A	N/A
Benzaldehyde, 4-ethyl-	10.50	15.63	N/A	N/A	N/A
Benzaldehyde, 4-pentyl-	1.64	4.23	N/A	N/A	N/A
Benzeneacetaldehyde	0.84	0.63	2	0	0
Butanal	2.85	2.33	8.2	0	0
2-Heptenal, (E)-	60.26	56.76	13	5	4
Dodecanal	3.34	7.80	10	0	1
E-Citral	0.40	1.12	32	0	0
Furfural	1.17	0.99	282	0	0
Heptanal	111.73	116.40	2.8	40	42
(*E*)-2-Undecenal	3.01	3.22	0.78	4	4
Hexadecanal	1.15	1.59	N/A	N/A	N/A
Decanal	4.53	5.93	3	2	2
Undecanal	5.82	8.68	12.5	0	1
3-Cyclohexene-1-carboxaldehyde	3.56	3.18	N/A	N/A	N/A
5-Ethylcyclopent-1-enecarboxaldehyde	31.79	27.18	N/A	N/A	N/A
Ketones					
1-Octen-3-one	3.65	2.33	0.016	228	146
1-Propanone, 1-(1-cyclohexen-1-yl)-	1.26	3.45	N/A	N/A	N/A
2(3H)-Furanone, 5-butyldihydro-	0.03	0.03	6.5	0	0
2(3H)-Furanone, 5-ethyldihydro-	0.03	0.03	260	0	0
2(3H)-Furanone, dihydro-5-pentyl-	0.06	0.05	9.7	0	0
2(3H)-Furanone, dihydro-5-propyl-	0.02	0.02	400	0	0
3(2H)-Furanone, 4-hydroxy-5-methyl-	0.27	0.00	N/A	N/A	N/A

2,3-Octanedione	724.27	980.71	N/A	N/A	N/A
2,3-Pentanedione	2.45	4.00	20	0	0
2,5-Hexanedione	0.14	0.03	N/A	N/A	N/A
2-Butanone	5.11	0.18	35000	0	0
2-Heptanone	21.18	22.77	140	0	0
2-Heptanone, 4,6-dimethyl-	16.26	18.14	N/A	N/A	N/A
2-Heptanone, 6-methyl-	0.51	1.10	8.1	0	0
2-Nonanone	0.73	0.66	5	0	0
2-Octanone	1.48	2.20	5	0	0
2-Pyrrolidinone	0.48	0.28	N/A	N/A	N/A
2-Pyrrolidinone, 1-methyl-	0.06	0.02	N/A	N/A	N/A
3,5-Octadien-2-one	6.15	9.56	N/A	N/A	N/A
3-Octanone	3.51	4.77	21.4	0	0
3-Octen-2-one	8.04	11.41	N/A	N/A	N/A
5,9-Undecadien-2-one, 6,10-dimethyl-, (Z)-	2.39	4.88	N/A	N/A	N/A
6-Methyl-5-hepten-2-one	12.85	13.91	50	0	0
6-Undecanone	0.14	0.60	85	0	0
Acetoin	0.49	0.47	14	0	0
Cyclohexanone	0.25	0.30	280	0	0
Ethanone, 1,1'-(1,3-phenylene)bis-	0.03	0.03	N/A	N/A	N/A
Isophorone	0.13	0.05	11000	0	0
2,6-Di-tert-butyl-4-hydroxy-4-methylcyclohexa-2,5-dien-1-one	15.00	9.32	N/A	N/A	N/A
Alcohols					
1-Pentanol	219.82	157.22	150	1	1
1-Octen-3-ol	137.01	150.08	45	3	3
(S)-(+)-3-Methyl-1-pentanol	9.36	7.60	N/A	N/A	N/A
1-Hexanol	9.00	8.63	500	0	0
1-Decanol	0.98	2.01	47	0	0
1-Dodecanol	0.98	1.34	16	0	0
Benzenemethanol	0.80	0.66	2500	0	0
Ethanol, 2-phenoxy-	0.14	0.12	N/A	N/A	N/A
5-Octen-1-ol, (Z)-	1.34	1.86	6	0	0
1,6-Heptadien-4-ol	1.96	1.00	N/A	N/A	N/A
Nerol	0.31	0.41	680	0	0
Acids					
Hexanoic acid	18.97	22.97	3000	0	0
Acetic acid	3.60	1.89	99000	0	0
Decanoic acid	2.91	1.80	130	0	0
Octanoic acid	2.03	1.45	1400	0	0
Heptanoic acid	1.45	1.08	640	0	0
Pentanoic acid	0.76	0.64	24	0	0
Butanoic acid	0.56	0.29	204	0	0
Hexanoic acid, 2-ethyl-	0.20	0.25	27000	0	0
Dodecanoic acid	0.18	0.09	N/A	N/A	N/A
Esters					
Formic acid, octyl ester	29.03	20.13	N/A	N/A	N/A

1,2-Benzenedicarboxylic acid, bis(2-methylpropyl) ester	15.12	13.51	N/A	N/A	N/A
n-Caproic acid vinyl ester	0.76	1.19	N/A	N/A	N/A
Decanoic acid, ethyl ester	0.21	0.12	5	0	0
Hexadecanoic acid, methyl ester	0.14	0.20	2000	0	0
Nonanoic acid, ethyl ester	0.37	0.10	370	0	0
Pentanoic acid, pentyl ester	0.06	0.20	26	0	0
Acetic acid, cyclohexyl ester	0.07	0.20	N/A	N/A	N/A
Benzoic acid, 2-hydroxy-, methyl ester	0.15	0.13	40	0	0
Octanoic acid, ethyl ester	0.19	0.08	19	0	0
Nonanoic acid, methyl ester	0.14	0.18	N/A	N/A	N/A
Propanoic acid, 2-methyl-, butyl ester	0.27	0.04	93	0	0
Decanoic acid, methyl ester	0.07	0.06	4.3	0	0
Hexanedioic acid, bis(2-methylpropyl) ester	0.04	0.03	N/A	N/A	N/A
Butanoic acid, butyl ester	0.09	0.02	400	0	0
Dodecanoic acid, ethyl ester	0.02	0.02	400	0	0
Trans-Methyl Dihydrojasmonate	0.05	0.05	N/A	N/A	N/A
1-Methoxy-2-propyl acetate	0.12	0.08	N/A	N/A	N/A
Triacetin	0.11	0.04	N/A	N/A	N/A
Diethyl Phthalate	0.16	0.13	N/A	N/A	N/A
Anisyl propionate	0.18	0.20	N/A	N/A	N/A
Tributyl phosphate	0.23	0.22	N/A	N/A	N/A
Dimethyl phthalate	0.60	0.51	N/A	N/A	N/A
Hexanoic acid, thio-, S-butyl ester	1.45	3.64	N/A	N/A	N/A
Hexanethioic acid, S-methyl ester	0.34	0.52	0.3	1	2
Furans					
Furan, 2-pentyl-	118.40	287.83	5.8	20	50
Furan, 3-methyl-	0.03	0.07	N/A	N/A	N/A
Furan, 2-ethyl-	5.83	6.17	N/A	N/A	N/A
trans-Linalool oxide (furanoid)	1.95	4.52	60	0	0
1-Propanone, 1-(5-methyl-2-furanyl)-	0.25	0.38	N/A	N/A	N/A
1-Pentanone, 1-(2-furanyl)-	0.17	0.49	N/A	N/A	N/A
2-Butylfuran	4.81	0.15	5	1	0
Furan, 2-hexyl-	0.12	0.24	N/A	N/A	N/A
Sulfur-containing compounds					
Dimethyl sulfone	2.65	1.08	N/A	N/A	N/A
Thiophene, 2-pentyl-	3.59	11.06	N/A	N/A	N/A
5-Methyl-2-thiophenecarboxaldehyde	3.88	2.63	N/A	N/A	N/A
2-Thiophenecarboxaldehyde	0.22	0.21	5000	0	0
Thiophene, 2-methyl-	0.04	0.04	N/A	N/A	N/A
2-Acetyl-2-thiazoline	18.48	10.90	0.12	154	91
Benzothiazole	0.21	0.20	80	0	0
Propanal, 3-(methylthio)-	1.38	0.58	0.2	7	3
Nitrogen-containing compounds					
Pyridine, 2-pentyl-	2.26	3.68	0.6	4	6
Ethanone, 1-(2-pyridinyl)-	2.92	110.24	19	0	6
Formamide, N,N-dibutyl-	1.41	2.26	N/A	N/A	N/A
Pyridine, 3-ethyl-	0.40	0.34	N/A	N/A	N/A

Pyrazine, 2,3-dimethyl-	0.36	0.02	400	0	0
Pyridine, 2-ethyl-	0.09	0.09	N/A	N/A	N/A
Hydrocarbons					
(3Z,5E)-1,3,5-Undecatriene	3.36	7.30	N/A	N/A	N/A
4-Octyne	5.17	6.17	N/A	N/A	N/A
Benzene	2.37	2.33	72	0	0
Benzene, 1,2,3,5-tetramethyl-	0.23	0.16	N/A	N/A	N/A
Benzene, 1,2,3-trimethyl-	0.46	0.32	N/A	N/A	N/A
Benzene, 1,3-bis(1,1-dimethylethyl)-	2.13	1.40	N/A	N/A	N/A
Benzene, 1,3-dimethyl-	11.72	5.02	N/A	N/A	N/A
Benzene, 1-ethyl-2,3-dimethyl-	0.84	0.51	N/A	N/A	N/A
Benzene, 1-ethyl-2,4-dimethyl-	0.18	0.10	N/A	N/A	N/A
Benzene, 1-ethyl-2-methyl-	1.17	0.96	N/A	N/A	N/A
Benzene, 1-ethyl-3,5-dimethyl-	0.59	0.32	N/A	N/A	N/A
Benzene, 1-methyl-3-propyl-	1.46	0.50	N/A	N/A	N/A
Benzene, 2-ethyl-1,3-dimethyl-	1.34	0.86	N/A	N/A	N/A
Decane	4.78	0.96	130	0	0
Decane, 5-methyl-	0.26	0.05	N/A	N/A	N/A
Dodecane	9.67	5.85	10000	0	0
Ethylbenzene	13.60	6.16	22000	0	0
Mesitylene	9.65	7.80	700	0	0
Octane, 2,4,6-trimethyl-	14.43	2.57	N/A	N/A	N/A
o-Xylene	2.94	1.31	450	0	0
p-Xylene	27.52	5.21	1000	0	0
Styrene	10.32	17.18	65	0	0
Toluene	38.62	36.21	527	0	0
Tridecane	1.74	1.45	N/A	N/A	N/A
Others					
1,8-Cineole	0.10	0.15	1.1	0	0
Butylated Hydroxytoluene	2.36	1.39	1	2	1
p-Cresol	1.07	0.62	3.9	0	0
Phenol	0.39	0.27	500	0	0

[1]Odor threshold in water obtained from the literature [van Gemert, L. J. (2011). Compilations of odour threshold values in air, water and other media. (Edition 2011 ed.). The Netherlands: Oliemans Punter & Partners BV, Zeist.]

HIMF, high intramuscular fat group; LIMF, low intramuscular fat group. N/A, Not Available

Table S7. Odorants (OAV>1) in the low and high intramuscular fat groups (n=8).

NO.	Odorants name	OAV	
		HIMF	LIMF
Odor1	Hexanal	469	447
Odor2	Nonanal	333	407
Odor3	1-Octen-3-one	228	146
Odor4	2,4-Decadienal, (*E*,*E*)-	169	306
Odor5	2-Acetyl-2-thiazoline	154	91
Odor6	Heptanal	40	42
Odor7	2-Nonenal, (*E*)-	37	41
Odor8	Pentanal	36	28
Odor9	Octanal	33	26
Odor10	Furan, 2-pentyl-	20	50
Odor11	2-Decenal, (*E*)-	13	13
Odor12	2-Octenal, (*E*)-	10	11
Odor13	Trans,trans-Nona-2,4-dienal	10	13
Odor14	Propanal, 3-(methylthio)-	7	3
Odor15	2-Heptenal, (*E*)-	5	4
Odor16	(*E*)-2-Undecenal	4	4
Odor17	Pyridine, 2-pentyl-	4	6
Odor18	1-Octen-3-ol	3	3
Odor19	Decanal	2	2
Odor20	Ethanone, 1-(2-pyridinyl)-	0	6
Odor21	Butylated Hydroxytoluene	2	1
Odor22	Benzaldehyde	1	2
Odor23	Hexanethioic acid, S-methyl ester	1	2

Abbreviations: HIMF, high intramuscular fat group; LIMF, low intramuscular fat group; OAV, odor active values.

Table S8. Significantly changed odorants between the low and high intramuscular fat groups (n=8).

**Odorants name**	**OAV**		***P*-value**	**VIP**
	**HIMF**	**LIMF**		
Pentanal	36	28	0.0215	1.82
2-Acetyl-2-thiazoline	154	91	0.0345	1.20
Furan, 2-pentyl-	20	50	0.0126	3.46
Ethanone, 1-(2-pyridinyl)-	0	6	0.0000	7.98
Octanal	33	26	0.0470	1.24

Abbreviations: HIMF, high intramuscular fat group; LIMF, low intramuscular fat group; VIP, variable importance in projection, OAV, odor active values

Table S9. Correlation between the fatty acids and differential odorants.

Correlation coefficient

Label	2-Acetyl-2-thiazoline	Octanal	Ethanone, 1-(2-pyridinyl)-	Furan, 2-pentyl-	Pentanal	C14:1	C15:0	C16:1	C17:0	C18:0	C18:1n9c	C18:2n6c	C21:0	C20:2	C20:3n6	C20:4n6	C20:3n3	C22:0	C20:5n3	C23:0	C24:0	C24:1	C22:6n3	SFA	MUFA	PUFA
2-Acetyl-2-thiazoline	1	0.61176	-0.6	-0.65	0.61471	0.49521	-0.5012	0.62647	-0.42984	-0.64412	0.73824	-0.68235	-0.47717	-0.74224	-0.75812	-0.78056	-0.33838	-0.17192	-0.40296	-0.62377	-0.62972	-0.62622	-0.66126	-0.35988	0.74118	-0.67059
Octanal	0.61176	1	-0.46471	-0.21471	0.71471	0.38762	-0.1137	0.39706	-0.18759	-0.42941	0.51765	-0.41471	-0.43004	-0.5852	-0.58555	-0.58468	-0.34736	-0.37823	-0.30108	-0.58997	-0.46564	-0.44413	-0.35893	-0.34808	0.52059	-0.45
Ethanone, 1-(2-pyridinyl)-	-0.6	-0.46471	1	0.42647	-0.71176	-0.36257	0.47277	-0.53235	0.59823	0.62941	-0.75294	0.62059	0.61267	0.70817	0.72419	0.69514	0.42222	0.52922	0.62345	0.75898	0.7598	0.69876	0.81168	0.47493	-0.69706	0.68235
Furan, 2-pentyl-	-0.65	-0.21471	0.42647	1	-0.39118	-0.64407	0.63585	-0.77941	0.58789	0.71176	-0.64118	0.66471	0.70398	0.74224	0.6062	0.59941	0.71419	0.15249	0.62801	0.59612	0.63859	0.57884	0.48701	0.18289	-0.72941	0.65588
Pentanal	0.61471	0.71471	-0.71176	-0.39118	1	0.56448	-0.4234	0.56471	-0.6647	-0.77059	0.68824	-0.44706	-0.52283	-0.81928	-0.66372	-0.75258	-0.58542	-0.69516	-0.6569	-0.74822	-0.76719	-0.74169	-0.6419	-0.77286	0.68824	-0.50882
C14:1	0.49521	0.38762	-0.36257	-0.64407	0.56448	1	-0.57578	0.86072	-0.72169	-0.75608	0.51732	-0.52469	-0.58893	-0.62659	-0.51959	-0.65387	-0.72102	-0.14983	-0.30327	-0.56818	-0.37926	-0.61054	-0.35748	-0.31042	0.62344	-0.57038
C15:0	-0.5012	-0.1137	0.47277	0.63585	-0.4234	-0.57578	1	-0.80191	0.82125	0.769	-0.59844	0.55206	0.442	0.65489	0.56495	0.5304	0.67631	0.19924	0.54996	0.46032	0.56545	0.62052	0.62501	0.35412	-0.62687	0.58348
C16:1	0.62647	0.39706	-0.53235	-0.77941	0.56471	0.86072	-0.80191	1	-0.78435	-0.81471	0.69412	-0.76765	-0.72165	-0.78817	-0.66372	-0.73491	-0.72766	-0.15697	-0.49116	-0.67601	-0.5632	-0.68099	-0.55254	-0.28614	0.79412	-0.79412
C17:0	-0.42984	-0.18759	0.59823	0.58789	-0.6647	-0.72169	0.82125	-0.78435	1	0.87889	-0.66766	0.55687	0.51627	0.7314	0.60667	0.65681	0.7527	0.47901	0.60865	0.5625	0.66741	0.75762	0.62156	0.6163	-0.70459	0.61448
C18:0	-0.64412	-0.42941	0.62941	0.71176	-0.77059	-0.75608	0.769	-0.81471	0.87889	1	-0.83235	0.62941	0.60678	0.86965	0.84219	0.84683	0.80252	0.52324	0.64322	0.72057	0.8145	0.90454	0.75955	0.69027	-0.85882	0.70882
C18:1n9c	0.73824	0.51765	-0.75294	-0.64118	0.68824	0.51732	-0.59844	0.69412	-0.66766	-0.83235	1	-0.86765	-0.68041	-0.81336	-0.93953	-0.91605	-0.58542	-0.43803	-0.62801	-0.72364	-0.84258	-0.87493	-0.90849	-0.58702	0.97059	-0.89412
C18:2n6c	-0.68235	-0.41471	0.62059	0.66471	-0.44706	-0.52469	0.55206	-0.76765	0.55687	0.62941	-0.86765	1	0.70398	0.67706	0.78319	0.82327	0.41923	0.07475	0.42273	0.58536	0.62233	0.65434	0.69254	0.20944	-0.9	0.97941
C21:0	-0.47717	-0.43004	0.61267	0.70398	-0.52283	-0.58893	0.442	-0.72165	0.51627	0.60678	-0.68041	0.70398	1	0.7923	0.56278	0.56342	0.68075	0.39376	0.59315	0.71086	0.70689	0.69682	0.55634	0.19498	-0.69514	0.6922
C20:2	-0.74224	-0.5852	0.70817	0.74224	-0.81928	-0.62659	0.65489	-0.78817	0.7314	0.86965	-0.81336	0.67706	0.7923	1	0.76078	0.77746	0.72628	0.62653	0.72766	0.79016	0.90097	0.85981	0.7442	0.54532	-0.82076	0.69928
C20:3n6	-0.75812	-0.58555	0.72419	0.6062	-0.66372	-0.51959	0.56495	-0.66372	0.60667	0.84219	-0.93953	0.78319	0.56278	0.76078	1	0.93058	0.53384	0.3711	0.51701	0.73656	0.79022	0.85153	0.83648	0.55769	-0.92331	0.85989
C20:4n6	-0.78056	-0.58468	0.69514	0.59941	-0.75258	-0.65387	0.5304	-0.73491	0.65681	0.84683	-0.91605	0.82327	0.56342	0.77746	0.93058	1	0.48807	0.32189	0.46218	0.72009	0.73724	0.83248	0.79498	0.56869	-0.9352	0.87924
C20:3n3	-0.33838	-0.34736	0.42222	0.71419	-0.58542	-0.72102	0.67631	-0.72766	0.7527	0.80252	-0.58542	0.41923	0.68075	0.72628	0.53384	0.48807	1	0.50076	0.699	0.56626	0.65092	0.64209	0.4784	0.47903	-0.64232	0.46714
C22:0	-0.17192	-0.37823	0.52922	0.15249	-0.69516	-0.14983	0.19924	-0.15697	0.47901	0.52324	-0.43803	0.07475	0.39376	0.62653	0.3711	0.32189	0.50076	1	0.65079	0.51229	0.68975	0.61628	0.51628	0.67322	-0.33936	0.12707
C20:5n3	-0.40296	-0.30108	0.62345	0.62801	-0.6569	-0.30327	0.54996	-0.49116	0.60865	0.64322	-0.62801	0.42273	0.59315	0.72766	0.51701	0.46218	0.699	0.65079	1	0.73475	0.76195	0.60542	0.60598	0.56429	-0.61737	0.43946
C23:0	-0.62377	-0.58997	0.75898	0.59612	-0.74822	-0.56818	0.46032	-0.67601	0.5625	0.72057	-0.72364	0.58536	0.71086	0.79016	0.73656	0.72009	0.56626	0.51229	0.73475	1	0.68261	0.77101	0.63794	0.50388	-0.72671	0.65297
C24:0	-0.62972	-0.46564	0.7598	0.63859	-0.76719	-0.37926	0.56545	-0.5632	0.66741	0.8145	-0.84258	0.62233	0.70689	0.90097	0.79022	0.73724	0.65092	0.68975	0.76195	0.68261	1	0.85714	0.82562	0.6212	-0.79824	0.66076
C24:1	-0.62622	-0.44413	0.69876	0.57884	-0.74169	-0.61054	0.62052	-0.68099	0.75762	0.90454	-0.87493	0.65434	0.69682	0.85981	0.85153	0.83248	0.64209	0.61628	0.60542	0.77101	0.85714	1	0.84259	0.65182	-0.82163	0.71652
C22:6n3	-0.66126	-0.35893	0.81168	0.48701	-0.6419	-0.35748	0.62501	-0.55254	0.62156	0.75955	-0.90849	0.69254	0.55634	0.7442	0.83648	0.79498	0.4784	0.51628	0.60598	0.63794	0.82562	0.84259	1	0.58554	-0.81615	0.72679

Label	2-Acetyl-2-thiazoline	Octanal	Ethanone, 1-(2-pyridinyl)-	Furan, 2-pentyl-	Pentanal	C14:1	C15:0	C16:1	C17:0	C18:0	C18:1n9c	C18:2n6c	C21:0	C20:2	C20:3n6	C20:4n6	C20:3n3	C22:0	C20:5n3	C23:0	C24:0	C24:1	C22:6n3	SFA	MUFA	PUFA
SFA	-0.35988	-0.34808	0.47493	0.18289	-0.77286	-0.31042	0.35412	-0.28614	0.6163	0.69027	-0.58702	0.20944	0.19498	0.54532	0.55769	0.56869	0.47903	0.67322	0.56429	0.50388	0.6212	0.65182	0.58554	1	-0.55752	0.28614
MUFA	0.74118	0.52059	-0.69706	-0.72941	0.68824	0.62344	-0.62687	0.79412	-0.70459	-0.85882	0.97059	-0.9	-0.69514	-0.82076	-0.92331	-0.9352	-0.64232	-0.33936	-0.61737	-0.72671	-0.79824	-0.82163	-0.81615	-0.55752	1	-0.92941
PUFA	-0.67059	-0.45	0.68235	0.65588	-0.50882	-0.57038	0.58348	-0.79412	0.61448	0.70882	-0.89412	0.97941	0.6922	0.69928	0.85989	0.87924	0.46714	0.12707	0.43946	0.65297	0.66076	0.71652	0.72679	0.28614	-0.92941	1

***P*-value**

Label	2-Acetyl-2-thiazoline	Octanal	Ethanone, 1-(2-pyridinyl)-	Furan, 2-pentyl-	Pentanal	C14:1	C15:0	C16:1	C17:0	C18:0	C18:1n9c	C18:2n6c	C21:0	C20:2	C20:3n6	C20:4n6	C20:3n3	C22:0	C20:5n3	C23:0	C24:0	C24:1	C22:6n3	SFA	MUFA	PUFA
2-Acetyl-2-thiazoline	0	0.012	0.014	0.0064	0.011	0.051	0.048	0.0094	0.097	0.0071	0.0011	0.0036	0.062	9.90E-04	6.70E-04	3.60E-04	0.2	0.52	0.12	0.0098	0.0089	0.0095	0.0053	0.17	0.001	0.0045
Octanal	0.012	0	0.07	0.42	0.0019	0.14	0.68	0.13	0.49	0.097	0.04	0.11	0.096	0.017	0.017	0.017	0.19	0.15	0.26	0.016	0.069	0.085	0.17	0.19	0.039	0.08
Ethanone, 1-(2-pyridinyl)-	0.014	0.07	0	0.099	0.002	0.17	0.064	0.034	0.014	0.009	7.60E-04	0.01	0.012	0.0021	0.0015	0.0028	0.1	0.035	0.0099	6.50E-04	6.40E-04	0.0026	1.30E-04	0.063	0.0027	0.0036
Furan, 2-pentyl-	0.0064	0.42	0.099	0	0.13	0.0071	0.0081	3.70E-04	0.017	0.002	0.0074	0.005	0.0023	9.90E-04	0.013	0.014	0.0019	0.57	0.0092	0.015	0.0078	0.019	0.056	0.5	0.0013	0.0058
Pentanal	0.011	0.0019	0.002	0.13	0	0.023	0.1	0.023	0.005	4.80E-04	0.0032	0.083	0.038	1.00E-04	0.0051	7.70E-04	0.017	0.0028	0.0057	8.60E-04	5.20E-04	0.001	0.0073	4.50E-04	0.0032	0.044
C14:1	0.051	0.14	0.17	0.0071	0.023	0	0.02	1.90E-05	0.0016	7.00E-04	0.04	0.037	0.016	0.0094	0.039	0.006	0.0016	0.58	0.25	0.022	0.15	0.012	0.17	0.24	0.0099	0.021
C15:0	0.048	0.68	0.064	0.0081	0.1	0.02	0	1.90E-04	9.60E-05	5.00E-04	0.014	0.027	0.086	0.0059	0.023	0.035	0.004	0.46	0.027	0.073	0.022	0.01	0.0096	0.18	0.0094	0.018
C16:1	0.0094	0.13	0.034	3.70E-04	0.023	1.90E-05	1.90E-04	0	3.20E-04	1.20E-04	0.0029	5.20E-04	0.0016	2.90E-04	0.0051	0.0012	0.0014	0.56	0.053	0.004	0.023	0.0037	0.026	0.28	2.40E-04	2.40E-04
C17:0	0.097	0.49	0.014	0.017	0.005	0.0016	9.60E-05	3.20E-04	0	7.40E-06	0.0047	0.025	0.041	0.0013	0.013	0.0057	7.70E-04	0.06	0.012	0.023	0.0047	6.70E-04	0.01	0.011	0.0023	0.011
C18:0	0.0071	0.097	0.009	0.002	4.80E-04	7.00E-04	5.00E-04	1.20E-04	7.40E-06	0	6.30E-05	0.009	0.013	1.20E-05	4.30E-05	3.50E-05	1.80E-04	0.038	0.0072	0.0016	1.20E-04	1.50E-06	6.40E-04	0.0031	2.00E-05	0.0021
C18:1n9c	0.0011	0.04	7.60E-04	0.0074	0.0032	0.04	0.014	0.0029	0.0047	6.30E-05	0	1.30E-05	0.0037	1.30E-04	6.70E-08	6.30E-07	0.017	0.09	0.0092	0.0015	4.20E-05	9.20E-06	1.10E-06	0.017	4.70E-10	3.00E-06
C18:2n6c	0.0036	0.11	0.01	0.005	0.083	0.037	0.027	5.20E-04	0.025	0.009	1.30E-05	0	0.0023	0.004	3.30E-04	8.90E-05	0.11	0.78	0.1	0.017	0.01	0.006	0.0029	0.44	2.10E-06	4.00E-11
C21:0	0.062	0.096	0.012	0.0023	0.038	0.016	0.086	0.0016	0.041	0.013	0.0037	0.0023	0	2.50E-04	0.023	0.023	0.0037	0.13	0.015	0.002	0.0022	0.0027	0.025	0.47	0.0028	0.003
C20:2	9.90E-04	0.017	0.0021	9.90E-04	1.00E-04	0.0094	0.0059	2.90E-04	0.0013	1.20E-05	1.30E-04	0.004	2.50E-04	0	6.20E-04	3.90E-04	0.0014	0.0094	0.0014	2.70E-04	1.90E-06	2.00E-05	9.50E-04	0.029	9.80E-05	0.0026
C20:3n6	6.70E-04	0.017	0.0015	0.013	0.0051	0.039	0.023	0.0051	0.013	4.30E-05	6.70E-08	3.30E-04	0.023	6.20E-04	0	1.70E-07	0.033	0.16	0.04	0.0011	2.70E-04	2.90E-05	5.40E-05	0.025	3.40E-07	1.90E-05
C20:4n6	3.60E-04	0.017	0.0028	0.014	7.70E-04	0.006	0.035	0.0012	0.0057	3.50E-05	6.30E-07	8.90E-05	0.023	3.90E-04	1.70E-07	0	0.055	0.22	0.071	0.0017	0.0011	6.30E-05	2.30E-04	0.022	1.10E-07	7.30E-06
C20:3n3	0.2	0.19	0.1	0.0019	0.017	0.0016	0.004	0.0014	7.70E-04	1.80E-04	0.017	0.11	0.0037	0.0014	0.033	0.055	0	0.048	0.0026	0.022	0.0063	0.0073	0.061	0.06	0.0073	0.068
C22:0	0.52	0.15	0.035	0.57	0.0028	0.58	0.46	0.56	0.06	0.038	0.09	0.78	0.13	0.0094	0.16	0.22	0.048	0	0.0063	0.042	0.0031	0.011	0.041	0.0043	0.2	0.64
C20:5n3	0.12	0.26	0.0099	0.0092	0.0057	0.25	0.027	0.053	0.012	0.0072	0.0092	0.1	0.015	0.0014	0.04	0.071	0.0026	0.0063	0	0.0012	6.00E-04	0.013	0.013	0.023	0.011	0.089
C23:0	0.0098	0.016	6.50E-04	0.015	8.60E-04	0.022	0.073	0.004	0.023	0.0016	0.0015	0.017	0.002	2.70E-04	0.0011	0.0017	0.022	0.042	0.0012	0	0.0036	4.70E-04	0.0078	0.047	0.0014	0.0061

C24:0	0.0089	0.069	6.40E-04	0.0078	5.20E-04	0.15	0.022	0.023	0.0047	1.20E-04	4.20E-05	0.01	0.0022	1.90E-06	2.70E-04	0.0011	0.0063	0.0031	6.00E-04	0.0036	0	2.20E-05	8.20E-05	0.01	2.10E-04	0.0053
C24:1	0.0095	0.085	0.0026	0.019	0.001	0.012	0.01	0.0037	6.70E-04	1.50E-06	9.20E-06	0.006	0.0027	2.00E-05	2.90E-05	6.30E-05	0.0073	0.011	0.013	4.70E-04	2.20E-05	0	4.20E-05	0.0062	9.50E-05	0.0018
C22:6n3	0.0053	0.17	1.30E-04	0.056	0.0073	0.17	0.0096	0.026	0.01	6.40E-04	1.10E-06	0.0029	0.025	9.50E-04	5.40E-05	2.30E-04	0.061	0.041	0.013	0.0078	8.20E-05	4.20E-05	0	0.017	1.20E-04	0.0014
SFA	0.17	0.19	0.063	0.5	4.50E-04	0.24	0.18	0.28	0.011	0.0031	0.017	0.44	0.47	0.029	0.025	0.022	0.06	0.0043	0.023	0.047	0.01	0.0062	0.017	0	0.025	0.28
MUFA	0.001	0.039	0.0027	0.0013	0.0032	0.0099	0.0094	2.40E-04	0.0023	2.00E-05	4.70E-10	2.10E-06	0.0028	9.80E-05	3.40E-07	1.10E-07	0.0073	0.2	0.011	0.0014	2.10E-04	9.50E-05	1.20E-04	0.025	0	1.90E-07
PUFA	0.0045	0.08	0.0036	0.0058	0.044	0.021	0.018	2.40E-04	0.011	0.0021	3.00E-06	4.00E-11	0.003	0.0026	1.90E-05	7.30E-06	0.068	0.64	0.089	0.0061	0.0053	0.0018	0.0014	0.28	1.90E-07	0

Table S10. Correlation between the differential lipids and differential odorants.

Correlation coefficient

Label	2-Acetyl-2-thiazoline	Octanal	Ethanone, 1-(2-pyridinyl)-	Furan, 2-pentyl-	Pentanal	DG 30:1	TG 10:0_16:0_16:1	TG 8:0_16:1_18:1	TG 10:0_16:0_18:2	TG 10:0_16:1_18:2	TG 16:0_14:1_16:1	TG 14:1_16:1_16:1	TG 14:0_16:1_18:2	TG 16:0_16:1_18:2	TG 16:0_17:1_18:1	CL 16:0_28:0_28:0_28:0	PC 15:4_24:6	PC 16:1_16:1	PC 15:0_18:2	PC 17:0_18:2	PC O-32:3	PC O-15:2_22:6	PE 6:0_42:1	PE 42:0_6:0	PE O-16:0_28:0;O1	PE O-18:0_28:0;O1	PS 40:5	LPG O-24:6	NAGlySer 20:0;O(FA 28:1)	Cer 16:1;O3/38:6(2OH)	Cer 18:2;O3/38:6(2OH)	Cer 20:2;O3/38:6(2OH)	Cer 18:1;O2/27:1	SM 12:1;O2/35:0
2-Acetyl-2-thiazoline	1	0.61176	-0.6	-0.65	0.61471	0.58235	0.53824	0.56471	0.6	0.62353	0.57647	0.60294	0.63824	0.72941	0.75588	0.64706	-0.58529	-0.63235	-0.69412	-0.62941	-0.69118	-0.69118	0.61765	0.61471	0.69706	0.66471	-0.81471	0.80294	0.77353	0.62647	0.58235	0.72941	-0.62353	0.79412
Octanal	0.61176	1	-0.46471	-0.21471	0.71471	0.61176	0.30294	0.30882	0.34706	0.34118	0.33824	0.3	0.32353	0.43824	0.51765	0.66176	-0.53824	-0.54706	-0.61471	-0.46176	-0.44412	-0.47941	0.49412	0.59706	0.45294	0.44412	-0.52353	0.59706	0.57059	0.52647	0.49412	0.42059	-0.80294	0.52941
Ethanone, 1-(2-pyridinyl)-	-0.6	-0.46471	1	0.42647	-0.71176	-0.58235	-0.66176	-0.60588	-0.63824	-0.65588	-0.66471	-0.66176	-0.58824	-0.65294	-0.73235	-0.62353	0.60294	0.62059	0.64412	0.71765	0.7	0.80588	-0.58235	-0.62353	-0.68529	-0.65	0.69118	-0.67353	-0.70294	-0.70882	-0.69118	-0.7	0.69412	-0.72647
Furan, 2-pentyl-	-0.65	-0.21471	0.42647	1	-0.39118	-0.45882	-0.71765	-0.70294	-0.70588	-0.71176	-0.69412	-0.68824	-0.65294	-0.59412	-0.64118	-0.39706	0.68529	0.68824	0.62353	0.55	0.63824	0.51176	-0.57647	-0.55294	-0.73235	-0.71765	0.61765	-0.68235	-0.65294	-0.58824	-0.58824	-0.76765	0.37647	-0.64118
Pentanal	0.61471	0.71471	-0.71176	-0.39118	1	0.69412	0.69118	0.73235	0.74706	0.77353	0.71765	0.75	0.70294	0.74118	0.83824	0.89118	-0.76176	-0.8	-0.85	-0.87353	-0.68235	-0.77353	0.82353	0.81765	0.74706	0.75294	-0.78824	0.77647	0.75	0.67059	0.70294	0.70588	-0.84412	0.83824
DG 30:1	0.58235	0.61176	-0.58235	-0.45882	0.69412	1	0.78824	0.78235	0.79118	0.78235	0.80882	0.74412	0.74706	0.78529	0.83235	0.81471	-0.74412	-0.58529	-0.69118	-0.60294	-0.70588	-0.72353	0.76176	0.78235	0.73824	0.78529	-0.62353	0.82941	0.85882	0.7	0.79412	0.69706	-0.69118	0.81765
TG 10:0_16:0_16:1	0.53824	0.30294	-0.66176	-0.71765	0.69118	0.78824	1	0.97059	0.96765	0.93529	0.98235	0.92059	0.85882	0.79412	0.83235	0.68235	-0.78235	-0.72059	-0.73235	-0.75	-0.79706	-0.70882	0.81471	0.77941	0.87647	0.9	-0.67059	0.80588	0.87647	0.81765	0.88235	0.85882	-0.63529	0.82647
TG 8:0_16:1_18:1	0.56471	0.30882	-0.60588	-0.70294	0.73235	0.78235	0.97059	1	0.99706	0.97647	0.97647	0.96765	0.93529	0.85294	0.87647	0.75588	-0.80294	-0.72647	-0.76176	-0.75294	-0.74412	-0.71176	0.85294	0.79412	0.86765	0.89706	-0.71765	0.84412	0.86176	0.74706	0.82941	0.83235	-0.6	0.87647
TG 10:0_16:0_18:2	0.6	0.34706	-0.63824	-0.70588	0.74706	0.79118	0.96765	0.99706	1	0.97941	0.97941	0.97353	0.94706	0.87353	0.89118	0.77059	-0.82059	-0.74412	-0.78235	-0.76471	-0.75294	-0.73529	0.85882	0.80588	0.88235	0.90588	-0.74706	0.86471	0.87941	0.76176	0.83824	0.84706	-0.62647	0.89118
TG 10:0_16:1_18:2	0.62353	0.34118	-0.65588	-0.71176	0.77353	0.78235	0.93529	0.97647	0.97941	1	0.95588	0.98824	0.96176	0.90882	0.93824	0.80588	-0.85882	-0.78235	-0.80294	-0.79706	-0.78235	-0.78824	0.89706	0.81765	0.89118	0.91471	-0.81176	0.85882	0.86471	0.70294	0.79706	0.86765	-0.62647	0.92941
TG 16:0_14:1_16:1	0.57647	0.33824	-0.66471	-0.69412	0.71765	0.80882	0.98235	0.97647	0.97941	0.95588	1	0.95588	0.91471	0.87059	0.86176	0.74118	-0.77941	-0.74706	-0.76765	-0.75294	-0.79706	-0.74118	0.86471	0.82059	0.89706	0.91765	-0.72353	0.83824	0.89412	0.78824	0.87059	0.85882	-0.65882	0.87059
TG 14:1_16:1_16:1	0.60294	0.3	-0.66176	-0.68824	0.75	0.74412	0.92059	0.96765	0.97353	0.98824	0.95588	1	0.97941	0.92941	0.92353	0.79706	-0.82647	-0.77353	-0.8	-0.78529	-0.73529	-0.77647	0.87647	0.80294	0.87353	0.88824	-0.81765	0.83235	0.82941	0.65294	0.74706	0.83235	-0.58824	0.90882
TG 14:0_16:1_18:2	0.63824	0.32353	-0.58824	-0.65294	0.70294	0.74706	0.85882	0.93529	0.94706	0.96176	0.91471	0.97941	1	0.95	0.91471	0.80294	-0.80294	-0.72059	-0.76471	-0.70294	-0.68235	-0.73529	0.85588	0.77353	0.83529	0.85	-0.82353	0.82941	0.81471	0.58824	0.68824	0.78529	-0.54706	0.90294
TG 16:0_16:1_18:2	0.72941	0.43824	-0.65294	-0.59412	0.74118	0.78529	0.79412	0.85294	0.87353	0.90882	0.87059	0.92941	0.95	1	0.93824	0.87059	-0.77647	-0.74412	-0.81471	-0.76471	-0.69412	-0.85588	0.89118	0.82941	0.85588	0.86765	-0.87941	0.85882	0.81765	0.57059	0.67353	0.80882	-0.65294	0.93824
TG 16:0_17:1_1	0.75588	0.51765	-0.73235	-0.64118	0.83824	0.83235	0.83235	0.87647	0.89118	0.93824	0.86176	0.92353	0.91471	0.93824	1	0.90588	-0.87941	-0.80294	-0.85882	-0.83824	-0.78235	-0.87353	0.87941	0.84412	0.87059	0.87941	-0.91176	0.87941	0.86471	0.6588	0.72941	0.85	-0.68824	0.96765

8:1																													2					
CL 16:0_28:0_28:0_28:0	0.64706	0.66176	-0.62353	-0.39706	0.89118	0.81471	0.68235	0.75588	0.77059	0.80588	0.74118	0.79706	0.80294	0.87059	0.90588	1	-0.78529	-0.78235	-0.87353	-0.78824	-0.65	-0.79706	0.87647	0.91471	0.79118	0.80588	-0.81765	0.8	0.77941	0.58824	0.66471	0.71765	-0.75882	0.90294
PC 15:4_24:6	-0.58529	-0.53824	0.60294	0.68529	-0.76176	-0.74412	-0.78235	-0.80294	-0.82059	-0.85882	-0.77941	-0.82647	-0.80294	-0.77647	-0.87941	-0.78529	1	0.86471	0.87353	0.77941	0.67941	0.72353	-0.77941	-0.8	-0.81176	-0.81765	0.79118	-0.77647	-0.76765	-0.62647	-0.67941	-0.79706	0.64118	-0.8
PC 16:1_16:1	-0.63235	-0.54706	0.62059	0.68824	-0.8	-0.58529	-0.72059	-0.72647	-0.74412	-0.78235	-0.74706	-0.77353	-0.72059	-0.74412	-0.80294	-0.78235	0.86471	1	0.94118	0.83824	0.76176	0.67941	-0.81765	-0.88824	-0.87059	-0.83235	0.84118	-0.69412	-0.76471	-0.67353	-0.68235	-0.82941	0.71176	-0.78529
PC 15:0_18:2	-0.69412	-0.61471	0.64412	0.62353	-0.85	-0.69118	-0.73235	-0.76176	-0.78235	-0.80294	-0.76765	-0.8	-0.76471	-0.81471	-0.85882	-0.87353	0.87353	0.94118	1	0.88824	0.67059	0.76471	-0.80294	-0.89706	-0.83235	-0.81765	0.82647	-0.81765	-0.78235	-0.66176	-0.67647	-0.77941	0.71471	-0.83235
PC 17:0_18:2	-0.62941	-0.46176	0.71765	0.55	-0.87353	-0.60294	-0.75	-0.75294	-0.76471	-0.79706	-0.75294	-0.78529	-0.70294	-0.76471	-0.83824	-0.78824	0.77941	0.83824	0.88824	1	0.67059	0.87059	-0.79706	-0.78824	-0.82059	-0.81765	0.81765	-0.77647	-0.71176	-0.65	-0.67647	-0.80882	0.71471	-0.81765
PC O-32:3	-0.69118	-0.44412	0.7	0.63824	-0.68235	-0.70588	-0.79706	-0.74412	-0.75294	-0.78235	-0.79706	-0.73529	-0.68235	-0.69412	-0.78235	-0.65	0.67941	0.76176	0.67059	0.67059	1	0.65882	-0.79118	-0.77059	-0.86471	-0.84118	0.76176	-0.68235	-0.91765	-0.85294	-0.86765	-0.88529	0.71765	-0.82059
PC O-15:2_22:6	-0.69118	-0.47941	0.80588	0.51176	-0.77353	-0.72353	-0.70882	-0.71176	-0.73529	-0.78824	-0.74118	-0.77647	-0.73529	-0.85588	-0.87353	-0.79706	0.72353	0.67941	0.76471	0.87059	0.65882	1	-0.80882	-0.75588	-0.81176	-0.82941	0.80588	-0.84706	-0.72941	-0.60882	-0.68824	-0.82059	0.74118	-0.88235
PE 6:0_42:1	0.61765	0.49412	-0.58235	-0.57647	0.82353	0.76176	0.81471	0.85294	0.85882	0.89706	0.86471	0.87647	0.85588	0.89118	0.87941	0.87647	-0.77941	-0.81765	-0.80294	-0.79706	-0.79118	-0.80882	1	0.92353	0.92647	0.95	-0.82059	0.79412	0.83529	0.68235	0.79706	0.88824	-0.80588	0.92647
PE 42:0_6:0	0.61471	0.59706	-0.62353	-0.55294	0.81765	0.78235	0.77941	0.79412	0.80588	0.81765	0.82059	0.80294	0.77353	0.82941	0.84412	0.91471	-0.8	-0.88824	-0.89706	-0.78824	-0.77059	-0.75588	0.92353	1	0.91765	0.91765	-0.77647	0.77059	0.85	0.74706	0.80882	0.85294	-0.81765	0.87647
PE O-16:0_28:0;O1	0.69706	0.45294	-0.68529	-0.73235	0.74706	0.73824	0.87647	0.86765	0.88235	0.89118	0.89706	0.87353	0.83529	0.85588	0.87059	0.79118	-0.81176	-0.87059	-0.83235	-0.82059	-0.86471	-0.81176	0.92647	0.91765	1	0.98824	-0.84706	0.81471	0.90294	0.82059	0.87353	0.97941	-0.77059	0.90882
PE O-18:0_28:0;O1	0.66471	0.44412	-0.65	-0.71765	0.75294	0.78529	0.9	0.89706	0.90588	0.91471	0.91765	0.88824	0.85	0.86765	0.87941	0.80588	-0.81765	-0.83235	-0.81765	-0.81765	-0.84118	-0.82941	0.95	0.91765	0.98824	1	-0.80588	0.84412	0.9	0.80882	0.88824	0.97059	-0.77647	0.92059
PS 40:5	-0.81471	-0.52353	0.69118	0.61765	-0.78824	-0.62353	-0.67059	-0.71765	-0.74706	-0.81176	-0.72353	-0.81765	-0.82353	-0.87941	-0.91176	-0.81765	0.79118	0.84118	0.82647	0.81765	0.76176	0.80588	-0.82059	-0.77647	-0.84706	-0.80588	1	-0.75	-0.77647	-0.56765	-0.59412	-0.82647	0.66176	-0.88824
LPG O-24:6	0.80294	0.59706	-0.67353	-0.68235	0.77647	0.82941	0.80588	0.84412	0.86471	0.85882	0.83824	0.83235	0.82941	0.85882	0.87941	0.8	-0.77647	-0.69412	-0.81765	-0.77647	-0.68235	-0.84706	0.79412	0.77059	0.81471	0.84412	-0.75	1	0.86765	0.74706	0.79706	0.80588	-0.81765	0.91176
NAGlySer 20:0;O(FA 28:1)	0.77353	0.57059	-0.70294	-0.65294	0.75	0.85882	0.87647	0.86176	0.87941	0.86471	0.89412	0.82941	0.81471	0.81765	0.86471	0.77941	-0.76765	-0.76471	-0.78235	-0.71176	-0.91765	-0.72941	0.83529	0.85	0.90294	0.9	-0.77647	0.86765	1	0.90882	0.93235	0.89118	-0.76765	0.90294
Cer 16:1;O3/38:6(2OH)	0.62647	0.52647	-0.70882	-0.58824	0.67059	0.7	0.81765	0.74706	0.76176	0.70294	0.78824	0.65294	0.58824	0.57059	0.65882	0.58824	-0.62647	-0.67353	-0.66176	-0.65	-0.85294	-0.60882	0.68235	0.74706	0.82059	0.80882	-0.56765	0.74706	0.90882	1	0.96765	0.83235	-0.77353	0.72941
Cer 18:2;O3/38:6(2OH)	0.58235	0.49412	-0.69118	-0.58824	0.70294	0.79412	0.88235	0.82941	0.83824	0.79706	0.87059	0.74706	0.68824	0.67353	0.72941	0.66471	-0.67941	-0.68235	-0.67647	-0.67647	-0.86765	-0.68824	0.79706	0.80882	0.87353	0.88824	-0.59412	0.79706	0.93235	0.96765	1	0.87353	-0.8	0.80294
Cer 20:2;O3/38:6(2OH)	0.72941	0.42059	-0.7	-0.76765	0.70588	0.69706	0.85882	0.83235	0.84706	0.86765	0.85882	0.83235	0.78529	0.80882	0.85	0.71765	-0.79706	-0.82941	-0.77941	-0.80882	-0.88529	-0.82059	0.88824	0.85294	0.97941	0.97059	-0.82647	0.80588	0.89118	0.83235	0.87353	1	-0.76176	0.89118
Cer 18:1;O2/27:	-0.62353	-0.80294	0.69412	0.37647	-0.84412	-0.69118	-0.63529	-0.6	-0.62647	-0.62647	-0.65882	-0.58824	-0.54706	-0.65294	-0.68824	-0.75882	0.64118	0.71176	0.71471	0.71471	0.71765	0.74118	-0.80588	-0.81765	-0.77059	-0.77647	0.66176	-0.71765	-0.76765	-0.7735	-0.8	-0.76176	1	-0.75294

1																														3				
SM 12:1;O2/35:0	0.79412	0.52941	-0.72647	-0.64118	0.83824	0.81765	0.82647	0.87647	0.89118	0.92941	0.87059	0.90882	0.90294	0.93824	0.96765	0.90294	-0.8	-0.78529	-0.83235	-0.81765	-0.82059	-0.88235	0.92647	0.87647	0.90882	0.92059	-0.88824	0.91176	0.90294	0.72941	0.80294	0.89118	-0.75294	1

***P*-value**

Label	2-Acetyl-2-thiazoline	Octanal	Ethanone, 1-(2-pyridinyl)-	Furan, 2-pentyl-	Pentanal	DG 30:1	TG 10:0_16:0_16:1	TG 8:0_16:1_18:1	TG 10:0_16:0_18:2	TG 10:0_16:1_18:2	TG 16:0_14:1_16:1	TG 14:1_16:1_16:1	TG 14:0_16:1_18:2	TG 16:0_16:1_18:2	TG 16:0_17:1_18:1	CL 16:0_28:0_28:0_28:0	PC 15:4_24:6	PC 16:1_16:1	PC 15:0_18:2	PC 17:0_18:2	PC O-32:3	PC O-15:2_22:6	PE 6:0_42:1	PE 42:0_6:0	PE O-16:0_28:0;O1	PE O-18:0_28:0;O1	PS 40:5	LPG O-24:6	NAGlySer 20:0;O(FA 28:1)	Cer 16:1;O3/38:6(2OH)	Cer 18:2;O3/38:6(2OH)	Cer 20:2;O3/38:6(2OH)	Cer 18:1;O2/27:1	SM 12:1;O2/35:0
2-Acetyl-2-thiazoline	0	0.012	0.014	0.0064	0.011	0.018	0.031	0.023	0.014	0.0099	0.019	0.013	0.0078	0.0013	7.10E-04	0.0067	0.017	0.0086	0.0029	0.009	0.003	0.003	0.011	0.011	0.0027	0.005	1.20E-04	1.80E-04	4.40E-04	0.0094	0.018	0.0013	0.0099	2.40E-04
Octanal	0.012	0	0.07	0.42	0.0019	0.012	0.25	0.24	0.19	0.2	0.2	0.26	0.22	0.09	0.04	0.0052	0.031	0.028	0.011	0.072	0.085	0.06	0.052	0.015	0.078	0.085	0.037	0.015	0.021	0.036	0.052	0.1	1.80E-04	0.035
Ethanone, 1-(2-pyridinyl)-	0.014	0.07	0	0.099	0.002	0.018	0.0052	0.013	0.0078	0.0058	0.005	0.0052	0.017	0.0061	0.0013	0.0099	0.013	0.01	0.0071	0.0017	0.0025	1.60E-04	0.018	0.0099	0.0034	0.0064	0.003	0.0042	0.0024	0.0021	0.003	0.0025	0.0029	0.0014
Furan, 2-pentyl-	0.0064	0.42	0.099	0	0.13	0.074	0.0017	0.0024	0.0022	0.002	0.0029	0.0032	0.0061	0.015	0.0074	0.13	0.0034	0.0032	0.0099	0.027	0.0078	0.043	0.019	0.026	0.0013	0.0017	0.011	0.0036	0.0061	0.017	0.017	5.20E-04	0.15	0.0074
Pentanal	0.011	0.0019	0.002	0.13	0	0.0029	0.003	0.0013	8.80E-04	4.40E-04	0.0017	8.20E-04	0.0024	0.001	5.00E-05	3.60E-06	6.10E-04	2.00E-04	3.10E-05	9.90E-06	0.0036	4.40E-04	8.80E-05	1.10E-04	8.80E-04	7.60E-04	2.90E-04	4.00E-04	8.20E-04	0.0045	0.0024	0.0022	3.90E-05	5.00E-05
DG 30:1	0.018	0.012	0.018	0.074	0.0029	0	2.90E-04	3.40E-04	2.60E-04	3.40E-04	1.50E-04	9.50E-04	8.80E-04	3.10E-04	6.30E-05	1.20E-04	9.50E-04	0.017	0.003	0.013	0.0022	0.0015	6.10E-04	3.40E-04	0.0011	3.10E-04	0.0099	7.10E-05	2.00E-05	0.0025	2.40E-04	0.0027	0.003	1.10E-04
TG 10:0_16:0_16:1	0.031	0.25	0.0052	0.0017	0.003	2.90E-04	0	4.70E-10	9.10E-10	1.10E-07	1.40E-11	4.30E-07	2.00E-05	2.40E-04	6.30E-05	0.0036	3.40E-04	0.0016	0.0013	8.20E-04	2.20E-04	0.0021	1.20E-04	3.70E-04	8.40E-06	2.10E-06	0.0045	1.60E-04	8.40E-06	1.10E-04	6.10E-06	2.00E-05	0.0082	7.90E-05
TG 8:0_16:1_18:1	0.023	0.24	0.013	0.0024	0.0013	3.40E-04	4.70E-10	0	5.10E-17	1.00E-10	1.00E-10	9.10E-10	1.10E-07	2.70E-05	8.40E-06	7.10E-04	1.80E-04	0.0014	6.10E-04	7.60E-04	9.50E-04	0.002	2.70E-05	2.40E-04	1.30E-05	2.50E-06	0.0017	3.90E-05	1.80E-05	8.80E-04	7.10E-05	6.30E-05	0.014	8.40E-06
TG 10:0_16:0_18:2	0.014	0.19	0.0078	0.0022	8.80E-04	2.60E-04	9.10E-10	5.10E-17	0	4.00E-11	4.00E-11	2.30E-10	2.70E-08	9.90E-06	3.60E-06	4.80E-04	9.80E-05	9.50E-04	3.40E-04	5.60E-04	7.60E-04	0.0012	2.00E-05	1.60E-04	6.10E-06	1.40E-06	8.80E-04	1.50E-05	7.20E-06	6.10E-04	5.00E-05	3.50E-05	0.0094	3.60E-06
TG 10:0_16:1_18:2	0.0099	0.2	0.0058	0.002	4.40E-04	3.40E-04	1.10E-07	1.00E-10	4.00E-11	0	7.80E-09	8.10E-13	2.90E-09	1.10E-06	7.80E-08	1.60E-04	2.00E-05	3.40E-04	1.80E-04	2.20E-04	3.40E-04	2.90E-04	2.50E-06	1.10E-04	3.60E-06	7.00E-07	1.30E-04	2.00E-05	1.50E-05	0.0024	2.20E-04	1.30E-05	0.0094	1.90E-07
TG 16:0_14:1_16:1	0.019	0.2	0.005	0.0029	0.0017	1.50E-04	1.40E-11	1.00E-10	4.00E-11	7.80E-09	0	7.80E-09	7.00E-07	1.10E-05	1.80E-05	0.001	3.70E-04	8.80E-04	5.20E-04	7.60E-04	2.20E-04	0.001	1.50E-05	9.80E-05	2.50E-06	5.50E-07	0.0015	5.00E-05	3.00E-06	2.90E-04	1.10E-05	2.00E-05	0.0055	1.10E-05
TG 14:1_16:1_16:1	0.013	0.26	0.0052	0.0032	8.20E-04	9.50E-04	4.30E-07	9.10E-10	2.30E-10	8.10E-13	7.80E-09	0	4.00E-11	1.90E-07	3.30E-07	2.20E-04	7.90E-05	4.40E-04	2.00E-04	3.10E-04	0.0012	4.00E-04	8.40E-06	1.80E-04	9.90E-06	4.30E-06	1.10E-04	6.30E-05	7.10E-05	0.0061	8.80E-04	6.30E-05	0.017	1.10E-06
TG 14:0_16:1_18:2	0.0078	0.22	0.017	0.0061	0.0024	8.80E-04	2.00E-05	1.10E-07	2.70E-08	2.90E-09	7.00E-07	4.00E-11	0	1.80E-08	7.00E-07	1.80E-04	1.80E-04	0.0016	5.60E-04	0.0024	0.0036	0.0012	2.30E-05	4.40E-04	5.60E-05	3.10E-05	8.80E-05	7.10E-05	1.20E-04	0.017	0.0032	3.10E-04	0.028	1.70E-06
TG 16:0_16:1_18:2	0.0013	0.09	0.0061	0.015	0.001	3.10E-04	2.40E-04	2.70E-05	9.90E-06	1.10E-06	1.10E-05	1.90E-07	1.80E-08	0	7.80E-08	1.10E-05	4.00E-04	9.50E-04	1.20E-04	5.60E-04	0.0029	2.30E-05	3.60E-06	7.10E-05	2.30E-05	1.30E-05	7.20E-06	2.00E-05	1.10E-04	0.021	0.0042	1.50E-04	0.0061	7.80E-08
TG 16:0_17:1_1	7.10E-04	0.04	0.0013	0.0074	5.00E-05	6.30E-05	6.30E-05	8.40E-06	3.60E-06	7.80E-08	1.80E-05	3.30E-07	7.00E-07	7.80E-08	0	1.40E-06	7.20E-06	1.80E-04	2.00E-05	5.00E-05	3.40E-04	9.90E-06	7.20E-06	3.90E-05	1.10E-05	7.20E-06	8.80E-07	7.20E-06	1.50E-05	0.0055	0.0013	3.10E-05	0.0032	9.10E-10

8:1																																		
CL 16:0_28:0_28:0_28:0	0.0067	0.0052	0.0099	0.13	3.60E-06	1.20E-04	0.0036	7.10E-04	4.80E-04	1.60E-04	0.001	2.20E-04	1.80E-04	1.10E-05	1.40E-06	0	3.10E-04	3.40E-04	9.90E-06	2.90E-04	0.0064	2.20E-04	8.40E-06	7.00E-07	2.60E-04	1.60E-04	1.10E-04	2.00E-04	3.70E-04	0.017	0.005	0.0017	6.50E-04	1.70E-06
PC 15:4_24:6	0.017	0.031	0.013	0.0034	6.10E-04	9.50E-04	3.40E-04	1.80E-04	9.80E-05	2.00E-05	3.70E-04	7.90E-05	1.80E-04	4.00E-04	7.20E-06	3.10E-04	0	1.50E-05	9.90E-06	3.70E-04	0.0038	0.0015	3.70E-04	2.00E-04	1.30E-04	1.10E-04	2.60E-04	4.00E-04	5.20E-04	0.0094	0.0038	2.20E-04	0.0074	2.00E-04
PC 16:1_16:1	0.0086	0.028	0.01	0.0032	2.00E-04	0.017	0.0016	0.0014	9.50E-04	3.40E-04	8.80E-04	4.40E-04	0.0016	9.50E-04	1.80E-04	3.40E-04	1.50E-05	0	5.60E-08	5.00E-05	6.10E-04	0.0038	1.10E-04	4.30E-06	1.10E-05	6.30E-05	4.40E-05	0.0029	5.60E-04	0.0042	0.0036	7.10E-05	0.002	3.10E-04
PC 15:0_18:2	0.0029	0.011	0.0071	0.0099	3.10E-05	0.003	0.0013	6.10E-04	3.40E-04	1.80E-04	5.20E-04	2.00E-04	5.60E-04	1.20E-04	2.00E-05	9.90E-06	9.90E-06	5.60E-08	0	4.30E-06	0.0045	5.60E-04	1.80E-04	2.50E-06	6.30E-05	1.10E-04	7.90E-05	1.10E-04	3.40E-04	0.0052	0.004	3.70E-04	0.0019	6.30E-05
PC 17:0_18:2	0.009	0.072	0.0017	0.027	9.90E-06	0.013	8.20E-04	7.60E-04	5.60E-04	2.20E-04	7.60E-04	3.10E-04	0.0024	5.60E-04	5.00E-05	2.90E-04	3.70E-04	5.00E-05	4.30E-06	0	0.0045	1.10E-05	2.20E-04	2.90E-04	9.80E-05	1.10E-04	1.10E-04	4.00E-04	0.002	0.0064	0.004	1.50E-04	0.0019	1.10E-04
PC O-32:3	0.003	0.085	0.0025	0.0078	0.0036	0.0022	2.20E-04	9.50E-04	7.60E-04	3.40E-04	2.20E-04	0.0012	0.0036	0.0029	3.40E-04	0.0064	0.0038	6.10E-04	0.0045	0.0045	0	0.0055	2.60E-04	4.80E-04	1.50E-05	4.40E-05	6.10E-04	0.0036	5.50E-07	2.70E-05	1.30E-05	5.10E-06	0.0017	9.80E-05
PC O-15:2_22:6	0.003	0.06	1.60E-04	0.043	4.40E-04	0.0015	0.0021	0.002	0.0012	2.90E-04	0.001	4.00E-04	0.0012	2.30E-05	9.90E-06	2.20E-04	0.0015	0.0038	5.60E-04	1.10E-05	0.0055	0	1.50E-04	7.10E-04	1.30E-04	7.10E-05	1.60E-04	3.50E-05	0.0013	0.012	0.0032	9.80E-05	0.001	6.10E-06
PE 6:0_42:1	0.011	0.052	0.018	0.019	8.80E-05	6.10E-04	1.20E-04	2.70E-05	2.00E-05	2.50E-06	1.50E-05	8.40E-06	2.30E-05	3.60E-06	7.20E-06	8.40E-06	3.70E-04	1.10E-04	1.80E-04	2.20E-04	2.60E-04	1.50E-04	0	3.30E-07	2.60E-07	1.80E-08	9.80E-05	2.40E-04	5.60E-05	0.0036	2.20E-04	4.30E-06	1.60E-04	2.60E-07
PE 42:0_6:0	0.011	0.015	0.0099	0.026	1.10E-04	3.40E-04	3.70E-04	2.40E-04	1.60E-04	1.10E-04	9.80E-05	1.80E-04	4.40E-04	7.10E-05	3.90E-05	7.00E-07	2.00E-04	4.30E-06	2.50E-06	2.90E-04	4.80E-04	7.10E-04	3.30E-07	0	5.50E-07	5.50E-07	4.00E-04	4.80E-04	3.10E-05	8.80E-04	1.50E-04	2.70E-05	1.10E-04	8.40E-06
PE O-16:0_28:0;O1	0.0027	0.078	0.0034	0.0013	8.80E-04	0.0011	8.40E-06	1.30E-05	6.10E-06	3.60E-06	2.50E-06	9.90E-06	5.60E-05	2.30E-05	1.10E-05	2.60E-04	1.30E-04	1.10E-05	6.30E-05	9.80E-05	1.50E-05	1.30E-04	2.60E-07	5.50E-07	0	8.10E-13	3.50E-05	1.20E-04	1.70E-06	9.80E-05	9.90E-06	4.00E-11	4.80E-04	1.10E-06
PE O-18:0_28:0;O1	0.005	0.085	0.0064	0.0017	7.60E-04	3.10E-04	2.10E-06	2.50E-06	1.40E-06	7.00E-07	5.50E-07	4.30E-06	3.10E-05	1.30E-05	7.20E-06	1.60E-04	1.10E-04	6.30E-05	1.10E-04	1.10E-04	4.40E-05	7.10E-05	1.80E-08	5.50E-07	8.10E-13	0	1.60E-04	3.90E-05	2.10E-06	1.50E-04	4.30E-06	4.70E-10	4.00E-04	4.30E-07
PS 40:5	1.20E-04	0.037	0.003	0.011	2.90E-04	0.0099	0.0045	0.0017	8.80E-04	1.30E-04	0.0015	1.10E-04	8.80E-05	7.20E-06	8.80E-07	1.10E-04	2.60E-04	4.40E-05	7.90E-05	1.10E-04	6.10E-04	1.60E-04	9.80E-05	4.00E-04	3.50E-05	1.60E-04	0	8.20E-04	4.00E-04	0.022	0.015	7.90E-05	0.0052	4.30E-06
LPG O-24:6	1.80E-04	0.015	0.0042	0.0036	4.00E-04	7.10E-05	1.60E-04	3.90E-05	1.50E-05	2.00E-05	5.00E-05	6.30E-05	7.10E-05	2.00E-05	7.20E-06	2.00E-04	4.00E-04	0.0029	1.10E-04	4.00E-04	0.0036	3.50E-05	2.40E-04	4.80E-04	1.20E-04	3.90E-05	8.20E-04	0	1.30E-05	8.80E-04	2.20E-04	1.60E-04	0.0017	8.80E-07
NAGlySer 20:0;O(FA 28:1)	4.40E-04	0.021	0.0024	0.0061	8.20E-04	2.00E-05	8.40E-06	1.80E-05	7.20E-06	1.50E-05	3.00E-06	7.10E-05	1.20E-04	1.10E-04	1.50E-05	3.70E-04	5.20E-04	5.60E-04	3.40E-04	0.002	5.50E-07	0.0013	5.60E-05	3.10E-05	1.70E-06	2.10E-06	4.00E-04	1.30E-05	0	1.10E-06	1.50E-07	3.60E-06	5.20E-04	1.70E-06
Cer 16:1;O3/38:6(2OH)	0.0094	0.036	0.0021	0.017	0.0045	0.0025	1.10E-04	8.80E-04	6.10E-04	0.0024	2.90E-04	0.0061	0.017	0.021	0.0055	0.017	0.0094	0.0042	0.0052	0.0064	2.70E-05	0.012	0.0036	8.80E-04	9.80E-05	1.50E-04	0.022	8.80E-04	1.10E-06	0	9.10E-10	6.30E-05	4.40E-04	0.0013
Cer 18:2;O3/38:6(2OH)	0.018	0.052	0.003	0.017	0.0024	2.40E-04	6.10E-06	7.10E-05	5.00E-05	2.20E-04	1.10E-05	8.80E-04	0.0032	0.0042	0.0013	0.005	0.0038	0.0036	0.004	0.004	1.30E-05	0.0032	2.20E-04	1.50E-04	9.90E-06	4.30E-06	0.015	2.20E-04	1.50E-07	9.10E-10	0	9.90E-06	2.00E-04	1.80E-04
Cer 20:2;O3/38:6(2OH)	0.0013	0.1	0.0025	5.20E-04	0.0022	0.0027	2.00E-05	6.30E-05	3.50E-05	1.30E-05	2.00E-05	6.30E-05	3.10E-04	1.50E-04	3.10E-05	0.0017	2.20E-04	7.10E-05	3.70E-04	1.50E-04	5.10E-06	9.80E-05	4.30E-06	2.70E-05	4.00E-11	4.70E-10	7.90E-05	1.60E-04	3.60E-06	6.30E-05	9.90E-06	0	6.10E-04	3.60E-06
Cer 18:1;O2/27:1	0.0099	1.80E-04	0.0029	0.15	3.90E-05	0.003	0.0082	0.014	0.0094	0.0094	0.0055	0.017	0.028	0.0061	0.0032	6.50E-04	0.0074	0.002	0.0019	0.0019	0.0017	0.001	1.60E-04	1.10E-04	4.80E-04	4.00E-04	0.0052	0.0017	5.20E-04	4.40E-04	2.00E-04	6.10E-04	0	7.60E-04
SM 12:1;O2/35:0	2.40E-04	0.035	0.0014	0.0074	5.00E-05	1.10E-04	7.90E-05	8.40E-06	3.60E-06	1.90E-07	1.10E-05	1.10E-06	1.70E-06	7.80E-08	9.10E-10	1.70E-06	2.00E-04	3.10E-04	6.30E-05	1.10E-04	9.80E-05	6.10E-06	2.60E-07	8.40E-06	1.10E-06	4.30E-07	4.30E-06	8.80E-07	1.70E-06	0.0013	1.80E-04	3.60E-06	7.60E-04	0
